# Predicted T-Cell and B-Cell Epitopes of NIS: Where Do Sjögren’s Syndrome and Hashimoto’s Thyroiditis Converge?

**DOI:** 10.3390/ijms27010200

**Published:** 2025-12-24

**Authors:** Rossella Talotta, Gabriele Cammaroto, Rosaria Maddalena Ruggeri, Elisa Postorino, Salvatore Cannavò, Pasquale Aragona

**Affiliations:** 1 Rheumatology Unit, Department of Clinical and Experimental Medicine, University Hospital “G. Martino”, University of Messina, 98124 Messina, Italy; 2 Medicine and Surgery Bachelor Degree Course, Department of Human Pathology of Adulthood and Childhood DETEV “G. Barresi”, University Hospital “G. Martino”, University of Messina, 98124 Messina, Italy; gabriele.2003@outlook.it; 3 Endocrinology Unit, Department of Human Pathology of Adulthood and Childhood DETEV “G. Barresi”, University Hospital “G. Martino”, University of Messina, 98124 Messina, Italy; rmruggeri@unime.it (R.M.R.); cannavos@unime.it (S.C.); 4 Ophthalmology Clinic, Department of Biomedical Sciences, University Hospital “G. Martino”, University of Messina, 98124 Messina, Italy; elisapostorino@virgilio.it (E.P.); paragona@unime.it (P.A.)

**Keywords:** B-cell epitopes, computational analysis, Hashimoto’s thyroiditis, NIS, prediction models, Sjögren’s syndrome, T-cell epitopes

## Abstract

The sodium iodide symporter (NIS) is a key protein in thyroid function responsible for iodine uptake, and it may be involved in the pathogenesis of autoimmune thyroiditis. However, it is also expressed in the salivary glands, the primary target of autoreactive cells in Sjögren’s syndrome (SS). Given the common link between the two diseases, we computationally investigated whether the epitopes of NIS can trigger an immune response leading to SS in Hashimoto’s thyroiditis (HT) patients genetically predisposed to both diseases. The TepiTool 2016, ABCpred 2006, and DiscoTope 2.0 servers were used to predict T-cell and B-cell epitopes by inputting the FASTA sequences and 3D structures of NIS, thyroid peroxidase (TPO) and Ro60 Y RNA-binding protein (Ro60), which served as reference antigens for HT and SS, respectively. T-cell epitopes were selected based on their binding to a panel of human leukocyte antigen (HLA) alleles associated with both SS and HT. We identified a total of 376 linear T-cell epitopes, 64 linear B-cell epitopes and 68 conformational B-cell epitopes of NIS. Compared to TPO, NIS T-cell epitopes showed significantly lower affinity for HLA alleles (*p* < 0.0001), while no significant difference was found compared to Ro60. While linear B-cell epitopes of NIS, TPO, and Ro60 showed similar binding affinity, conformational epitopes of NIS were predicted to have higher immunogenicity than Ro60 (*p* = 0.04), while no significant difference was found compared to TPO. These pivotal findings, discovered by the methods of computer modeling, suggest that NIS can potentially activate T cells and B cells in patients with genetic predisposition to SS and HT and need to be confirmed by further laboratory studies.

## 1. Introduction

Sjögren’s syndrome (SS) is a systemic autoimmune disease mainly affecting exocrine glands, particularly salivary and lacrimal glands, causing xerostomia and xerophthalmia in most patients [1,2]. Other symptoms include dysphagia, oral burning, gland swelling, and eye complications. Dysfunction of other exocrine glands can cause vaginal dryness, hoarseness, cough, pancreatitis, and gastritis [1]. Extraglandular involvement may affect skin, joints, lungs, heart, nerves, kidneys, and gastrointestinal tract, leading to arthritis, cutaneous rashes, interstitial lung disease, arrhythmias, neuropathies, and, rarely, nephritis or glomerulonephritis [1].

The prevalence of SS varies between 0.013% and 3.5%, depending on the populations studied [3]. The disease mainly affects women, with a female-to-male ratio of 9–20:1. SS can occur alone or be associated with other diseases such as rheumatoid arthritis (RA), systemic lupus erythematosus (SLE) and myositis [4,5,6]. In addition, SS can occur in patients with Hashimoto’s thyroiditis (HT) with a tenfold higher risk compared to the general population [7]. HT is the most common autoimmune thyroid disease, characterized by T-lymphocyte infiltration and follicular destruction [8]. It affects 0.3–1.5 per 1000 people yearly [9] with a 7.5% adult prevalence [10]. Similar to SS, the disease is more common in women with a female-to-male ratio of 7–10:1 [11].

SS occurs in 3–32% of patients with autoimmune thyroiditis [7]. This correlation between the two diseases can be attributed to a common genetic predisposition, shared environmental factors and pathogenetic mechanisms, as well as the possible presence of antigens expressed in both the thyroid and salivary glands. Regarding genetic predisposition, SS and HT patients may carry some polymorphic variants of genes belonging to the human leukocyte antigen (HLA) locus, such as the alleles HLA-DR3, HLA-DRB1*04:05, HLA-DR8, HLA-DR11, HLA-DRB4*01:01, HLA-DQA1*05:01 and HLA-DQB1*02:01 [12,13,14,15]. The two diseases also show some similarities in terms of pathogenetic mechanisms. Indeed, in HT and SS, the damage to the thyroid and salivary glands is caused by the activation of an immunological cascade triggered by cytotoxic CD8+ T lymphocytes through the release of interferon-gamma (IFN-γ) and tumor necrosis factor-alpha (TNF-α) and the activation of the FAS/FAS ligand pathway [7]. This is followed by an intervention in the humoral response with the activation and differentiation of B lymphocytes into plasma cells and the production of autoantibodies, which is partly due to the increase in B-cell activating factor (BAFF) levels. Thus, both diseases are characterized by hyperactivation of B lymphocytes, which may directly interact with solvent-exposed antigens through B-cell receptors (BCRs) and become activated regardless of HLA-mediated antigen presentation [16]. Anti-Ro/SSA and anti-La/SSB antibodies are the two most peculiar antibodies for the diagnosis of SS, being found in 50–90% and 30–60% of patients with SS, respectively [17]. HT is diagnosed through anti-thyroid peroxidase (TPO) and anti-thyroglobulin (Tg) antibodies, while the role of anti-pendrin and anti-sodium iodide symporter (NIS) antibodies is still debated [18].

It is known that NIS plays an antigenic role in the development of HT, even if it is marginal compared to TPO and Tg. Indeed, while anti-TPO and anti-Tg antibodies are positive in 95% and 60–80% of patients with HT, respectively [19], anti-NIS antibodies are present in only 27% of cases according to a study [20]. Since the latter are not frequently found, their dosage is generally only required for research purposes as it has little diagnostic value [18]. However, NIS is present in both follicular thyroid cells and salivary glands [21], which may explain immune cross-reactivity in patients with both HT and SS. While NIS involvement in thyroid autoimmunity is documented [7,22,23], its role in SS remains unclear and no prior epitope-level comparative analysis of NIS in SS has been reported in the literature.

Therefore, we conducted a pilot hypothesis-generating study whose main objective was to identify, by computational analysis methods, the presence of T-cell and B-cell epitopes of NIS capable of determining the development of SS in genetically predisposed patients to both SS and HT.

The secondary objectives of the study were to evaluate the degree of immunogenicity of the T-cell and B-cell epitopes of NIS compared with TPO and ribonucleoprotein Ro60 Y RNA-binding protein (Ro60), which represent the main antigenic targets in HT and SS, respectively.

## 2. Results

### 2.1. Linear T-Cell Epitopes of NIS

Analysis with the TepiTool server revealed a total of 376 linear T-cell epitopes of NIS that may bind to HLA class II alleles associated with SS and HT (Table 1 and Table S1). Thirty-one of these had a percentile rank ≤ 0.5, which corresponds to a high affinity for the selected alleles. The epitope with the highest binding affinity (percentile rank 0.01–0.03) for the alleles HLA-DRB1*03:05, HLA-DRB1*03:14 and HLA-DRB1*03:40 was NLMDFNPDPRSRYTF, which is located on the extracellular side of the protein between the highly glycosylated transmembrane domains VI and VII according to the Uniprot.org database [24]. The other two peptides with the lowest percentile ranks were PRKLVIISKGLSLIY and EDLIKPRLRSLAPRK, located in the cytosolic and cytosolic-transmembrane domains, respectively [24]. The mean ± standard deviation (SD) of the percentile rank of the NIS T-cell epitopes to the selected HLA alleles was 5.04 ± 3.09.

### 2.2. Linear B-Cell Epitopes of NIS

The ABCpred analysis predicted a total of 64 linear B-cell epitopes of NIS with a mean ± SD score of 0.75 ± 0.11 (Table 2). Four epitopes achieved a score ≥ 0.90; these were AGSWTPCVGHDGGRDQ (localized at the highly phosphorylated intracellular carboxyterminal end), LGRISAPDQYMPLLVL (localized extracellularly between the VIII and IX transmembrane domains), MEAVETGERPTFGAWD (localized at the extracellular amino-terminal end), and GVLQGSFTVMGVISGP (localized extracellularly between the X and XI transmembrane domains) [24].

### 2.3. Conformational B-Cell Epitopes of NIS

**Figure 1:**
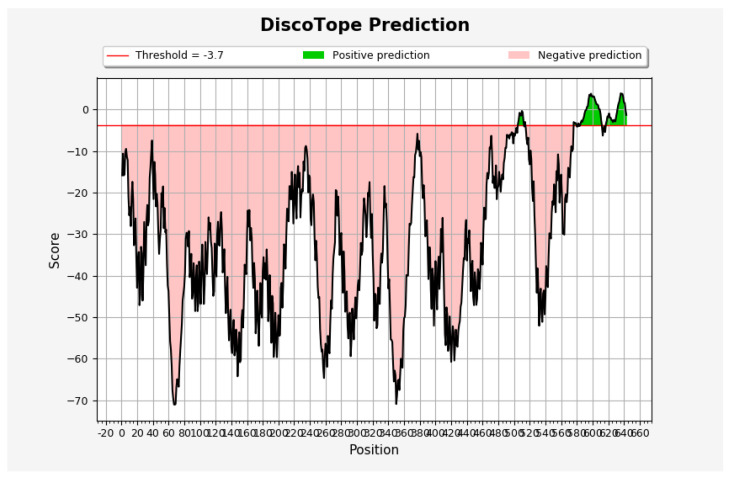
Graphical representation of positive predictions of NIS conformational B-cell epitopes (highlighted in green). A DiscoTope score above the threshold value of −3.7 corresponds to positive predictions, while a score below this threshold indicates negative predictions.

**Figure 2:**
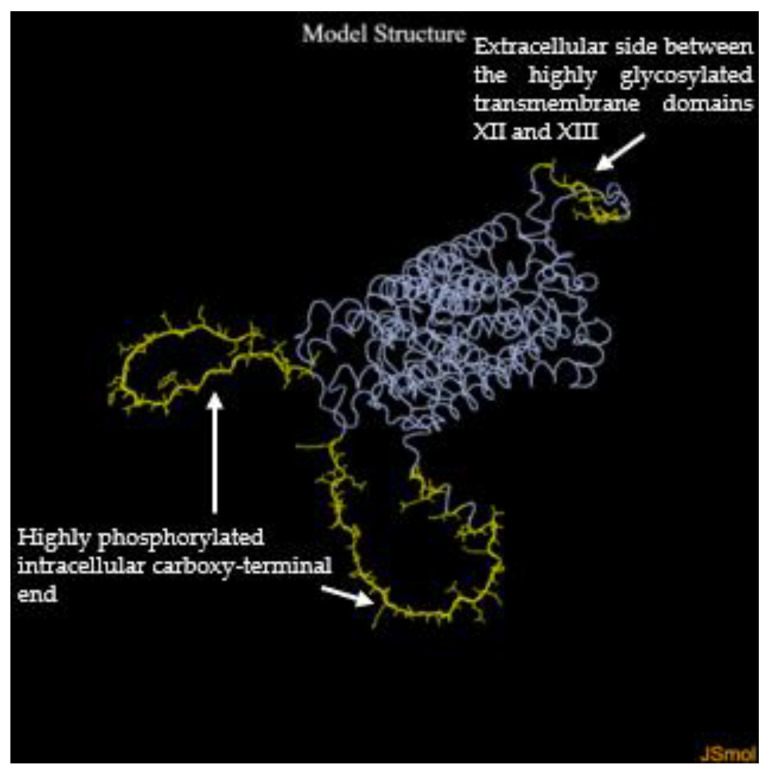
Representation of the conformational B-cell epitopes of NIS, highlighted in yellow in the 3D structure of the protein. White arrows indicate their positions within the protein domains.

DiscoTope analysis of the NIS 3D structure, which has a single α-chain, revealed a total of 68 conformational B-cell epitopes with a DiscoTope score ranging from 3.941 to −3.486, Table 3 and Figure 1 and Figure 2. The median (interquartile range, IQ) score was −0.787 (4.302). According to the Uniprot.org database [24], the epitopes are located on the extracellular side of the protein between the highly glycosylated transmembrane domains XII and XIII, specifically at amino acid positions 505–512 and 514, whereas the epitopes involving the amino acid residues between 576 and 579, in position 582, between 585 and 611, and between 617 and 643 are located at the highly phosphorylated intracellular carboxy-terminal end. No positive prediction was found for the amino acids of the transmembrane domains.

### 2.4. Comparative Analysis of T-Cell and B-Cell NIS Epitopes Versus TPO and Ro60

#### 2.4.1. Linear T-Cell Epitopes of TPO

When TepiTool analysis was applied to TPO, a total of 822 linear T-cell epitopes were found to bind to HLA class II alleles associated with SS and HT. Sixty-seven of these had a percentile rank ≤ 0.5, Table S2. The epitope with the highest binding affinity (percentile rank 0.03) to the HLA-DRB1*08:10 and HLA-DRB1*08:12 alleles was PAQLLSFSKLPEPTS, which is placed extracellularly according to the Uniprot.org database [24]. The mean ± SD of the percentile rank scores was 4.30 ± 2.96. Student’s *t*-test for unpaired samples revealed a significantly lower binding affinity for linear T-cell epitopes of NIS than for those of TPO (*p* < 0.0001).

#### 2.4.2. Linear B-Cell Epitopes of TPO

ABCpred analysis predicted a total of 93 linear B-cell epitopes for TPO. Fourteen epitopes, all located on the extracellular part of the protein according to the Uniprot.org database [24], achieved a score ≥ 0.90, Table S3. Overall, the mean ± SD of the ABCpred score was 0.78 ± 0.10. Statistical analysis showed a trend towards a lower prediction of BCR-binding for NIS compared to TPO (*p* = 0.05, Student’s *t*-test for unpaired samples).

#### 2.4.3. Conformational B-Cell Epitopes of TPO

The 3D structure of TPO was predicted to contain 101 conformational B-cell epitopes in the α-chain, with a DiscoTope score ranging from 3.456 to −3.682. The positive predictions are shown in Table S4 and Figure S1. The median (IQ) of the DiscoTope score for TPO was −2.129 (2.268). Using the Mann–Whitney U test for unpaired samples, no significant difference was found between NIS and TPO scores (*p* > 0.05). According to the database Uniprot.org [24], positive predictions involving amino acids between 30 and 33, at position 103, between 105 and 115, at position 117, between 181 and 184, between 192 and 201, at positions 382, 383, 469, 471, 472, 475, 476, 512, 627, 680, 738, 739 and 784 were located in the extracellular domain, while those containing amino acid residues between 878 and 933 were placed in the cytoplasmic domain. No positive predictions were found for amino acids in the transmembrane domain.

#### 2.4.4. Linear T-Cell Epitopes of Ro60

Using TepiTool analysis, a total of 686 linear T-cell epitopes of Ro60 were predicted to bind HLA class II alleles associated with SS and HT, with a mean ± SD percentile rank of 5.06 ± 2.99. Twenty-three of these had a percentile rank ≤ 0.5, Table S5. The epitope with the highest binding affinity (percentile rank 0.19 for the HLA-DRB1*08:05 allele) was DAAFYKTFKTVEPTG, which is located in the TAR RNA-binding protein and oxidative stress response-related (TROVE) domain according to the Uniprot.org database [24]. No significant difference was found between the binding avidity of Ro60 and NIS epitopes to selected HLA alleles (*p* > 0.05, Student’s *t*-test for unpaired samples).

#### 2.4.5. Linear B-Cell Epitopes of Ro60

The ABCpred analysis predicted a total of 53 linear B-cell epitopes of Ro60 (Table S6). Two epitopes scored ≥0.90; these were located in the von Willebrand Factor A(VWFA)-like domain according to the Uniprot.org database [24] and were VHPAIALREYRKKMDI and ASTVAAAMCMVVTRTE. The mean ± SD ABCpred score of Ro60 epitopes was 0.75 ± 0.09. There were no statistically significant differences when comparing NIS and Ro60 (*p* > 0.05, Student’s *t*-test for unpaired samples).

#### 2.4.6. Conformational B-Cell Epitopes of Ro60

According to DiscoTope analysis, the 3D structure of Ro60 contains 18 conformational B epitopes in the α-chain, whose score ranges from 1.674 to −3.598 (median [IQ] 2.129 [2.268]), Table S7, Figure S2. According to the Uniprot.org database [24], the positive predictions involved the cytoplasmic domain (amino acids at positions 1–5, 9–10, 12–14), the TROVE domain (amino acids at positions 21, 239, 240, 338–340) and the VWFA-like domain (amino acids at positions 488 and 491). The Mann–Whitney U test for unpaired samples showed a significantly higher binding prediction for NIS than for Ro60 (*p* = 0.04).

#### 2.4.7. HLA Class II Binding of NIS, TPO and Ro60 T-Cell Epitopes

The T-cell epitopes of NIS, TPO and Ro60 could be presented by the same polymorphic variants of the HLA class II complex, although with variable degrees of binding affinity, as reported in Table 4.

The table shows that the HLA-DRB1*11:06 allele has the highest average affinity for NIS, TPO and Ro60. This allele is associated with an increased risk of developing SS in the Spanish population [12] and HT in the Indian population [14]. Although the association between HLA-DR11 and the formation of anti-La/SSB antibodies in SS is not yet well defined, a study conducted in the Taiwanese population suggests a possible role that requires further investigation [13].

## 3. Discussion

The results of this computational study suggest that NIS may contain epitopes for T and B cells that could potentially trigger the onset of SS in patients with genetic predisposition to SS and HT. According to these data, NIS could act as a common antigen for autoreactive cells and antibodies active in HT and SS, justifying the non-negligible overlap rate between the two diseases [7].

NIS is a glycosylated hydrophobic phosphoprotein of 643 amino acids, consisting of 13 transmembrane segments, with a highly phosphorylated intracellular carboxyterminal end [21]. Biologically, NIS mediates the active transport of iodine with a stoichiometric sodium-to-iodine ratio of 2:1 due to the electrochemical sodium gradient generated by the Na+/K+ ATPase in the thyroid gland and other tissues such as the salivary glands, stomach, small intestine and breast during lactation. NIS can also promote the transport of other anions such as selenium, cyanate, thiocyanate, chlorate and nitrate [21].

In the thyroid gland, NIS is located on the basolateral membrane of the thyrocytes and actively transports iodine from the bloodstream, concentrating it intracellularly. The iodine then passes through the apical membrane into the lumen of the follicle, where it undergoes a series of enzymatic steps to be incorporated into Tg at the level of tyrosine residues [21,25].

In the salivary glands, NIS is placed on the basolateral side of the epithelial cells of the salivary ducts and allows the entry of iodine, which is subsequently secreted through the saliva, where it carries out an antimicrobial action [21]. Immunohistochemical studies have shown that NIS is more highly expressed in the basolateral membranes of the cells of the striated ducts than in the cells of the intercalated and excretory ducts, regardless of the type of gland [26]. Acinar cells do not express NIS. Also, NIS is more highly expressed in the parotid gland than in the submandibular glands and even more so in the minor salivary glands.

In the thyroid, the expression and activity of NIS are regulated by several mechanisms, such as the hypothalamic-pituitary-thyroid axis, the Wolff-Chaikoff effect, the maintenance of thyrocyte membrane potential by the voltage-gated K channels KCNQ1 and KCNE2, and the epigenetic regulation of the *SLC5A5* gene [21,27]. Other factors that could additionally influence the expression of NIS are cytokines and growth factors, such as insulin and insulin-like growth factor-1 (IGF-1), transforming growth factor-beta1 (TGF-β1), TNF-α, IFN-γ, interleukin-1 alpha (IL-1α) and interleukin-1beta (IL-1β), interleukin-6 (IL-6) and 3-iodothyronamine (3-T1AM) [25]. In particular, the expression of pro-inflammatory cytokines is associated with reduced transcription of NIS mRNA in in vitro studies in thyrocytes [28]. Translocation of NIS to the basolateral membrane is also regulated by post-translational modifications such as glycosylation and phosphorylation, which may play a crucial role in the generation and exposure of potential conformational epitopes [27].

In salivary glands, the factors influencing the expression of NIS are less clear. Indeed, its expression is not homogeneous, but varies depending on a series of factors, such as the type of gland, the portion of the glandular parenchyma examined and the presence or absence of an ongoing pathological process [26].

Several studies have confirmed the antigenic role of NIS in the development of HT, although marginal compared to TPO. Anti-NIS antibodies have been described in patients with autoimmune thyroiditis [18,19,20,29,30,31,32,33] including HT, but their clinical role is not entirely clear. By binding to NIS, such antibodies could interfere with the internalization of iodine, leading to hypothyroidism, which may follow HT diagnosis. However, in patients with autoimmune thyroiditis, percentages of anti-NIS antibodies are lower than those of anti-TPO and Tg antibodies. These laboratory findings are confirmed by histological studies that show that patients with HT show weak or even absent NIS mRNA expression in the thyroid gland [26]. Similarly, NIS expression in the striated ducts is reduced in inflammatory diseases of the salivary glands and even more so in benign and malignant neoplasms of ductal origin [26]. This could be partly explained by the increase in pro-inflammatory cytokine burden, such as TNF-α, IL-1 and IFN-γ [18,34]. It is important to underline that the inflammatory scenario that takes place in the thyroid gland during HT is in many ways reminiscent of what happens in the exocrine glands of SS patients.

**Figure 3:**
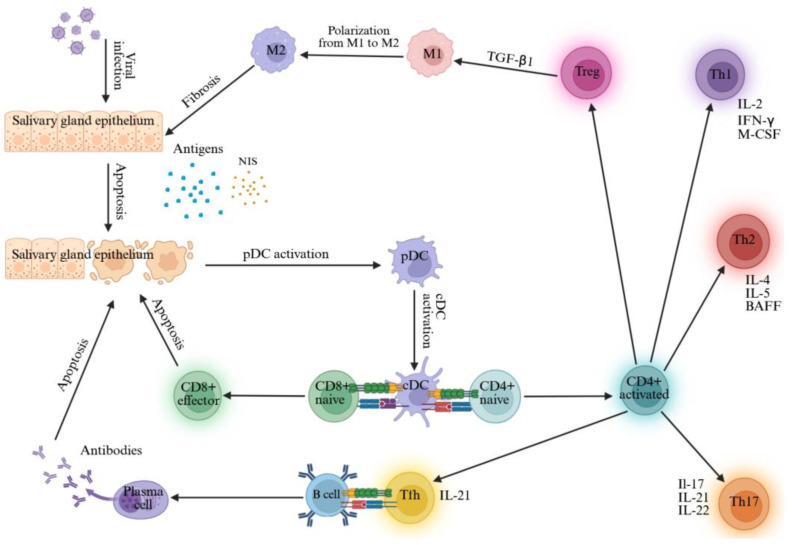
Immunopathogenesis of SS. The development of SS in genetically predisposed individuals appears to be triggered by external stimuli (e.g., viral infections) that cause cellular apoptosis in the salivary glands, resulting in a massive release of antigens, including NIS, that are scavenged by the pDCs. The pDCs activate the cDCs, which present the antigens and activate naïve CD4+ and CD8+ T cells. The latter are responsible for direct cellular damage via the apoptotic pathway. CD4+ T cells may instead differentiate into Th1, Th2, Th17, Tfh and Treg cells. The B cells act as APCs by capturing the autoantigens released after apoptosis of the salivary gland epithelial cells, presenting them to the Tfh cells and differentiating into plasma cells that produce autoantibodies. Treg cells produce TGF-β1, which polarizes macrophages from a pro-inflammatory phenotype (M1) to an anti-inflammatory phenotype (M2) and stimulates fibrosis. Abbreviations: APC, antigen-presenting cell; BAFF, B-cell activating factor; CD, cluster of differentiation; cDC, classical dendritic cell; IFN, interferon; IL, interleukin; M-CSF, macrophage colony-stimulating factor; NIS, sodium-iodine symporter; pDC, plasmacytoid dendritic cell; Tfh, T follicular helper cell; TGF-β1, transforming growth factor-β1; Th, T helper cell; Treg, T regulatory cell. Created with BioRender.com.

The pathogenesis of SS and HT, which is associated with a complex interplay of genetic, epigenetic and environmental factors [4,12,13,35,36,37,38,39,40,41], centers on abnormal activation of the innate and acquired immune system with activation of plasmacytoid dendritic cells (pDC) via Toll-like receptors (TLR) 7 and 9 and classical dendritic cells (cDC), and subsequently of CD8+ and CD4+ T lymphocytes, of which the latter differentiate into T helper 1 (Th1) cells, Th2 cells, Th17 cells, regulatory T cells (Treg) and T follicular helper cells (Tfh), with activation of B lymphocytes and plasma cells, hypergammaglobulinemia and antibody production [42,43]. In the advanced stage of the disease, the persistence of this chronic inflammatory pattern can lead to fibrosis and glandular atrophy, thanks to the action of TGF-β1, a pro-fibrotic cytokine that regulates the epithelial–mesenchymal transition process leading to fibrosis via the canonical TGF-β1/SMAD/Snail signaling pathway [42,44,45], Figure 3.

Similarly, HT is characterized by immunopathogenesis dominated by the proliferation of T lymphocytes, including CD4+ Th1 cells, which produce IFN-γ, IL-2 and TNF-α that activate cytotoxic lymphocytes and macrophages and cause the destruction of thyroid follicles [46,47]. In the subsequent phases, in addition to the cell-mediated damage, the humoral response also intervenes, thanks to the action of BAFF, which causes hyperactivation and differentiation of the B-cell lineage into plasma cells with the production of antibodies against TPO, Tg, thyroid-stimulating hormone receptor (TSH-R), pendrin and NIS. The increased expression of Th1 lymphocytes is accompanied by a Th17/Treg imbalance that causes the destruction of thyroid cells, which can lead to a state of transient thyrotoxicosis due to the massive release of thyroid hormones. This can lead to long-term hypothyroidism, which is associated with progressive damage to the parenchyma [10].

**Figure 4:**
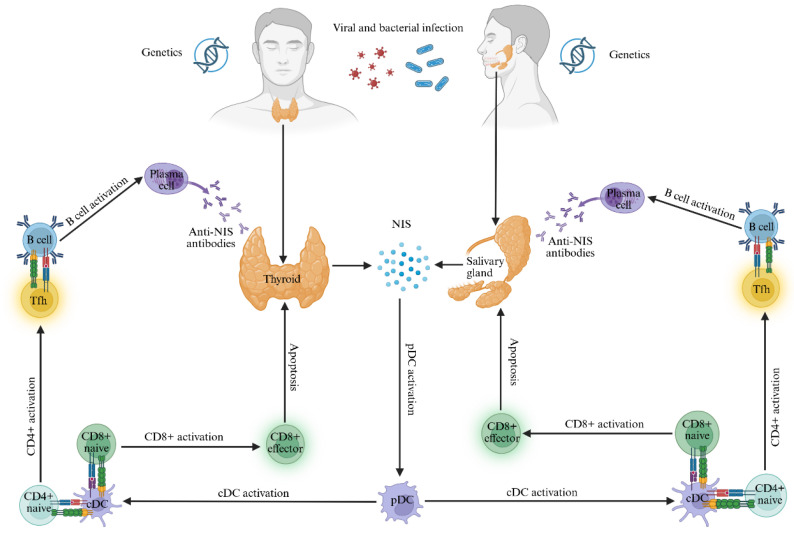
Hypothetical immunopathogenetic mechanism by which NIS could induce the occurrence of SS in patients with HT. The development of SS in genetically predisposed individuals with HT could be triggered by an environmental event, such as infections caused by viral and bacterial pathogens that share homologous amino acid sequences with NIS. Apoptosis of infected cells may lead to an anomalous process of NIS release, with subsequent phagocytosis by APCs, processing and antigen presentation in association with specific polymorphic variants of HLA class II molecules to naive T lymphocytes. This process could be enhanced by the simultaneous recognition of the conformational epitopes of NIS by B lymphocytes via the BCR, with subsequent digestion and presentation of the epitopes to Tfh lymphocytes, which assist their differentiation into plasma cells. The latter could produce anti-NIS autoantibodies, responsible, at a later stage, for a chronic inflammatory autoimmune process of the salivary glands and the thyroid. Abbreviations: CD, cluster of differentiation; cDC, classical dendritic cells; NIS, sodium-iodine symporter; pDC, plasmacytoid dendritic cells; Tfh, T follicular helper cells. Created with BioRender.com.

In both immunopathogenic scenarios, apoptosis of salivary and thyroid cells could induce the release of antigens such as NIS, which can elicit a cross-reactive immune response. This may be due to a common genetic predisposition, since SS and HT share some polymorphic variants of the HLA genes [12,13,14,15] and non-HLA genes [48] and similar pathogenic mechanisms [7,39,40,41]. Being expressed in both thyroid and salivary glands, NIS could be a common target of T and B autoreactive cells with the development of a synergic immune response. Specifically, it could be hypothesized that NIS acts as a trigger factor at the beginning of both diseases, while in the advanced stages it constitutes the target, which would reduce its expression as already observed in the experimental studies by Krista MD La Perle et al. [26], Figure 4.

Although studies on the prevalence of anti-NIS antibodies in SS patients are lacking, our computational analysis suggests that NIS may potentially contain immunogenic epitopes that could justify the coexistence of both diseases in these patients. Interestingly, NIS T-cell and B-cell epitopes scored overall lower compared to TPO and thus appeared to be less immunogenic, while they were comparable or slightly more immunogenic than Ro60. In detail, the linear T-cell and B-cell epitopes of NIS showed a significantly lower immunogenic potential than those of TPO, while they did not statistically differ compared to Ro60. Conversely, the 3D protein structure of NIS would contain several B-conformational epitopes with a DiscoTope score significantly higher than the epitopes of Ro60, while there would be no significant differences compared to TPO. Therefore, it could be hypothesized that NIS may have a greater pathogenetic role in inducing SS, compared to the reference antigen Ro60, but would play a secondary function in the pathogenetic scenario of HT. However, it is important to note that lower predicted affinity does not necessarily imply limited pathogenic relevance, particularly in chronic autoimmune conditions [49]. Regarding the NIS linear T-cell epitopes, our analysis revealed that the peptide NLMDFNPDPRSRYTF was the one with the highest binding affinity (percentile rank 0.01–0.03) for the HLA-DRB1*03:05, HLA-DRB1*03:14 and HLA-DRB1*03:40 alleles, which, according to the literature, are those most associated with the risk of developing SS and HT in the Spanish population [14,50,51]. According to the Uniprot.org database [24], this epitope is located on the extracellular side, between transmembrane domains VI and VII, where one of the three glycosylation sites of NIS is contained [21]. As reported in other studies, glycosylation is a post-translational process that significantly affects the immunogenic potential of various antigens and plays a key role in the activation of the autoimmune response [52]. In addition to this site, NIS contains two other glycosylation sites located in the loop between transmembrane domains XII and XIII [21], which also appear to include T-cell and B-cell computational epitopes according to our analysis. It is important to emphasize that the epitopes at these sites were confirmed by an in vitro flow cytometry study and a radiobinding assay study [53,54]. However, the NIS epitopes found in laboratory studies are partially consistent with those found in our research.

NIS is not only a highly glycosylated protein, but also has a dimeric conformation stabilized by a disulfide bridge, which is essential for its function [55,56,57]. Several in vitro studies showed that amino acid residues such as Ser-66, Glu-79, Arg-82, Lys-86, Asp-163, Asp-191, Gln-194, His-226, Arg-228, Asp-233, Asp-237, Arg-239, Arg-241, Gln-263, Asp-311, Asp-322, Asp-331, Ser-353, Thr-354, Asn-441, Tyr-242, Thr-243, and Gln-471 play a key role in the dimerization process and the functional regulation of iodine transport activity [58,59,60,61,62]. Our computational research revealed that such amino acid residues are placed within the predicted T-cell and B-cell epitopes. In particular, the peptide NLMDFNPDPRSRYTF has some amino acid residues (Tyr-242, Thr-243) that are involved in the dimerization of NIS [62] and in the control of iodine transport (Asp-233, Asp-237, Arg-239 and Arg-241) [58]. It can therefore be assumed that epitopes affecting these amino acid positions could impair the folding process and iodine transport activity, which in turn affects the synthesis of thyroid hormones or the antimicrobial function of NIS in the salivary glands [21].

This study did not detect homologous amino acid sequences between the predicted epitopes in NIS and those in TPO or Ro60. We could therefore exclude a direct antigenic cross-reactivity between NIS, TPO and Ro60. However, according to an in silico study, NIS and TPO share homologous amino acid sequences with certain Borrelia and Yersinia proteins, including amino acid residues located at the binding motifs of the T-cell receptor (TCR) and several polymorphic variants of the HLA-DR locus (HLA-DR3, HLA-DR4, HLA-DR5, HLA-DR8, and HLA-DR9), some of which predispose not only to HT, but also to SS [63]. Therefore, it could be hypothesized that pathogens such as Borrelia or Yersinia may be the triggering factor that determines the hyperactivation of the immune system in genetically predisposed individuals through molecular mimicry mechanisms towards autoantigens such as TPO or NIS. Importantly, our analysis has shown that the epitopes of NIS, TPO and Ro60 can be presented by the same polymorphic variants of the HLA class II complex, albeit with different degrees of binding affinity.

Although providing a new perspective in deciphering the immunopathogenic scenario underneath SS and HT, this pivotal study has several limitations.

Indeed, the main limit is due to the computational nature and the current availability of data in the biobanks and databases consulted. For example, for the search for T-cell epitopes, the TepiTool server uses the Immune Epitope Database (IEDB), which is continuously updated with the insertion of new data from experimental studies on humans and other species, so in the future it may be possible to predict additional epitopes capable of binding the same HLA alleles [64]. Furthermore, for the analysis of linear T-cell epitopes, we referred to HLA class II alleles whose association with SS and HT is already described in the literature in different ethnic groups; however, the selected alleles may not represent the entire polymorphic panorama of HLA in the world population due to the lack of studies conducted in different ethnic groups, especially in the African continent for which we have no data available. Furthermore, our search for linear T-cell epitopes was limited to the panel of HLA class II alleles available in the IEDB, so it was not possible to test the binding affinity of other HLA alleles predisposing to the two diseases [65].

The prediction of B-cell epitopes is further conditioned by both the mechanisms of protein folding and the conformational structures that condition the accessibility to antibodies capable of binding a specific epitope [16], by the flexibility of the epitopes, and by the difficulty in mapping the link between paratope and epitope [66]. It should be added that the computational predictions do not take into account changes in protein conformation, charge, pH, or interaction with proteins other than antibodies [67]. As already mentioned, NIS contains three glycosylation sites and its expression can be tuned by a variety of factors, including pro-inflammatory cytokines. Finally, the methods used to predict B-cell epitopes are based on neural network systems and propensity scales, which are characterized by modest overall accuracy and sensitivity [68,69].

Therefore, further laboratory studies are needed to evaluate the immunogenic potential of the in silico predicted NIS epitopes, using flow cytometry, enzyme-linked immunosorbent assay (ELISA), phage display libraries, peptide microarrays or T-cell functional assays [66]. Specifically, it would be advisable to compare the immunogenicity of predicted epitopes with that of peptides naturally processed from the original protein by antigen presenting cells (APCs); characterize the stable, long-lasting interaction of predicted epitopes with the HLA class II alleles studied; measure the effective presentation to T cells and the elicited T-cell response; confirm the static and dynamic structure of conformational epitopes; test the downstream immunologic cascades, including changes in gene expression; and assess the magnitude of the antibody response. Predicted NIS epitopes that are extracellular and computationally show higher avidity for HLA or BCRs, as well as those likely to be presented by HLA alleles common in the population and clearly associated with both diseases across ethnic groups, should be prioritized for experimental validation and antigenicity testing. According to our analysis, these include the T-cell epitope NLMDFNPDPRSRYTF, the B-cell linear epitope LGRISAPDQYMPLLVL, and conformational epitopes spanning amino acid positions 505 to 512 and at position 514. However, since NIS may be released during cell apoptosis, intracellular or transmembrane cryptic epitopes could also contribute to eliciting an aberrant immune response.

As mentioned above, two existing epitope mapping studies found NIS epitopes spanning multiple amino acid stretches that partially matched those found in our analysis. Similarly, the epitopes found in the reference antigens TPO and Ro60 partially coincided with those found in previous laboratory studies. Specifically, our research identified epitopes located in the Immune Domain Region (IDR)-A and IDR-B, Myeloperoxidase (MPO)-like and Complement Control Protein (CCP)-like modules of TPO and in the TROVE domain and in the VWFA-like domain of Ro60 that were also confirmed by flow cytometry, ELISA, phage display technology and mutagenesis studies, Western blot, and counterimmunoelectrophoresis [18,70,71,72,73,74,75,76,77,78,79,80,81,82,83,84]. However, it is also important to characterize the potential immunological mechanisms that could arise from the recognition of these peptides, such as peripheral tolerance phenomena, cross-reactivity and epitope spreading.

Finally, another limitation and a possible future research point is the evaluation of the presence of anti-NIS antibodies in other rheumatic and non-rheumatic autoimmune diseases known to be associated with HT, based on a common genetic predisposition [14,85].

## 4. Materials and Methods

### 4.1. HLA Allele Selection

Although studies have described the association between SS and HLA class I alleles [86] and between HT and HLA class I thyroid tissue expression [87], we intentionally focused on HLA class II predisposing variants, as they are linked to the humoral response and the production of autoantibodies rather than a direct cytotoxic response. Their association with both diseases is also supported by more robust scientific evidence [12,50].

To select the HLA class II alleles predisposing to both SS and HT to be used in the subsequent analyses, a review of the medical literature was performed by searching the PubMed database (National Library of Medicine, Bethesda, MD, USA) for relevant articles in the different ethnic groups. The search was completed on 13 December 2024 and resulted in the selection of 16 and 21 allelic polymorphisms, associated with the risk of developing SS and HT in the different ethnic groups, Table 5 [12,13,50,88,89,90,91,92,93,94,95,96,97,98,99,100] and Table 6 [14,15,51,101,102,103,104,105,106,107,108,109,110,111,112,113,114,115,116,117,118,119,120,121]. For every single allelic polymorphism, the frequency in each population studied is reported according to the Allele Frequencies Net database (Transplant Immunology Royal Liverpool University Hospital, Liverpool, UK; https://www.allelefrequencies.net, accessed on 13 May 2025) [122].

Of these alleles, 50 subtypes available in the IEDB dataset were associated with both SS and HT predisposition (Table 7) and were therefore selected to begin the search for linear T-cell epitopes according to the methods described in the next section.

### 4.2. Predictive Analysis of Linear T-Cell Epitopes

To predict the linear T-cell epitopes of NIS, the FASTA protein sequence was searched on the NCBI website (https://www.ncbi.nlm.nih.gov/protein/Q92911.1, accessed on 25 March 2025). This sequence was inserted as input into the TepiTool server provided by the IEDB (La Jolla Institute for Immunology, La Jolla, CA, USA; https://tools.iedb.org/tepitool/ accessed on 25 March 2025) [64,65]. The IEDB is a continuously updated database of over 1.6 million epitopes related to autoimmune, infectious and allergic diseases and transplant rejection that have been studied in humans, non-human primates and other animal species [65]. In addition, it provides advanced tools for the prediction of T-cell epitopes, such as the TepiTool, which makes it possible to identify the regions of a protein that the immune system might recognize by predicting binding to HLA class I and II molecules [64]. The standard IEDB analysis uses the consensus method, which has an estimated area under the ROC curve (AUC) of 0.89 ± 0.05 and a sensitivity in predicting T-cell activation of 66.7% [123]. For our analysis, we used the default settings suggested by the server for a moderate number of epitopes with a length of 15 amino acids and a percentile rank of less than 10. Duplicates were removed and the peptide overlap was set to 10 amino acids. The search for linear T-cell epitopes was performed with respect to the binding of the HLA class II alleles that predispose to SS and HT and are already listed in Table 7.

The same methods were used to analyze the affinity of the HLA class II alleles listed in Table 7 for epitopes within the entire FASTA protein sequence of human TPO (https://www.ncbi.nlm.nih.gov/protein/KAI4033394.1, accessed on 25 March 2025) and human ribonucleoprotein Ro60 (https://www.ncbi.nlm.nih.gov/protein/KAI4084282.1, accessed on 25 March 2025).

### 4.3. Predictive Analysis of Linear B-Cell Epitopes

The bioinformatics tool ABCpred (Department of Computational Biology, Indraprastha Institute of Information Technology, New Delhi, India; https://webs.iiitd.edu.in/raghava/abcpred/ accessed on 25 March 2025) was used to predict linear B-cell epitopes [68,124]. ABCpred is an artificial neural network-based server capable of estimating the affinity of peptides for BCRs with an accuracy of 65.93% and equal sensitivity and specificity, excluding identical epitopes or non-immunogenic peptides [66]. In our study, the FASTA protein sequence of NIS (https://www.ncbi.nlm.nih.gov/protein/Q92911.1, accessed on 25 March 2025) was entered into the search box to start the analysis using the suggested default settings (threshold 0.51; no overlap filter; prediction window length: 16). Using the same methods, we searched for linear B-cell epitopes within the entire FASTA protein sequence of human TPO (https://www.ncbi.nlm.nih.gov/protein/KAI4033394.1, accessed on 25 March 2025) and Ro60 (https://www.ncbi.nlm.nih.gov/protein/KAI4084282.1, accessed on 25 March 2025).

### 4.4. Predictive Analysis of Conformational B-Cell Epitopes

To predict the conformational B-cell epitopes, the 3D protein structure of NIS was searched on the AlphaFold Protein Structure Database website (EMBL-EBI, Wellcome Genome Campus, Hinxton, Cambridgeshire, UK; https://alphafold.ebi.ac.uk/entry/Q92911, accessed on 25 March 2025) [125]. AlphaFold Protein Structure Database is a digital library containing over 214 million 3D protein structures predicted with high accuracy by the AlphaFold 2 artificial intelligence system. Each protein model contains atomic coordinates and confidence scores for each residue called pLDDT, which range from 0 to 100. High scores (≥90) indicate high confidence of the predicted structure [125].

The search returned the NIS protein identifier (Q92911), whose Protein Data Bank (PDB) file [126] was inputted into the DiscoTope 2.0 (La Jolla Institute for Immunology, La Jolla, CA, USA; https://tools.iedb.org/discotope/ accessed on 25 March 2025) search field. DiscoTope 2.0 is a bioinformatics server based on the calculation of the number of contacts between amino acid residues and the probability that a residue is part of an epitope [69,127]. The default settings were selected and a threshold value of −3.7 was applied, which corresponds to a specificity of 75% and a sensitivity of 47% [69,127]. According to the updated version of the server, multiple epitopes for the same antigen were treated as a single entity [69]. The same methods were used to identify conformational B-cell epitopes of TPO (https://alphafold.ebi.ac.uk/entry/P07202, accessed on 25 March 2025) and Ro60 (https://alphafold.ebi.ac.uk/entry/P10155, accessed on 25 March 2025).

### 4.5. Statistical Analysis

Statistical analysis was performed using GraphPad Prism version 10.0.0 for Windows, GraphPad Software, Boston, MA, USA (https://www.graphpad.com/, accessed on 24 April 2025). In short, the data were first analyzed for parametric or non-parametric distribution using the Kolmogorov–Smirnov and the Shapiro–Wilk normality test. Given their normal distribution, the results of the analysis of predicted linear T-cell and B-cell epitopes of NIS were statistically compared with those of TPO and Ro60 using the two-tailed Student’s *t*-test for unpaired samples. Conversely, DiscoTope scores of NIS were statistically compared with those of TPO and Ro60 using the Mann–Whitney U test for unpaired samples due to their non-parametric distribution. Significance was set at *p*-values < 0.05 and a confidence interval of 95%.

## 5. Conclusions

According to the results of this computational study, NIS appears to contain linear and conformational epitopes for T and B cells that could explain the coexistence of SS and HT in genetically predisposed individuals. Although no homologies between the predicted epitopes of the NIS transporter and those of TPO and Ro60 were detected in this study, the T-cell epitopes of NIS could bind to the same HLA class II alleles that predispose to both autoimmune diseases.

We are confident that the results of this in silico study can pave the way for subsequent laboratory studies to further elucidate the role of NIS in the pathogenesis of SS in genetically predisposed patients with HT, including the assessment of the presence, titers and frequency of anti-NIS antibodies in patients with SS and their correlation with disease severity.

## Figures and Tables

**Table 1:** NIS T-cell epitopes that bind HLA class II alleles with a percentile rank ≤ 0.5 according to the TepiTool analysis. Abbreviations: HLA, human leukocyte antigen.

Peptide Start	Peptide End	Peptide Sequence	Percentile Rank	Allele
230	244	NLMDFNPDPRSRYTF	0.01	HLA-DRB1*03:05
230	244	NLMDFNPDPRSRYTF	0.02	HLA-DRB1*03:40
230	244	NLMDFNPDPRSRYTF	0.03	HLA-DRB1*03:14
380	394	PRKLVIISKGLSLIY	0.03	HLA-DRB1*11:13
380	394	PRKLVIISKGLSLIY	0.07	HLA-DRB1*08:31
380	394	PRKLVIISKGLSLIY	0.08	HLA-DRB1*08:04
380	394	PRKLVIISKGLSLIY	0.08	HLA-DRB1*14:15
380	394	PRKLVIISKGLSLIY	0.09	HLA-DRB1*11:25
380	394	PRKLVIISKGLSLIY	0.09	HLA-DRB1*11:27
380	394	PRKLVIISKGLSLIY	0.11	HLA-DRB1*11:08
380	394	PRKLVIISKGLSLIY	0.12	HLA-DRB1*11:04
380	394	PRKLVIISKGLSLIY	0.12	HLA-DRB1*11:06
380	394	PRKLVIISKGLSLIY	0.12	HLA-DRB1*11:52
380	394	PRKLVIISKGLSLIY	0.13	HLA-DRB1*11:19
380	394	PRKLVIISKGLSLIY	0.14	HLA-DRB1*11:01
380	394	PRKLVIISKGLSLIY	0.14	HLA-DRB1*11:05
380	394	PRKLVIISKGLSLIY	0.14	HLA-DRB1*11:09
380	394	PRKLVIISKGLSLIY	0.14	HLA-DRB1*11:10
380	394	PRKLVIISKGLSLIY	0.14	HLA-DRB1*11:15
380	394	PRKLVIISKGLSLIY	0.14	HLA-DRB1*11:29
380	394	PRKLVIISKGLSLIY	0.15	HLA-DRB1*08:02
380	394	PRKLVIISKGLSLIY	0.15	HLA-DRB1*08:09
380	394	PRKLVIISKGLSLIY	0.16	HLA-DRB1*11:37
368	382	EDLIKPRLRSLAPRK	0.17	HLA-DRB1*11:03
380	394	PRKLVIISKGLSLIY	0.39	HLA-DRB1*08:10
380	394	PRKLVIISKGLSLIY	0.43	HLA-DRB1*08:12
368	382	EDLIKPRLRSLAPRK	0.46	HLA-DRB1*11:11
368	382	EDLIKPRLRSLAPRK	0.48	HLA-DRB1*11:02
368	382	EDLIKPRLRSLAPRK	0.48	HLA-DRB1*11:16
380	394	PRKLVIISKGLSLIY	0.49	HLA-DRB1*08:06
380	394	PRKLVIISKGLSLIY	0.5	HLA-DRB1*11:03

**Table 2:** Linear B-cell epitopes of NIS according to the ABCpred analysis, sorted by the degree of affinity.

Rank	Sequence	Start Position	Score
1	AGSWTPCVGHDGGRDQ	622	0.94
1	LGRISAPDQYMPLLVL	314	0.94
2	MEAVETGERPTFGAWD	1	0.92
3	GVLQGSFTVMGVISGP	410	0.90
4	TGIICTFYTAVGGMKA	170	0.89
4	TALLFMPVFYRLGLTS	101	0.89
5	ALSVNASGLLDPALLP	484	0.88
5	MGVISGPLLGAFILGM	419	0.88
6	QTASVAPKEEVAILDD	569	0.87
6	LISCLTGPTKRSTLAP	544	0.87
7	FYTDCDPLLLGRISAP	305	0.86
8	DSSRAPSSGMDASRPA	502	0.85
9	GPTKRSTLAPGLLWWD	550	0.84
9	SFYAISYLYYGALGTL	521	0.84
9	GATLYPPSEQTMRVLP	461	0.84
9	GMFLPACNTPGVLAGL	433	0.84
9	PRSRYTFWTFVVGGTL	237	0.84
9	GLDIWASLLSTGIICT	160	0.84
10	FFLGQKELEGAGSWTP	612	0.83
10	GTLSTASTSINAMAAV	349	0.83
10	FGAWDYGVFALMLLVS	12	0.83
11	QRSAEDFFTGGRRLAA	41	0.82
11	EDLPGVPGLFLACAYS	333	0.82
11	ACCGIVMFVFYTDCDP	296	0.82
11	GGTLVWLSMYGVNQAQ	249	0.82
12	AVTVEDLIKPRLRSLA	363	0.81
13	GVPSEAYRYGLKFLWM	75	0.80
14	GIVIYAPALILNQVGL	146	0.79
15	GLLWWDLARQTASVAP	560	0.77
15	LTSTYEYLEMRFSRAV	114	0.77
16	ASRPALADSFYAISYL	513	0.76
17	PEELPTGNKKPPGFLP	590	0.75
18	PSEQTMRVLPSSAARC	467	0.74
18	YGVNQAQVQRYVACRT	258	0.74
18	YIVATMLYTGIVIYAP	137	0.74
19	PVFYRLGLTSTYEYLE	107	0.73
20	YRYGLKFLWMCLGQLL	81	0.72
21	PKEEVAILDDNLVKGP	575	0.71
21	INLMDFNPDPRSRYTF	228	0.71
22	GLALSLWVALGATLYP	451	0.70
22	LIVSSAACCGIVMFVF	290	0.70
22	ARGVMLVGGPRQVLTL	206	0.70
23	YGALGTLTTVLCGALI	530	0.69
23	ISKGLSLIYGSACLTV	385	0.69
23	PGLFLACAYSGTLSTA	339	0.69
23	AVGGMKAVVWTDVFQV	179	0.69
24	LGAFILGMFLPACNTP	427	0.67
24	QVLTLAQNHSRINLMD	217	0.67
25	IKPRLRSLAPRKLVII	370	0.65
25	IGLWVGLARGGQRSAE	30	0.65
26	SINAMAAVTVEDLIKP	357	0.64
26	CRTEKQAKLALLINQV	271	0.64
26	GFWVVLARGVMLVGGP	200	0.64
27	RVLPSSAARCVALSVN	473	0.63
28	VGLSLSASFMSAVQVL	59	0.61
28	GRRLAALPVGLSLSAS	51	0.61
29	PALILNQVGLDIWASL	152	0.60
30	TVAALSSLLGGGVLQG	399	0.59
30	LIYGSACLTVAALSSL	391	0.59
30	AVVWTDVFQVVVMLSG	185	0.59
31	FLPTNEDRLFFLGQKE	603	0.57
32	TPGVLAGLGAGLALSL	441	0.54
33	VQRYVACRTEKQAKLA	265	0.53
33	MRFSRAVRLCGTLQYI	123	0.53

**Table 3:** Conformational B-cell epitopes of NIS according to DiscoTope 2.0 analysis (positive predictions).

Chain ID	Residue ID	Residue Name	Contact Number	Propensity Score	DiscoTope Score
A	636	ARG	2	4.713	3.941
A	598	ASN	0	4.334	3.835
A	638	GLN	1	4.433	3.808
A	637	ASP	4	4.702	3.702
A	596	THR	0	4.057	3.591
A	597	GLY	3	4.148	3.326
A	599	LYS	1	3.801	3.249
A	601	PRO	2	3.921	3.24
A	602	PRO	0	3.499	3.096
A	600	LYS	3	3.836	3.05
A	635	GLY	1	3.494	2.977
A	639	GLN	2	3.608	2.963
A	595	PRO	1	2.901	2.452
A	603	GLY	1	2.866	2.421
A	634	GLY	3	2.53	1.894
A	604	PHE	4	2.659	1.893
A	640	GLU	1	2.049	1.698
A	641	THR	1	1.931	1.594
A	633	ASP	1	1.699	1.389
A	605	LEU	0	1.487	1.316
A	606	PRO	3	1.792	1.241
A	607	THR	4	1.727	1.068
A	594	LEU	5	1.61	0.85
A	593	GLU	1	0.885	0.668
A	632	HIS	0	0.494	0.438
A	609	GLU	0	0.139	0.123
A	608	ASN	3	0.505	0.102
A	592	GLU	2	0.191	−0.061
A	642	ASN	0	−0.121	−0.107
A	591	PRO	0	−0.205	−0.181
A	510	SER	2	−0.109	−0.327
A	511	GLY	5	−0.08	−0.645
A	631	GLY	3	−0.46	−0.752
A	507	ALA	6	−0.09	−0.77
A	610	ASP	1	−0.778	−0.804
A	590	GLY	0	−0.909	−0.805
A	621	GLU	0	−1.034	−0.915
A	508	PRO	6	−0.507	−1.139
A	643	LEU	3	−1.053	−1.277
A	509	SER	6	−0.865	−1.456
A	619	GLU	1	−1.7	−1.619
A	630	VAL	0	−1.947	−1.723
A	589	LYS	9	−0.814	−1.755
A	620	LEU	3	−1.671	−1.824
A	623	ALA	0	−2.175	−1.925
A	622	GLY	3	−1.807	−1.944
A	588	VAL	6	−1.821	−2.302
A	506	ARG	1	−2.586	−2.403
A	624	GLY	3	−2.414	−2.481
A	627	THR	1	−2.684	−2.49
A	625	SER	0	−2.921	−2.585
A	611	ARG	3	−2.599	−2.645
A	586	ASN	7	−2.083	−2.648
A	628	PRO	3	−2.61	−2.655
A	618	LYS	2	−2.844	−2.747
A	512	MET	9	−2.04	−2.84
A	629	CYS	1	−3.096	−2.855
A	587	LEU	6	−2.51	−2.912
A	514	ALA	0	−3.363	−2.977
A	626	TRP	2	−3.117	−2.988
A	576	PRO	4	−2.87	−3.0
A	585	ASP	6	−2.785	−3.155
A	578	GLU	1	−3.507	−3.218
A	577	LYS	1	−3.515	−3.226
A	582	ILE	5	−3.083	−3.303
A	579	GLU	6	−2.973	−3.321
A	505	SER	1	−3.768	−3.45
A	617	GLN	2	−3.679	−3.486

**Table 4:** Comparison of linear T-cell epitopes of NIS, TPO and Ro60 with the lowest percentile rank binding the same HLA allele. The HLA class II alleles are selected based on the highest affinity (lower percentile rank) of NIS epitopes. Abbreviations: HLA, human leukocyte antigen.

Allele	Percentile Rank for Predicted NIS T-Cell Epitopes	Percentile Rank for Predicted TPO T-Cell Epitopes	Percentile Rank for Predicted Ro60 T-Cell Epitopes
HLA-DRB1*03:05	0.01	1.4	0.26
HLA-DRB1*03:14	0.03	1.7	0.23
HLA-DRB1*03:40	0.02	1.4	0.33
HLA-DRB1*08:02	0.15	0.1	0.62
HLA-DRB1*08:04	0.08	0.34	0.42
HLA-DRB1*08:06	0.49	0.08	0.75
HLA-DRB1*08:09	0.15	0.1	0.62
HLA-DRB1*08:10	0.39	0.03	0.64
HLA-DRB1*08:12	0.43	0.03	0.84
HLA-DRB1*08:31	0.07	0.28	0.27
HLA-DRB1*11:01	0.14	0.07	0.56
HLA-DRB1*11:02	0.48	0.5	0.3
HLA-DRB1*11:03	0.17	0.2	1.3
HLA-DRB1*11:04	0.12	0.23	0.25
HLA-DRB1*11:05	0.14	0.15	0.6
HLA-DRB1*11:06	0.12	0.22	0.24
HLA-DRB1*11:08	0.11	0.16	0.64
HLA-DRB1*11:09	0.14	0.07	0.56
HLA-DRB1*11:10	0.14	0.07	0.56
HLA-DRB1*11:11	0.46	0.43	1.1
HLA-DRB1*11:13	0.03	0.46	0.25
HLA-DRB1*11:15	0.14	0.07	0.56
HLA-DRB1*11:16	0.48	0.5	0.3
HLA-DRB1*11:19	0.13	0.18	0.65
HLA-DRB1*11:25	0.09	0.17	0.35
HLA-DRB1*11:27	0.09	0.19	0.5
HLA-DRB1*11:29	0.14	0.07	0.56
HLA-DRB1*11:37	0.16	0.08	0.54
HLA-DRB1*11:52	0.12	0.27	0.62
HLA-DRB1*14:15	0.08	0.34	0.42

**Table 5:** List of HLA class II alleles that predispose to SS in the world population according to literature data. Abbreviations: HLA, human leukocyte antigen; NA, not available.

HLA Class II Allele	Population	Allele Frequency	References
HLA-DRB1*01:01	Mexican	0.003–0.13	[12]
HLA-DR3	Australian	0.12–0.14	[88]
HLA-DR3	French	0.09–0.13	[89]
HLA-DR3	Spanish	0.06–0.20	[50]
HLA-DR3	Caucasian (mostly Norwegian)	0.06–0.14	[90]
HLA-DR3	American	0.05–0.13	[12]
HLA-DRB1*03:01	French	0.08–0.15	[12,91,92]
HLA-DRB1*03:01	Swiss	NA	[91]
HLA-DRB1*03:01	American	0.01–0.15	[91]
HLA-DRB1*03:01	Norwegian	0.14	[50]
HLA-DRB1*03:01	Californian Caucasian	0.06	[93]
HLA-DRB1*03:01	Columbian	0.02–0.15	[12,94]
HLA-DRB1*03:01	Latin American	0–0.15	[95]
HLA-DRB1*03:01	Japanese	0.0007–0.008	[12]
HLA-DRB1*03:01	Greek	0.07–0.09	[12]
HLA-DRB1*03:01	Italian	0.06–0.56	[12]
HLA-DRB1*03:01	Finnish	0.06–0.10	[12]
HLA-DRB1*03:01	Norwegian	0.14	[12]
HLA-DRB1*03:01	British	0.10–0.17	[12]
HLA-DRB1*03:01	Australian	0.05	[12]
HLA-DRB1*04:05	Japanese	0.08–0.16	[12,50,93]
HLA-DR5	Greek	NA	[96]
HLA-DR8	Taiwanese	0.04–0.72	[13]
HLA-DRB1*08:03	Chinese	0.004–0.09	[12,50,93]
HLA-DRB1*08:032	Japanese	NA	[12,97]
HLA-DR11	Spanish	0–0.24	[12,50]
HLA-DRB1*11:01	Israeli Jews	0.01–0.11	[12]
HLA-DRB1*11:01	Greek of non-Jewish origin	0.08–0.11	[12]
HLA-DRB1*11:04	Israeli Jews	0.0001–0.34	[12]
HLA-DRB1*11:04	Greek of non-Jewish origin	0.10–0.19	[12]
HLA-DR15	Australian	0.14–0.16	[88]
HLA-DR15	French	0.10–0.18	[89,92]
HLA-DR15	Danish	NA	[12]
HLA-DRB1*15:01	French	0.08–0.13	[12]
HLA-DRB3*01:01	Norwegian	NA	[50]
HLA-DRB3*01:01	Californian Caucasian	NA	[93]
HLA-DRB3*01:01	Danish	NA	[98]
HLA-DRB3*01:01	Japanese	NA	[12]
HLA-DRB4*01:01	Japanese	NA	[12,93]
HLA-DRw52	American Caucasian	NA	[12]
HLA-DRw52	British	NA	[12]
HLA-DRw53	Japanese	NA	[12]
HLA-DQA1*01	Israeli	NA	[12]
HLA-DQA1*01	Israeli Jew	NA	[12]
HLA-DQA1*01	Greek	0.40	[12]
HLA-DQA1*01:01	European	0.01–0.27	[12]
HLA-DQA1*01:01	Afroamerican	0.02–0.17	[12]
HLA-DQA1*01:02	Australian	0.10–0.15	[88]
HLA-DQA1*01:03	Chinese	0.06–0.15	[12,93]
HLA-DQA1*01:03	Japanese	0.15–0.23	[97]
HLA-DQA1*03:01	Japanese	0.11–0.42	[12,93]
HLA-DQA1*03:01	Danish	NA	[12]
HLA-DQA1*05:01	French	0.22–0.32	[91]
HLA-DQA1*05:01	Swiss	NA	[91]
HLA-DQA1*05:01	American	0.15–0.94	[91]
HLA-DQA1*05:01	Australian	0.13–0.31	[12,88]
HLA-DQA1*05:01	Danish	0.15	[12,50]
HLA-DQA1*05:01	Israeli	0.26–0.29	[50]
HLA-DQA1*05:01	Jew	0.26–0.29	[50]
HLA-DQA1*05:01	Greek	0.07–0.43	[12,50]
HLA-DQA1*05:01	Caucasian (mostly Norwegian)	0.22	[90]
HLA-DQA1*05:01	American Caucasian	0.23–0.26	[99]
HLA-DQA1*05:01	Black American	0.15–0.94	[99]
HLA-DQA1*05:01	Japanese	0.001–0.09	[12]
HLA-DQA1*05:01	Finnish	NA	[12]
HLA-DQA1*05:01	Norwegian	0.22	[12]
HLA-DQA1*05:01	Italian	0.07–0.41	[100]
HLA-DQB1*02	Australian	NA	[88]
HLA-DQB1*02	French	0.15–0.27	[12,89]
HLA-DQB1*02	Norwegian	0.06–0.19	[12,90]
HLA-DQB1*02:01	French	0.08–0.25	[12,91]
HLA-DQB1*02:01	Swiss	0.06–0.13	[91]
HLA-DQB1*02:01	American Caucasian	0.08–0.15	[91,93,99]
HLA-DQB1*02:01	Afroamerican	0.07–0.22	[99]
HLA-DQB1*02:01	Danish	0.21	[98]
HLA-DQB1*02:01	Columbian	0.02–0.33	[12,94]
HLA-DQB1*02:01	Japanese	0.002–0.01	[12]
HLA-DQB1*02:01	European	0.05–0.59	[12]
HLA-DQB1*02:01	Afroamerican	0.07–0.22	[12]
HLA-DQB1*02:01	Italian	0.07–0.59	[100]
HLA-DQB1*03	Japanese	NA	[97]
HLA-DQB1*03:01	French	0.16–0.31	[91]
HLA-DQB1*03:01	Swiss	0.17–0.27	[91]
HLA-DQB1*03:01	American	0.16–0.94	[91]
HLA-DQB1*03:01	Israeli	0.19–0.30	[50]
HLA-DQB1*03:01	Jew	0.19–0.30	[50]
HLA-DQB1*03:01	Greek	0.27–0.34	[50]
HLA-DQB1*04:01	Japanese	0.09–0.16	[12,50,93]
HLA-DQB1*06	Japanese	NA	[97]
HLA-DQB1*06:01	Chinese	0.04–0.26	[12,50]
HLA-DQB1*06:01	Japanese	0.16–0.22	[12,97]
HLA-DQB1*06:02	Australian	0.04	[88]
HLA-DQB1*06:02	Danish	0.17	[50]
HLA-DQB1*06:02	French	0.08–0.16	[12]

**Table 6:** List of HLA class II alleles that predispose to HT in the world population, according to literature data. Abbreviations: HLA, human leukocyte antigen; NA, not available.

HLA Class II Allele	Population	Allele Frequency	References
HLA-DR3	Romanian	0.11	[14]
HLA-DR3	Hungarian	NA	[101]
HLA-DR3	British	0.13–0.16	[102,103]
HLA-DR3	Spanish	0.06–0.20	[51]
HLA-DRB1*03:01	Iranian	0.05–0.29	[104]
HLA-DRB1*03:01	Mexican	0.003–0.14	[105]
HLA-DR4	British	0.13–0.20	[102]
HLA-DR4	Greek	0.04–0.10	[106]
HLA-DR4	Korean	0.20	[107]
HLA-DR4	Italian	0.03–0.11	[108]
HLA-DRB1*04:02	Iranian	0.02–0.09	[104]
HLA-DRB1*04:03	Japanese	0.01–0.06	[109]
HLA-DRB1*04:04	Mexican	0.01–0.21	[105]
HLA-DRB1*04:05	Greek	0.01–0.03	[106]
HLA-DRB1*04:05	Japanese	0.08–0.16	[110]
HLA-DRB1*04:05	Iranian	0.01–0.02	[104]
HLA-DR5	Southern Indian	NA	[111]
HLA-DR5	Canadian	NA	[112]
HLA-DR5	Danish	NA	[113]
HLA-DR7	Japanese	0.006–0.008	[109]
HLA-DR8	British	0.02–0.04	[102]
HLA-DR8	Korean	0.10	[107]
HLA-DRB1*08:02	Japanese	0.01–0.10	[110]
HLA-DRB1*08:032	Japanese	NA	[109,114]
HLA-DR9	Southern Chinese	0.06–0.19	[115]
HLA-DRB1*09:01	Japanese	0.12–0.16	[109,110,114,116]
HLA-DR11	Southern Indian	0.03–0.18	[111]
HLA-DRB1*11:04	Iranian	NA	[104]
HLA-DR12	Southern Indian	0.02–0.07	[111]
HLA-DR13	British	0.09–0.11	[102]
HLA-DRB1*13:01	Iranian	0.03–0.15	[104]
HLA-DRB1*14:04	Northern Indian	0.003–0.13	[117]
HLA-DR16	Romanian	0.10–0.11	[14]
HLA-DRB4*01:01	Japanese	NA	[109]
HLA-DRw3	Canadian	NA	[118]
HLA-DQA1*01	British	0.34	[102]
HLA-DQA1*03:01	Greek	0.05–0.08	[106]
HLA-DQA1*03:01	Lebanese	NA	[15]
HLA-DQA1*03:01	Southern Indian	0.09–0.11	[111]
HLA-DQA1*03:02	Japanese	0.14	[110]
HLA-DQA1*03:03	Japanese	0.17	[110]
HLA-DQA1*04:01	Japanese	0.01–0.08	[110]
HLA-DQA1*05:01	Lebanese	NA	[15]
HLA-DQB1*02:01	Greek	0.08–0.16	[106]
HLA-DQB1*02:01	Southern Indian	0.05–0.23	[111]
HLA-DQB1*03	Brazilian	0.29–0.73	[119]
HLA-DQB1*03:01	British	0.13–0.22	[102]
HLA-DQB1*03:01	Italian	0.03–0.35	[108]
HLA-DQB1*03:02	Greek	0.03–0.09	[106]
HLA-DQB1*03:02	Lebanese	0.05–0.15	[15]
HLA-DQB1*03:03	Japanese	0.11–0.18	[110,116]
HLA-DQB1*04:01	Japanese	0.09–0.16	[110]
HLA-DQB1*04:02	British	0.003–0.02	[102]
HLA-DQB1*04:02	Japanese	0.03–0.08	[110]
HLA-DQB1*05	Northern Greek	NA	[120]
HLA-DQB1*06	British	0.21–0.27	[102]
HLA-DQB1*06:01	Japanese	0.16–0.22	[121]

**Table 7:** Panel of HLA class II alleles predisposing to SS and HT used in the computational analysis. Abbreviations: HLA, human leukocyte antigen.

Host Species	Human
Class allele	Class II
1	HLA-DRB1*03:01
2	HLA-DRB1*03:05
3	HLA-DRB1*03:06
4	HLA-DRB1*03:07
5	HLA-DRB1*03:14
6	HLA-DRB1*03:15
7	HLA-DRB1*03:23
8	HLA-DRB1*03:36
9	HLA-DRB1*03:40
10	HLA-DRB1*04:05
11	HLA-DRB1*08:01
12	HLA-DRB1*08:02
13	HLA-DRB1*08:03
14	HLA-DRB1*08:04
15	HLA-DRB1*08:05
16	HLA-DRB1*08:06
17	HLA-DRB1*08:07
18	HLA-DRB1*08:09
19	HLA-DRB1*08:10
20	HLA-DRB1*08:11
21	HLA-DRB1*08:12
22	HLA-DRB1*08:14
23	HLA-DRB1*08:16
24	HLA-DRB1*08:17
25	HLA-DRB1*08:31
26	HLA-DRB1*11:01
27	HLA-DRB1*11:02
28	HLA-DRB1*11:03
29	HLA-DRB1*11:04
30	HLA-DRB1*11:05
31	HLA-DRB1*11:06
32	HLA-DRB1*11:07
33	HLA-DRB1*11:08
34	HLA-DRB1*11:09
35	HLA-DRB1*11:10
36	HLA-DRB1*11:11
37	HLA-DRB1*11:13
38	HLA-DRB1*11:14
39	HLA-DRB1*11:15
40	HLA-DRB1*11:16
41	HLA-DRB1*11:19
42	HLA-DRB1*11:20
43	HLA-DRB1*11:21
44	HLA-DRB1*11:25
45	HLA-DRB1*11:27
46	HLA-DRB1*11:29
47	HLA-DRB1*11:37
48	HLA-DRB1*11:52
49	HLA-DRB1*14:15
50	HLA-DRB4*01:01

## Data Availability

The original contributions presented in this study are included in the article/Supplementary Materials. Further inquiries can be directed to the corresponding author.

## Supplementary Materials

The following supporting information can be downloaded at: https://www.mdpi.com/article/10.3390/ijms27010200/s1.

## Abbreviations

The following abbreviations are used in this manuscript:

APC	Antigen-presenting cell
BAFF	B-cell activating factor
CCP	Complement Control Protein
CD	Cluster of differentiation
cDC	Classical dendritic cell
ELISA	Enzyme-linked immunosorbent assay
HLA	Human leukocyte antigen
HT	Hashimoto’s Thyroiditis
IDR	Immune Domain Region
IEDB	Immune Epitope Database
IFN	Interferon
IL	Interleukin
M-CSF	Macrophage colony-stimulating factor
MPO	Myeloperoxidase
NA	Not available
NIS	Sodium-iodide symporter
PDB	Protein Data Bank
pDC	Plasmacytoid dendritic cell
RA	Rheumatoid arthritis
Ro60	Ro60 Y RNA-binding protein
TCR	T-cell receptor
Tfh	T helper follicular cell
TG	Thyroglobulin
TGF	Transforming growth factor
TNF	Tumor necrosis factor
Th	T helper cell
TPO	Thyroid peroxidase
TSH-R	Thyroid-stimulating hormone receptor
Treg	T regulatory cell
TROVE	TAR RNA-binding protein and oxidative stress response-related
SLE	Systemic lupus erythematosus
SD	Standard deviation
SS	Sjögren’s syndrome
VWFA	Von Willebrand Factor A
