# Supplementary material for: Predicted T-Cell and B-Cell Epitopes of NIS: Where Do Sjögren’s Syndrome and Hashimoto’s Thyroiditis Converge?
Source: Int J Mol Sci. 2025 Dec 24;27(1):200. doi: 10.3390/ijms27010200 (PMC12785876; doi:10.3390/ijms27010200)
Supplement: Supplementary file 1 [file ijms-27-00200-s001.zip › Table S1 IJMS REV.pdf]

Peptide Start	*Peptide End*	*Peptide Sequence*	*Percentile rank*	*Allele*
**230**	244	NLMDFNPDPRSRYTF	0.01	HLA-DRB1*03:05
**230**	244	NLMDFNPDPRSRYTF	0.02	HLA-DRB1*03:40
**230**	244	NLMDFNPDPRSRYTF	0.03	HLA-DRB1*03:14
**380**	394	PRKLVIISKGLSLIY	0.03	HLA-DRB1*11:13
**380**	394	PRKLVIISKGLSLIY	0.07	HLA-DRB1*08:31
**380**	394	PRKLVIISKGLSLIY	0.08	HLA-DRB1*08:04
**380**	394	PRKLVIISKGLSLIY	0.08	HLA-DRB1*14:15
**380**	394	PRKLVIISKGLSLIY	0.09	HLA-DRB1*11:25
**380**	394	PRKLVIISKGLSLIY	0.09	HLA-DRB1*11:27
**380**	394	PRKLVIISKGLSLIY	0.11	HLA-DRB1*11:08
**380**	394	PRKLVIISKGLSLIY	0.12	HLA-DRB1*11:04
**380**	394	PRKLVIISKGLSLIY	0.12	HLA-DRB1*11:06
**380**	394	PRKLVIISKGLSLIY	0.12	HLA-DRB1*11:52
**380**	394	PRKLVIISKGLSLIY	0.13	HLA-DRB1*11:19
**380**	394	PRKLVIISKGLSLIY	0.14	HLA-DRB1*11:01
**380**	394	PRKLVIISKGLSLIY	0.14	HLA-DRB1*11:05
**380**	394	PRKLVIISKGLSLIY	0.14	HLA-DRB1*11:09
**380**	394	PRKLVIISKGLSLIY	0.14	HLA-DRB1*11:10
**380**	394	PRKLVIISKGLSLIY	0.14	HLA-DRB1*11:15
**380**	394	PRKLVIISKGLSLIY	0.14	HLA-DRB1*11:29
**380**	394	PRKLVIISKGLSLIY	0.15	HLA-DRB1*08:02
**380**	394	PRKLVIISKGLSLIY	0.15	HLA-DRB1*08:09
**380**	394	PRKLVIISKGLSLIY	0.16	HLA-DRB1*11:37
**368**	382	EDLIKPRLRSLAPRK	0.17	HLA-DRB1*11:03
**380**	394	PRKLVIISKGLSLIY	0.39	HLA-DRB1*08:10
**380**	394	PRKLVIISKGLSLIY	0.43	HLA-DRB1*08:12
**368**	382	EDLIKPRLRSLAPRK	0.46	HLA-DRB1*11:11
**368**	382	EDLIKPRLRSLAPRK	0.48	HLA-DRB1*11:02
**368**	382	EDLIKPRLRSLAPRK	0.48	HLA-DRB1*11:16
**380**	394	PRKLVIISKGLSLIY	0.49	HLA-DRB1*08:06

**380**	394	PRKLVIISKGLSLIY	0.5	HLA-DRB1*11:03
**230**	244	NLMDFNPDPRSRYTF	0.52	HLA-DRB1*03:15
**368**	382	EDLIKPRLRSLAPRK	0.52	HLA-DRB1*11:21
**265**	279	QVQRYVACRTEKQAK	0.56	HLA-DRB1*11:37
**230**	244	NLMDFNPDPRSRYTF	0.61	HLA-DRB1*03:01
**230**	244	NLMDFNPDPRSRYTF	0.61	HLA-DRB1*03:23
**230**	244	NLMDFNPDPRSRYTF	0.61	HLA-DRB1*03:36
**230**	244	NLMDFNPDPRSRYTF	0.62	HLA-DRB1*03:07
**230**	244	NLMDFNPDPRSRYTF	0.62	HLA-DRB1*11:07
**265**	279	QVQRYVACRTEKQAK	0.68	HLA-DRB1*11:05
**380**	394	PRKLVIISKGLSLIY	0.69	HLA-DRB1*11:11
**230**	244	NLMDFNPDPRSRYTF	0.7	HLA-DRB1*03:06
**380**	394	PRKLVIISKGLSLIY	0.72	HLA-DRB1*08:11
**265**	279	QVQRYVACRTEKQAK	0.79	HLA-DRB1*08:02
**265**	279	QVQRYVACRTEKQAK	0.79	HLA-DRB1*08:09
**265**	279	QVQRYVACRTEKQAK	0.84	HLA-DRB1*11:01
**265**	279	QVQRYVACRTEKQAK	0.84	HLA-DRB1*11:09
**265**	279	QVQRYVACRTEKQAK	0.84	HLA-DRB1*11:10
**265**	279	QVQRYVACRTEKQAK	0.84	HLA-DRB1*11:15
**265**	279	QVQRYVACRTEKQAK	0.84	HLA-DRB1*11:29
**380**	394	PRKLVIISKGLSLIY	0.85	HLA-DRB1*11:02
**380**	394	PRKLVIISKGLSLIY	0.85	HLA-DRB1*11:16
**44**	58	AEDFFTGGRRLAALP	0.91	HLA-DRB1*11:27
**310**	324	CDPLLLGRISAPDQY	0.96	HLA-DRB1*08:06
**265**	279	QVQRYVACRTEKQAK	0.96	HLA-DRB1*11:08
**368**	382	EDLIKPRLRSLAPRK	0.97	HLA-DRB1*11:25
**265**	279	QVQRYVACRTEKQAK	0.98	HLA-DRB1*11:19
**265**	279	QVQRYVACRTEKQAK	1.1	HLA-DRB1*04:05
**44**	58	AEDFFTGGRRLAALP	1.1	HLA-DRB1*11:01
**44**	58	AEDFFTGGRRLAALP	1.1	HLA-DRB1*11:09
**44**	58	AEDFFTGGRRLAALP	1.1	HLA-DRB1*11:10
**44**	58	AEDFFTGGRRLAALP	1.1	HLA-DRB1*11:15

**380**	394	PRKLVIISKGLSLIY	1.1	HLA-DRB1*11:21
**265**	279	QVQRYVACRTEKQAK	1.1	HLA-DRB1*11:27
**44**	58	AEDFFTGGRRLAALP	1.1	HLA-DRB1*11:29
**610**	624	DRLFFLGQKELEGAG	1.2	HLA-DRB1*08:05
**44**	58	AEDFFTGGRRLAALP	1.2	HLA-DRB1*11:11
**380**	394	PRKLVIISKGLSLIY	1.3	HLA-DRB1*08:07
**380**	394	PRKLVIISKGLSLIY	1.3	HLA-DRB1*11:14
**380**	394	PRKLVIISKGLSLIY	1.3	HLA-DRB1*11:20
**44**	58	AEDFFTGGRRLAALP	1.3	HLA-DRB1*11:37
**310**	324	CDPLLLGRISAPDQY	1.4	HLA-DRB1*08:01
**380**	394	PRKLVIISKGLSLIY	1.4	HLA-DRB1*08:05
**265**	279	QVQRYVACRTEKQAK	1.4	HLA-DRB1*08:05
**310**	324	CDPLLLGRISAPDQY	1.4	HLA-DRB1*08:10
**310**	324	CDPLLLGRISAPDQY	1.4	HLA-DRB1*08:16
**380**	394	PRKLVIISKGLSLIY	1.5	HLA-DRB1*08:01
**380**	394	PRKLVIISKGLSLIY	1.5	HLA-DRB1*08:16
**578**	592	EEVAILDDNLVKGPE	1.6	HLA-DRB1*03:06
**610**	624	DRLFFLGQKELEGAG	1.6	HLA-DRB1*08:01
**610**	624	DRLFFLGQKELEGAG	1.6	HLA-DRB1*08:16
**310**	324	CDPLLLGRISAPDQY	1.7	HLA-DRB1*08:12
**368**	382	EDLIKPRLRSLAPRK	1.7	HLA-DRB1*11:14
**368**	382	EDLIKPRLRSLAPRK	1.7	HLA-DRB1*11:20
**578**	592	EEVAILDDNLVKGPE	1.8	HLA-DRB1*03:01
**578**	592	EEVAILDDNLVKGPE	1.8	HLA-DRB1*03:23
**578**	592	EEVAILDDNLVKGPE	1.8	HLA-DRB1*03:36
**44**	58	AEDFFTGGRRLAALP	1.8	HLA-DRB1*08:02
**380**	394	PRKLVIISKGLSLIY	1.8	HLA-DRB1*08:03
**44**	58	AEDFFTGGRRLAALP	1.8	HLA-DRB1*08:09
**380**	394	PRKLVIISKGLSLIY	1.8	HLA-DRB1*08:14
**51**	65	GRRLAALPVGLSLSA	1.8	HLA-DRB1*08:31
**368**	382	EDLIKPRLRSLAPRK	1.9	HLA-DRB1*11:04
**44**	58	AEDFFTGGRRLAALP	1.9	HLA-DRB1*11:08

**265**	279	QVQRYVACRTEKQAK	2.1	HLA-DRB1*08:01
**265**	279	QVQRYVACRTEKQAK	2.1	HLA-DRB1*08:16
**44**	58	AEDFFTGGRRLAALP	2.1	HLA-DRB1*11:05
**368**	382	EDLIKPRLRSLAPRK	2.1	HLA-DRB1*11:06
**44**	58	AEDFFTGGRRLAALP	2.1	HLA-DRB1*11:19
**578**	592	EEVAILDDNLVKGPE	2.2	HLA-DRB1*03:15
**265**	279	QVQRYVACRTEKQAK	2.2	HLA-DRB1*08:03
**310**	324	CDPLLLGRISAPDQY	2.2	HLA-DRB1*08:03
**265**	279	QVQRYVACRTEKQAK	2.2	HLA-DRB1*08:14
**310**	324	CDPLLLGRISAPDQY	2.2	HLA-DRB1*08:14
**265**	279	QVQRYVACRTEKQAK	2.4	HLA-DRB1*08:11
**216**	230	PRQVLTLAQNHSRIN	2.4	HLA-DRB1*08:31
**578**	592	EEVAILDDNLVKGPE	2.5	HLA-DRB1*03:07
**265**	279	QVQRYVACRTEKQAK	2.5	HLA-DRB1*08:07
**578**	592	EEVAILDDNLVKGPE	2.5	HLA-DRB1*11:07
**216**	230	PRQVLTLAQNHSRIN	2.5	HLA-DRB4*01:01
**216**	230	PRQVLTLAQNHSRIN	2.7	HLA-DRB1*11:21
**610**	624	DRLFFLGQKELEGAG	2.8	HLA-DRB1*08:03
**610**	624	DRLFFLGQKELEGAG	2.8	HLA-DRB1*08:14
**363**	377	AAVTVEDLIKPRLRS	2.8	HLA-DRB1*11:13
**51**	65	GRRLAALPVGLSLSA	2.9	HLA-DRB1*08:04
**51**	65	GRRLAALPVGLSLSA	2.9	HLA-DRB1*14:15
**578**	592	EEVAILDDNLVKGPE	2.9	HLA-DRB4*01:01
**355**	369	ASTSINAMAAVTVED	3.0	HLA-DRB1*08:07
**27**	41	STGIGLWVGLARGGQ	3.1	HLA-DRB1*11:21
**610**	624	DRLFFLGQKELEGAG	3.1	HLA-DRB1*11:37
**27**	41	STGIGLWVGLARGGQ	3.2	HLA-DRB1*11:02
**27**	41	STGIGLWVGLARGGQ	3.2	HLA-DRB1*11:16
**578**	592	EEVAILDDNLVKGPE	3.3	HLA-DRB1*03:05
**310**	324	CDPLLLGRISAPDQY	3.3	HLA-DRB1*08:11
**216**	230	PRQVLTLAQNHSRIN	3.3	HLA-DRB1*11:02
**216**	230	PRQVLTLAQNHSRIN	3.3	HLA-DRB1*11:16

**578**	592	EEVAILDDNLVKGPE	3.5	HLA-DRB1*03:14
**610**	624	DRLFFLGQKELEGAG	3.6	HLA-DRB1*08:11
**468**	482	PSEQTMRVLPSSAAR	3.6	HLA-DRB1*08:31
**216**	230	PRQVLTLAQNHSRIN	3.6	HLA-DRB1*11:06
**216**	230	PRQVLTLAQNHSRIN	3.7	HLA-DRB1*11:04
**51**	65	GRRLAALPVGLSLSA	3.7	HLA-DRB1*11:06
**44**	58	AEDFFTGGRRLAALP	3.7	HLA-DRB1*11:14
**44**	58	AEDFFTGGRRLAALP	3.7	HLA-DRB1*11:20
**51**	65	GRRLAALPVGLSLSA	3.9	HLA-DRB1*11:04
**265**	279	QVQRYVACRTEKQAK	3.9	HLA-DRB1*11:11
**368**	382	EDLIKPRLRSLAPRK	3.9	HLA-DRB1*11:13
**230**	244	NLMDFNPDPRSRYTF	3.9	HLA-DRB1*11:14
**230**	244	NLMDFNPDPRSRYTF	3.9	HLA-DRB1*11:20
**209**	223	GVMLVGGPRQVLTLA	3.9	HLA-DRB1*11:52
**368**	382	EDLIKPRLRSLAPRK	4.1	HLA-DRB1*11:01
**610**	624	DRLFFLGQKELEGAG	4.1	HLA-DRB1*11:05
**368**	382	EDLIKPRLRSLAPRK	4.1	HLA-DRB1*11:09
**368**	382	EDLIKPRLRSLAPRK	4.1	HLA-DRB1*11:10
**368**	382	EDLIKPRLRSLAPRK	4.1	HLA-DRB1*11:15
**368**	382	EDLIKPRLRSLAPRK	4.1	HLA-DRB1*11:29
**578**	592	EEVAILDDNLVKGPE	4.2	HLA-DRB1*04:05
**150**	164	YAPALILNQVTGLDI	4.2	HLA-DRB1*04:05
**610**	624	DRLFFLGQKELEGAG	4.2	HLA-DRB1*11:01
**230**	244	NLMDFNPDPRSRYTF	4.2	HLA-DRB1*11:08
**610**	624	DRLFFLGQKELEGAG	4.2	HLA-DRB1*11:09
**610**	624	DRLFFLGQKELEGAG	4.2	HLA-DRB1*11:10
**610**	624	DRLFFLGQKELEGAG	4.2	HLA-DRB1*11:15
**610**	624	DRLFFLGQKELEGAG	4.2	HLA-DRB1*11:29
**468**	482	PSEQTMRVLPSSAAR	4.3	HLA-DRB1*04:05
**145**	159	TGIVIYAPALILNQV	4.3	HLA-DRB1*11:13
**380**	394	PRKLVIISKGLSLIY	4.4	HLA-DRB1*03:15
**216**	230	PRQVLTLAQNHSRIN	4.4	HLA-DRB1*08:04

**610**	624	DRLFFLGQKELEGAG	4.4	HLA-DRB1*11:27
**216**	230	PRQVLTLAQNHSRIN	4.4	HLA-DRB1*14:15
**225**	239	NHSRINLMDFNPDPR	4.5	HLA-DRB4*01:01
**578**	592	EEVAILDDNLVKGPE	4.6	HLA-DRB1*03:40
**368**	382	EDLIKPRLRSLAPRK	4.6	HLA-DRB1*08:31
**120**	134	YLEMRFSRAVRLCGT	4.6	HLA-DRB1*11:01
**120**	134	YLEMRFSRAVRLCGT	4.6	HLA-DRB1*11:09
**120**	134	YLEMRFSRAVRLCGT	4.6	HLA-DRB1*11:10
**120**	134	YLEMRFSRAVRLCGT	4.6	HLA-DRB1*11:15
**120**	134	YLEMRFSRAVRLCGT	4.6	HLA-DRB1*11:29
**380**	394	PRKLVIISKGLSLIY	4.7	HLA-DRB1*03:07
**380**	394	PRKLVIISKGLSLIY	4.7	HLA-DRB1*11:07
**51**	65	GRRLAALPVGLSLSA	4.7	HLA-DRB4*01:01
**230**	244	NLMDFNPDPRSRYTF	4.8	HLA-DRB1*11:19
**368**	382	EDLIKPRLRSLAPRK	4.8	HLA-DRB1*11:27
**51**	65	GRRLAALPVGLSLSA	4.9	HLA-DRB1*08:02
**51**	65	GRRLAALPVGLSLSA	4.9	HLA-DRB1*08:09
**610**	624	DRLFFLGQKELEGAG	4.9	HLA-DRB1*11:08
**216**	230	PRQVLTLAQNHSRIN	5.0	HLA-DRB1*11:13
**216**	230	PRQVLTLAQNHSRIN	5.0	HLA-DRB1*11:14
**216**	230	PRQVLTLAQNHSRIN	5.0	HLA-DRB1*11:20
**120**	134	YLEMRFSRAVRLCGT	5.0	HLA-DRB1*11:27
**368**	382	EDLIKPRLRSLAPRK	5.1	HLA-DRB1*08:04
**468**	482	PSEQTMRVLPSSAAR	5.1	HLA-DRB1*11:06
**368**	382	EDLIKPRLRSLAPRK	5.1	HLA-DRB1*14:15
**216**	230	PRQVLTLAQNHSRIN	5.2	HLA-DRB1*08:12
**216**	230	PRQVLTLAQNHSRIN	5.2	HLA-DRB1*11:03
**355**	369	ASTSINAMAAVTVED	5.2	HLA-DRB1*11:13
**610**	624	DRLFFLGQKELEGAG	5.3	HLA-DRB1*03:14
**216**	230	PRQVLTLAQNHSRIN	5.4	HLA-DRB1*03:07
**265**	279	QVQRYVACRTEKQAK	5.4	HLA-DRB1*03:14
**216**	230	PRQVLTLAQNHSRIN	5.4	HLA-DRB1*11:07

**368**	382	EDLIKPRLRSLAPRK	5.4	HLA-DRB1*11:19
**610**	624	DRLFFLGQKELEGAG	5.4	HLA-DRB1*11:19
**468**	482	PSEQTMRVLPSSAAR	5.6	HLA-DRB1*08:04
**368**	382	EDLIKPRLRSLAPRK	5.6	HLA-DRB1*11:05
**468**	482	PSEQTMRVLPSSAAR	5.6	HLA-DRB1*14:15
**610**	624	DRLFFLGQKELEGAG	5.7	HLA-DRB1*08:02
**610**	624	DRLFFLGQKELEGAG	5.7	HLA-DRB1*08:09
**51**	65	GRRLAALPVGLSLSA	5.7	HLA-DRB1*11:05
**216**	230	PRQVLTLAQNHSRIN	5.7	HLA-DRB1*11:25
**310**	324	CDPLLLGRISAPDQY	5.8	HLA-DRB1*08:05
**368**	382	EDLIKPRLRSLAPRK	5.8	HLA-DRB1*11:08
**363**	377	AAVTVEDLIKPRLRS	5.9	HLA-DRB1*08:10
**363**	377	AAVTVEDLIKPRLRS	5.9	HLA-DRB1*11:04
**363**	377	AAVTVEDLIKPRLRS	5.9	HLA-DRB1*11:06
**44**	58	AEDFFTGGRRLAALP	6.0	HLA-DRB1*08:01
**44**	58	AEDFFTGGRRLAALP	6.0	HLA-DRB1*08:05
**44**	58	AEDFFTGGRRLAALP	6.0	HLA-DRB1*08:16
**363**	377	AAVTVEDLIKPRLRS	6.1	HLA-DRB1*08:06
**225**	239	NHSRINLMDFNPDPR	6.2	HLA-DRB1*08:10
**27**	41	STGIGLWVGLARGGQ	6.2	HLA-DRB1*11:01
**27**	41	STGIGLWVGLARGGQ	6.2	HLA-DRB1*11:09
**27**	41	STGIGLWVGLARGGQ	6.2	HLA-DRB1*11:10
**27**	41	STGIGLWVGLARGGQ	6.2	HLA-DRB1*11:15
**27**	41	STGIGLWVGLARGGQ	6.2	HLA-DRB1*11:29
**44**	58	AEDFFTGGRRLAALP	6.4	HLA-DRB1*08:11
**51**	65	GRRLAALPVGLSLSA	6.4	HLA-DRB1*11:25
**265**	279	QVQRYVACRTEKQAK	6.5	HLA-DRB1*03:40
**355**	369	ASTSINAMAAVTVED	6.5	HLA-DRB1*08:12
**363**	377	AAVTVEDLIKPRLRS	6.5	HLA-DRB1*11:25
**610**	624	DRLFFLGQKELEGAG	6.6	HLA-DRB1*03:40
**363**	377	AAVTVEDLIKPRLRS	6.6	HLA-DRB1*08:12
**265**	279	QVQRYVACRTEKQAK	6.6	HLA-DRB1*08:31

**560**	574	PGLLWWDLARQTASV	6.6	HLA-DRB1*11:27
**363**	377	AAVTVEDLIKPRLRS	6.7	HLA-DRB1*08:31
**560**	574	PGLLWWDLARQTASV	6.7	HLA-DRB1*11:01
**468**	482	PSEQTMRVLPSSAAR	6.7	HLA-DRB1*11:04
**560**	574	PGLLWWDLARQTASV	6.7	HLA-DRB1*11:09
**560**	574	PGLLWWDLARQTASV	6.7	HLA-DRB1*11:10
**230**	244	NLMDFNPDPRSRYTF	6.7	HLA-DRB1*11:11
**560**	574	PGLLWWDLARQTASV	6.7	HLA-DRB1*11:15
**560**	574	PGLLWWDLARQTASV	6.7	HLA-DRB1*11:29
**380**	394	PRKLVIISKGLSLIY	6.8	HLA-DRB1*03:14
**216**	230	PRQVLTLAQNHSRIN	6.8	HLA-DRB1*03:15
**216**	230	PRQVLTLAQNHSRIN	6.8	HLA-DRB1*08:10
**32**	46	LWVGLARGGQRSAED	6.8	HLA-DRB1*11:01
**32**	46	LWVGLARGGQRSAED	6.8	HLA-DRB1*11:09
**32**	46	LWVGLARGGQRSAED	6.8	HLA-DRB1*11:10
**32**	46	LWVGLARGGQRSAED	6.8	HLA-DRB1*11:15
**230**	244	NLMDFNPDPRSRYTF	6.8	HLA-DRB1*11:27
**32**	46	LWVGLARGGQRSAED	6.8	HLA-DRB1*11:29
**51**	65	GRRLAALPVGLSLSA	6.8	HLA-DRB1*11:37
**380**	394	PRKLVIISKGLSLIY	6.9	HLA-DRB1*03:01
**380**	394	PRKLVIISKGLSLIY	6.9	HLA-DRB1*03:06
**380**	394	PRKLVIISKGLSLIY	6.9	HLA-DRB1*03:23
**380**	394	PRKLVIISKGLSLIY	6.9	HLA-DRB1*03:36
**145**	159	TGIVIYAPALILNQV	6.9	HLA-DRB1*08:07
**363**	377	AAVTVEDLIKPRLRS	6.9	HLA-DRB1*11:52
**265**	279	QVQRYVACRTEKQAK	7.0	HLA-DRB1*03:05
**380**	394	PRKLVIISKGLSLIY	7.0	HLA-DRB1*03:40
**355**	369	ASTSINAMAAVTVED	7.0	HLA-DRB1*11:52
**265**	279	QVQRYVACRTEKQAK	7.1	HLA-DRB1*08:04
**225**	239	NHSRINLMDFNPDPR	7.1	HLA-DRB1*08:06
**70**	84	AVQVLGVPSEAYRYG	7.1	HLA-DRB1*08:12
**368**	382	EDLIKPRLRSLAPRK	7.1	HLA-DRB1*11:37

**265**	279	QVQRYVACRTEKQAK	7.1	HLA-DRB1*14:15
**216**	230	PRQVLTLAQNHSRIN	7.2	HLA-DRB1*03:01
**216**	230	PRQVLTLAQNHSRIN	7.2	HLA-DRB1*03:23
**216**	230	PRQVLTLAQNHSRIN	7.2	HLA-DRB1*03:36
**51**	65	GRRLAALPVGLSLSA	7.2	HLA-DRB1*11:01
**51**	65	GRRLAALPVGLSLSA	7.2	HLA-DRB1*11:09
**51**	65	GRRLAALPVGLSLSA	7.2	HLA-DRB1*11:10
**51**	65	GRRLAALPVGLSLSA	7.2	HLA-DRB1*11:15
**51**	65	GRRLAALPVGLSLSA	7.2	HLA-DRB1*11:29
**468**	482	PSEQTMRVLPSSAAR	7.2	HLA-DRB4*01:01
**225**	239	NHSRINLMDFNPDPR	7.3	HLA-DRB1*08:03
**225**	239	NHSRINLMDFNPDPR	7.3	HLA-DRB1*08:14
**27**	41	STGIGLWVGLARGGQ	7.3	HLA-DRB1*11:05
**44**	58	AEDFFTGGRRLAALP	7.3	HLA-DRB1*11:06
**495**	509	DPALLPANDSSRAPS	7.4	HLA-DRB1*03:15
**363**	377	AAVTVEDLIKPRLRS	7.4	HLA-DRB1*08:04
**120**	134	YLEMRFSRAVRLCGT	7.4	HLA-DRB1*11:37
**363**	377	AAVTVEDLIKPRLRS	7.4	HLA-DRB1*14:15
**216**	230	PRQVLTLAQNHSRIN	7.5	HLA-DRB1*03:06
**150**	164	YAPALILNQVTGLDI	7.5	HLA-DRB1*03:40
**610**	624	DRLFFLGQKELEGAG	7.5	HLA-DRB1*08:07
**455**	469	LSLWVALGATLYPPS	7.5	HLA-DRB1*08:07
**265**	279	QVQRYVACRTEKQAK	7.5	HLA-DRB1*11:04
**145**	159	TGIVIYAPALILNQV	7.5	HLA-DRB1*11:14
**145**	159	TGIVIYAPALILNQV	7.5	HLA-DRB1*11:20
**225**	239	NHSRINLMDFNPDPR	7.6	HLA-DRB1*08:12
**216**	230	PRQVLTLAQNHSRIN	7.6	HLA-DRB1*11:05
**355**	369	ASTSINAMAAVTVED	7.7	HLA-DRB1*08:10
**145**	159	TGIVIYAPALILNQV	7.7	HLA-DRB1*08:12
**44**	58	AEDFFTGGRRLAALP	7.7	HLA-DRB1*11:04
**610**	624	DRLFFLGQKELEGAG	7.8	HLA-DRB1*03:05
**216**	230	PRQVLTLAQNHSRIN	7.8	HLA-DRB1*03:40

**327**	341	LLVLDIFEDLPGVPG	7.8	HLA-DRB1*04:05
**216**	230	PRQVLTLAQNHSRIN	7.8	HLA-DRB1*08:06
**209**	223	GVMLVGGPRQVLTLA	7.8	HLA-DRB1*11:13
**145**	159	TGIVIYAPALILNQV	7.8	HLA-DRB1*11:52
**265**	279	QVQRYVACRTEKQAK	7.9	HLA-DRB1*11:06
**468**	482	PSEQTMRVLPSSAAR	8.0	HLA-DRB1*08:02
**468**	482	PSEQTMRVLPSSAAR	8.0	HLA-DRB1*08:09
**145**	159	TGIVIYAPALILNQV	8.0	HLA-DRB1*08:10
**468**	482	PSEQTMRVLPSSAAR	8.0	HLA-DRB1*08:12
**216**	230	PRQVLTLAQNHSRIN	8.0	HLA-DRB1*11:37
**44**	58	AEDFFTGGRRLAALP	8.1	HLA-DRB1*03:40
**120**	134	YLEMRFSRAVRLCGT	8.1	HLA-DRB1*11:05
**120**	134	YLEMRFSRAVRLCGT	8.1	HLA-DRB1*11:08
**578**	592	EEVAILDDNLVKGPE	8.1	HLA-DRB1*11:13
**495**	509	DPALLPANDSSRAPS	8.4	HLA-DRB1*03:07
**44**	58	AEDFFTGGRRLAALP	8.4	HLA-DRB1*03:14
**600**	614	KPPGFLPTNEDRLFF	8.4	HLA-DRB1*08:07
**495**	509	DPALLPANDSSRAPS	8.4	HLA-DRB1*11:07
**44**	58	AEDFFTGGRRLAALP	8.5	HLA-DRB1*11:03
**265**	279	QVQRYVACRTEKQAK	8.5	HLA-DRB1*11:14
**265**	279	QVQRYVACRTEKQAK	8.5	HLA-DRB1*11:20
**363**	377	AAVTVEDLIKPRLRS	8.6	HLA-DRB1*03:01
**363**	377	AAVTVEDLIKPRLRS	8.6	HLA-DRB1*03:23
**363**	377	AAVTVEDLIKPRLRS	8.6	HLA-DRB1*03:36
**225**	239	NHSRINLMDFNPDPR	8.6	HLA-DRB1*04:05
**51**	65	GRRLAALPVGLSLSA	8.6	HLA-DRB1*08:06
**578**	592	EEVAILDDNLVKGPE	8.6	HLA-DRB1*08:12
**560**	574	PGLLWWDLARQTASV	8.6	HLA-DRB1*11:05
**380**	394	PRKLVIISKGLSLIY	8.7	HLA-DRB1*03:05
**216**	230	PRQVLTLAQNHSRIN	8.7	HLA-DRB1*11:01
**363**	377	AAVTVEDLIKPRLRS	8.7	HLA-DRB1*11:02
**363**	377	AAVTVEDLIKPRLRS	8.7	HLA-DRB1*11:03

**216**	230	PRQVLTLAQNHSRIN	8.7	HLA-DRB1*11:08
**216**	230	PRQVLTLAQNHSRIN	8.7	HLA-DRB1*11:09
**216**	230	PRQVLTLAQNHSRIN	8.7	HLA-DRB1*11:10
**216**	230	PRQVLTLAQNHSRIN	8.7	HLA-DRB1*11:15
**363**	377	AAVTVEDLIKPRLRS	8.7	HLA-DRB1*11:16
**51**	65	GRRLAALPVGLSLSA	8.7	HLA-DRB1*11:27
**216**	230	PRQVLTLAQNHSRIN	8.7	HLA-DRB1*11:29
**368**	382	EDLIKPRLRSLAPRK	8.7	HLA-DRB1*11:52
**495**	509	DPALLPANDSSRAPS	8.8	HLA-DRB1*03:06
**257**	271	SMYGVNQAQVQRYVA	8.8	HLA-DRB1*03:40
**368**	382	EDLIKPRLRSLAPRK	8.8	HLA-DRB1*08:06
**44**	58	AEDFFTGGRRLAALP	8.8	HLA-DRB1*11:25
**578**	592	EEVAILDDNLVKGPE	8.8	HLA-DRB1*11:52
**495**	509	DPALLPANDSSRAPS	8.9	HLA-DRB1*03:01
**209**	223	GVMLVGGPRQVLTLA	8.9	HLA-DRB1*03:07
**495**	509	DPALLPANDSSRAPS	8.9	HLA-DRB1*03:23
**495**	509	DPALLPANDSSRAPS	8.9	HLA-DRB1*03:36
**321**	335	PDQYMPLLVLDIFED	8.9	HLA-DRB1*08:07
**468**	482	PSEQTMRVLPSSAAR	8.9	HLA-DRB1*11:05
**209**	223	GVMLVGGPRQVLTLA	8.9	HLA-DRB1*11:07
**216**	230	PRQVLTLAQNHSRIN	8.9	HLA-DRB1*11:11
**27**	41	STGIGLWVGLARGGQ	8.9	HLA-DRB1*11:14
**355**	369	ASTSINAMAAVTVED	8.9	HLA-DRB1*11:14
**216**	230	PRQVLTLAQNHSRIN	8.9	HLA-DRB1*11:19
**120**	134	YLEMRFSRAVRLCGT	8.9	HLA-DRB1*11:19
**27**	41	STGIGLWVGLARGGQ	8.9	HLA-DRB1*11:20
**355**	369	ASTSINAMAAVTVED	8.9	HLA-DRB1*11:20
**51**	65	GRRLAALPVGLSLSA	9.1	HLA-DRB1*04:05
**216**	230	PRQVLTLAQNHSRIN	9.1	HLA-DRB1*08:02
**145**	159	TGIVIYAPALILNQV	9.1	HLA-DRB1*08:03
**216**	230	PRQVLTLAQNHSRIN	9.1	HLA-DRB1*08:09
**145**	159	TGIVIYAPALILNQV	9.1	HLA-DRB1*08:14

**265**	279	QVQRYVACRTEKQAK	9.1	HLA-DRB1*11:25
**1**	15	MEAVETGERPTFGAW	9.2	HLA-DRB1*03:15
**468**	482	PSEQTMRVLPSSAAR	9.2	HLA-DRB1*08:10
**560**	574	PGLLWWDLARQTASV	9.2	HLA-DRB1*11:37
**468**	482	PSEQTMRVLPSSAAR	9.2	HLA-DRB1*11:37
**363**	377	AAVTVEDLIKPRLRS	9.3	HLA-DRB1*03:06
**368**	382	EDLIKPRLRSLAPRK	9.3	HLA-DRB1*08:02
**368**	382	EDLIKPRLRSLAPRK	9.3	HLA-DRB1*08:09
**32**	46	LWVGLARGGQRSAED	9.3	HLA-DRB1*11:05
**578**	592	EEVAILDDNLVKGPE	9.4	HLA-DRB1*08:10
**27**	41	STGIGLWVGLARGGQ	9.5	HLA-DRB1*11:03
**560**	574	PGLLWWDLARQTASV	9.5	HLA-DRB1*11:19
**310**	324	CDPLLLGRISAPDQY	9.6	HLA-DRB1*08:04
**115**	129	TSTYEYLEMRFSRAV	9.6	HLA-DRB1*11:05
**363**	377	AAVTVEDLIKPRLRS	9.6	HLA-DRB1*11:21
**310**	324	CDPLLLGRISAPDQY	9.6	HLA-DRB1*14:15
**216**	230	PRQVLTLAQNHSRIN	9.7	HLA-DRB1*03:14
**495**	509	DPALLPANDSSRAPS	9.8	HLA-DRB1*03:14
**216**	230	PRQVLTLAQNHSRIN	9.8	HLA-DRB1*11:52
**610**	624	DRLFFLGQKELEGAG	9.9	HLA-DRB1*11:11
**468**	482	PSEQTMRVLPSSAAR	9.9	HLA-DRB1*11:25
**225**	239	NHSRINLMDFNPDPR	10.0	HLA-DRB1*08:01
**225**	239	NHSRINLMDFNPDPR	10.0	HLA-DRB1*08:16
**363**	377	AAVTVEDLIKPRLRS	10.0	HLA-DRB1*11:08
**569**	583	RQTASVAPKEEVAIL	10.0	HLA-DRB1*11:52
**265**	279	QVQRYVACRTEKQAK	10.0	HLA-DRB4*01:01
