# Supplementary material for: Predicted T-Cell and B-Cell Epitopes of NIS: Where Do Sjögren’s Syndrome and Hashimoto’s Thyroiditis Converge?
Source: Int J Mol Sci. 2025 Dec 24;27(1):200. doi: 10.3390/ijms27010200 (PMC12785876; doi:10.3390/ijms27010200)
Supplement: Supplementary file 1 [file ijms-27-00200-s001.zip › Table S2 IJMS REV.pdf]

Peptide Start	*Peptide End*	*Peptide Sequence*	*Percentile rank*	*Allele*
**70**	84	PAQLLSFSKLPEPTS	0.03	HLA-DRB1*08:10
**70**	84	PAQLLSFSKLPEPTS	0.03	HLA-DRB1*08:12
**430**	444	ADAVYQEARKVVGAL	0.07	HLA-DRB1*11:01
**430**	444	ADAVYQEARKVVGAL	0.07	HLA-DRB1*11:09
**430**	444	ADAVYQEARKVVGAL	0.07	HLA-DRB1*11:10
**430**	444	ADAVYQEARKVVGAL	0.07	HLA-DRB1*11:15
**430**	444	ADAVYQEARKVVGAL	0.07	HLA-DRB1*11:29
**619**	633	ADKILDLYKHPDNID	0.08	HLA-DRB1*08:06
**494**	508	HATIHPLVRRLDASF	0.08	HLA-DRB1*08:06
**494**	508	HATIHPLVRRLDASF	0.08	HLA-DRB1*08:12
**430**	444	ADAVYQEARKVVGAL	0.08	HLA-DRB1*11:37
**70**	84	PAQLLSFSKLPEPTS	0.09	HLA-DRB1*08:06
**494**	508	HATIHPLVRRLDASF	0.09	HLA-DRB1*08:10
**619**	633	ADKILDLYKHPDNID	0.09	HLA-DRB1*08:10
**619**	633	ADKILDLYKHPDNID	0.09	HLA-DRB1*08:12
**430**	444	ADAVYQEARKVVGAL	0.1	HLA-DRB1*08:02
**430**	444	ADAVYQEARKVVGAL	0.1	HLA-DRB1*08:09
**430**	444	ADAVYQEARKVVGAL	0.15	HLA-DRB1*11:05
**430**	444	ADAVYQEARKVVGAL	0.16	HLA-DRB1*11:08
**52**	66	TAMYATMQRNLKKRG	0.16	HLA-DRB1*11:08
**95**	109	ETSIQAMKRKVNLKT	0.17	HLA-DRB1*11:25
**52**	66	TAMYATMQRNLKKRG	0.18	HLA-DRB1*11:19
**619**	633	ADKILDLYKHPDNID	0.19	HLA-DRB1*08:05
**430**	444	ADAVYQEARKVVGAL	0.19	HLA-DRB1*11:27
**70**	84	PAQLLSFSKLPEPTS	0.2	HLA-DRB1*08:03
**70**	84	PAQLLSFSKLPEPTS	0.2	HLA-DRB1*08:14
**95**	109	ETSIQAMKRKVNLKT	0.2	HLA-DRB1*11:03
**95**	109	ETSIQAMKRKVNLKT	0.22	HLA-DRB1*11:06
**430**	444	ADAVYQEARKVVGAL	0.22	HLA-DRB1*11:19
**95**	109	ETSIQAMKRKVNLKT	0.23	HLA-DRB1*11:04
**619**	633	ADKILDLYKHPDNID	0.24	HLA-DRB1*08:01
**619**	633	ADKILDLYKHPDNID	0.24	HLA-DRB1*08:16
**494**	508	HATIHPLVRRLDASF	0.24	HLA-DRB1*11:06
**494**	508	HATIHPLVRRLDASF	0.26	HLA-DRB1*08:01
**494**	508	HATIHPLVRRLDASF	0.26	HLA-DRB1*08:05
**494**	508	HATIHPLVRRLDASF	0.26	HLA-DRB1*08:16
**494**	508	HATIHPLVRRLDASF	0.27	HLA-DRB1*11:04
**202**	216	LPPVREVTRHVIQVS	0.27	HLA-DRB1*11:52
**95**	109	ETSIQAMKRKVNLKT	0.28	HLA-DRB1*08:31
**494**	508	HATIHPLVRRLDASF	0.28	HLA-DRB1*11:25
**70**	84	PAQLLSFSKLPEPTS	0.33	HLA-DRB1*08:01
**70**	84	PAQLLSFSKLPEPTS	0.33	HLA-DRB1*08:16
**95**	109	ETSIQAMKRKVNLKT	0.34	HLA-DRB1*08:04
**95**	109	ETSIQAMKRKVNLKT	0.34	HLA-DRB1*14:15
**52**	66	TAMYATMQRNLKKRG	0.35	HLA-DRB1*11:05
**360**	374	GRAYLPFVPPRAPSA	0.38	HLA-DRB1*08:02
**360**	374	GRAYLPFVPPRAPSA	0.38	HLA-DRB1*08:09
**494**	508	HATIHPLVRRLDASF	0.39	HLA-DRB1*11:03
**70**	84	PAQLLSFSKLPEPTS	0.42	HLA-DRB1*08:05

**52**	66	TAMYATMQRNLKKRG	0.42	HLA-DRB1*11:27
**95**	109	ETSIQAMKRKVNLKT	0.43	HLA-DRB1*11:11
**95**	109	ETSIQAMKRKVNLKT	0.46	HLA-DRB1*11:05
**202**	216	LPPVREVTRHVIQVS	0.46	HLA-DRB1*11:13
**346**	360	AEGLLRVHARLRDSG	0.46	HLA-DRB1*11:21
**95**	109	ETSIQAMKRKVNLKT	0.46	HLA-DRB1*11:27
**494**	508	HATIHPLVRRLDASF	0.48	HLA-DRB1*08:03
**102**	116	KRKVNLKTQQSQHPT	0.48	HLA-DRB1*08:04
**494**	508	HATIHPLVRRLDASF	0.48	HLA-DRB1*08:14
**95**	109	ETSIQAMKRKVNLKT	0.48	HLA-DRB1*11:01
**95**	109	ETSIQAMKRKVNLKT	0.48	HLA-DRB1*11:09
**95**	109	ETSIQAMKRKVNLKT	0.48	HLA-DRB1*11:10
**95**	109	ETSIQAMKRKVNLKT	0.48	HLA-DRB1*11:15
**95**	109	ETSIQAMKRKVNLKT	0.48	HLA-DRB1*11:29
**102**	116	KRKVNLKTQQSQHPT	0.48	HLA-DRB1*14:15
**95**	109	ETSIQAMKRKVNLKT	0.49	HLA-DRB1*11:37
**346**	360	AEGLLRVHARLRDSG	0.5	HLA-DRB1*11:02
**346**	360	AEGLLRVHARLRDSG	0.5	HLA-DRB1*11:16
**494**	508	HATIHPLVRRLDASF	0.52	HLA-DRB1*08:11
**346**	360	AEGLLRVHARLRDSG	0.52	HLA-DRB1*11:03
**494**	508	HATIHPLVRRLDASF	0.54	HLA-DRB1*08:31
**430**	444	ADAVYQEARKVVGAL	0.56	HLA-DRB1*08:11
**619**	633	ADKILDLYKHPDNID	0.57	HLA-DRB1*08:11
**619**	633	ADKILDLYKHPDNID	0.59	HLA-DRB1*08:03
**619**	633	ADKILDLYKHPDNID	0.59	HLA-DRB1*08:14
**52**	66	TAMYATMQRNLKKRG	0.6	HLA-DRB1*11:01
**95**	109	ETSIQAMKRKVNLKT	0.6	HLA-DRB1*11:08
**52**	66	TAMYATMQRNLKKRG	0.6	HLA-DRB1*11:09
**52**	66	TAMYATMQRNLKKRG	0.6	HLA-DRB1*11:10
**52**	66	TAMYATMQRNLKKRG	0.6	HLA-DRB1*11:15
**52**	66	TAMYATMQRNLKKRG	0.6	HLA-DRB1*11:29
**494**	508	HATIHPLVRRLDASF	0.64	HLA-DRB1*08:04
**95**	109	ETSIQAMKRKVNLKT	0.64	HLA-DRB1*11:02
**95**	109	ETSIQAMKRKVNLKT	0.64	HLA-DRB1*11:16
**494**	508	HATIHPLVRRLDASF	0.64	HLA-DRB1*14:15
**494**	508	HATIHPLVRRLDASF	0.66	HLA-DRB1*11:01
**494**	508	HATIHPLVRRLDASF	0.66	HLA-DRB1*11:09
**494**	508	HATIHPLVRRLDASF	0.66	HLA-DRB1*11:10
**494**	508	HATIHPLVRRLDASF	0.66	HLA-DRB1*11:15
**494**	508	HATIHPLVRRLDASF	0.66	HLA-DRB1*11:29
**70**	84	PAQLLSFSKLPEPTS	0.68	HLA-DRB1*11:02
**70**	84	PAQLLSFSKLPEPTS	0.68	HLA-DRB1*11:16
**95**	109	ETSIQAMKRKVNLKT	0.69	HLA-DRB1*11:19
**360**	374	GRAYLPFVPPRAPSA	0.7	HLA-DRB1*11:05
**494**	508	HATIHPLVRRLDASF	0.7	HLA-DRB1*11:19
**360**	374	GRAYLPFVPPRAPSA	0.71	HLA-DRB1*08:03
**360**	374	GRAYLPFVPPRAPSA	0.71	HLA-DRB1*08:14
**95**	109	ETSIQAMKRKVNLKT	0.71	HLA-DRB1*11:21
**360**	374	GRAYLPFVPPRAPSA	0.72	HLA-DRB1*08:07
**52**	66	TAMYATMQRNLKKRG	0.74	HLA-DRB1*11:11
**70**	84	PAQLLSFSKLPEPTS	0.77	HLA-DRB1*11:03

**494**	508	HATIHPLVRRLDASF	0.79	HLA-DRB1*11:05
**52**	66	TAMYATMQRNLKKRG	0.8	HLA-DRB1*11:37
**494**	508	HATIHPLVRRLDASF	0.81	HLA-DRB1*11:21
**360**	374	GRAYLPFVPPRAPSA	0.82	HLA-DRB1*08:11
**494**	508	HATIHPLVRRLDASF	0.82	HLA-DRB1*11:08
**408**	422	TLWLREHNRLAAALK	0.86	HLA-DRB1*11:03
**95**	109	ETSIQAMKRKVNLKT	0.87	HLA-DRB1*08:06
**532**	546	GGGLDPLIRGLLARP	0.87	HLA-DRB1*11:13
**70**	84	PAQLLSFSKLPEPTS	0.88	HLA-DRB1*08:11
**619**	633	ADKILDLYKHPDNID	0.89	HLA-DRB1*08:31
**430**	444	ADAVYQEARKVVGAL	0.9	HLA-DRB1*08:01
**39**	53	VSSVLEESKRLVDTA	0.9	HLA-DRB1*08:06
**430**	444	ADAVYQEARKVVGAL	0.9	HLA-DRB1*08:16
**202**	216	LPPVREVTRHVIQVS	0.91	HLA-DRB1*11:02
**202**	216	LPPVREVTRHVIQVS	0.91	HLA-DRB1*11:16
**70**	84	PAQLLSFSKLPEPTS	0.93	HLA-DRB1*11:25
**202**	216	LPPVREVTRHVIQVS	0.95	HLA-DRB1*11:21
**95**	109	ETSIQAMKRKVNLKT	0.98	HLA-DRB1*08:02
**95**	109	ETSIQAMKRKVNLKT	0.98	HLA-DRB1*08:09
**494**	508	HATIHPLVRRLDASF	0.98	HLA-DRB1*11:02
**494**	508	HATIHPLVRRLDASF	0.98	HLA-DRB1*11:16
**430**	444	ADAVYQEARKVVGAL	1.0	HLA-DRB1*08:05
**545**	559	RPAKLQVQDQLMNEE	1.0	HLA-DRB4*01:01
**425**	439	NAHWSADAVYQEARK	1.1	HLA-DRB1*03:14
**216**	230	SNEVVTDDDRYSDLL	1.1	HLA-DRB1*03:15
**430**	444	ADAVYQEARKVVGAL	1.1	HLA-DRB1*08:07
**202**	216	LPPVREVTRHVIQVS	1.1	HLA-DRB1*11:03
**430**	444	ADAVYQEARKVVGAL	1.1	HLA-DRB1*11:11
**494**	508	HATIHPLVRRLDASF	1.1	HLA-DRB1*11:11
**95**	109	ETSIQAMKRKVNLKT	1.1	HLA-DRB1*11:13
**408**	422	TLWLREHNRLAAALK	1.1	HLA-DRB1*11:21
**494**	508	HATIHPLVRRLDASF	1.1	HLA-DRB1*11:27
**360**	374	GRAYLPFVPPRAPSA	1.1	HLA-DRB1*11:37
**39**	53	VSSVLEESKRLVDTA	1.1	HLA-DRB1*11:52
**52**	66	TAMYATMQRNLKKRG	1.2	HLA-DRB1*08:02
**52**	66	TAMYATMQRNLKKRG	1.2	HLA-DRB1*08:09
**408**	422	TLWLREHNRLAAALK	1.2	HLA-DRB1*11:02
**619**	633	ADKILDLYKHPDNID	1.2	HLA-DRB1*11:04
**70**	84	PAQLLSFSKLPEPTS	1.2	HLA-DRB1*11:04
**494**	508	HATIHPLVRRLDASF	1.2	HLA-DRB1*11:13
**346**	360	AEGLLRVHARLRDSG	1.2	HLA-DRB1*11:14
**52**	66	TAMYATMQRNLKKRG	1.2	HLA-DRB1*11:14
**408**	422	TLWLREHNRLAAALK	1.2	HLA-DRB1*11:16
**70**	84	PAQLLSFSKLPEPTS	1.2	HLA-DRB1*11:19
**360**	374	GRAYLPFVPPRAPSA	1.2	HLA-DRB1*11:19
**346**	360	AEGLLRVHARLRDSG	1.2	HLA-DRB1*11:20
**52**	66	TAMYATMQRNLKKRG	1.2	HLA-DRB1*11:20
**102**	116	KRKVNLKTQQSQHPT	1.2	HLA-DRB1*11:25
**494**	508	HATIHPLVRRLDASF	1.2	HLA-DRB1*11:37
**151**	165	YRPITGACNNRDHPR	1.3	HLA-DRB1*04:05
**360**	374	GRAYLPFVPPRAPSA	1.3	HLA-DRB1*04:05

**102**	116	KRKVNLKTQQSQHPT	1.3	HLA-DRB1*08:02
**102**	116	KRKVNLKTQQSQHPT	1.3	HLA-DRB1*08:06
**102**	116	KRKVNLKTQQSQHPT	1.3	HLA-DRB1*08:09
**39**	53	VSSVLEESKRLVDTA	1.3	HLA-DRB1*08:10
**202**	216	LPPVREVTRHVIQVS	1.3	HLA-DRB1*08:12
**346**	360	AEGLLRVHARLRDSG	1.3	HLA-DRB1*08:12
**70**	84	PAQLLSFSKLPEPTS	1.3	HLA-DRB1*11:06
**346**	360	AEGLLRVHARLRDSG	1.3	HLA-DRB1*11:11
**39**	53	VSSVLEESKRLVDTA	1.3	HLA-DRB1*11:13
**70**	84	PAQLLSFSKLPEPTS	1.3	HLA-DRB1*11:21
**202**	216	LPPVREVTRHVIQVS	1.3	HLA-DRB1*11:25
**532**	546	GGGLDPLIRGLLARP	1.3	HLA-DRB1*11:52
**425**	439	NAHWSADAVYQEARK	1.4	HLA-DRB1*03:05
**327**	341	STVYGSSPALERQLR	1.4	HLA-DRB1*03:40
**70**	84	PAQLLSFSKLPEPTS	1.4	HLA-DRB1*08:04
**346**	360	AEGLLRVHARLRDSG	1.4	HLA-DRB1*08:10
**39**	53	VSSVLEESKRLVDTA	1.4	HLA-DRB1*08:12
**70**	84	PAQLLSFSKLPEPTS	1.4	HLA-DRB1*08:31
**70**	84	PAQLLSFSKLPEPTS	1.4	HLA-DRB1*11:13
**70**	84	PAQLLSFSKLPEPTS	1.4	HLA-DRB1*14:15
**216**	230	SNEVVTDDDRYSDLL	1.5	HLA-DRB1*03:06
**388**	402	PCFLAGDGRATEVPS	1.5	HLA-DRB1*03:15
**425**	439	NAHWSADAVYQEARK	1.5	HLA-DRB1*04:05
**619**	633	ADKILDLYKHPDNID	1.5	HLA-DRB1*11:06
**360**	374	GRAYLPFVPPRAPSA	1.5	HLA-DRB1*11:27
**95**	109	ETSIQAMKRKVNLKT	1.5	HLA-DRB1*11:52
**314**	328	RQQMNGLTSFLDAST	1.6	HLA-DRB1*04:05
**619**	633	ADKILDLYKHPDNID	1.6	HLA-DRB1*08:04
**102**	116	KRKVNLKTQQSQHPT	1.6	HLA-DRB1*08:31
**70**	84	PAQLLSFSKLPEPTS	1.6	HLA-DRB1*11:08
**674**	688	NSHVFTDAQRRELEK	1.6	HLA-DRB1*11:08
**360**	374	GRAYLPFVPPRAPSA	1.6	HLA-DRB1*11:08
**674**	688	NSHVFTDAQRRELEK	1.6	HLA-DRB1*11:11
**619**	633	ADKILDLYKHPDNID	1.6	HLA-DRB1*11:25
**353**	367	HARLRDSGRAYLPFV	1.6	HLA-DRB1*11:52
**619**	633	ADKILDLYKHPDNID	1.6	HLA-DRB1*14:15
**216**	230	SNEVVTDDDRYSDLL	1.7	HLA-DRB1*03:01
**327**	341	STVYGSSPALERQLR	1.7	HLA-DRB1*03:14
**216**	230	SNEVVTDDDRYSDLL	1.7	HLA-DRB1*03:23
**216**	230	SNEVVTDDDRYSDLL	1.7	HLA-DRB1*03:36
**39**	53	VSSVLEESKRLVDTA	1.7	HLA-DRB1*08:01
**360**	374	GRAYLPFVPPRAPSA	1.7	HLA-DRB1*08:01
**494**	508	HATIHPLVRRLDASF	1.7	HLA-DRB1*08:02
**494**	508	HATIHPLVRRLDASF	1.7	HLA-DRB1*08:09
**39**	53	VSSVLEESKRLVDTA	1.7	HLA-DRB1*08:16
**360**	374	GRAYLPFVPPRAPSA	1.7	HLA-DRB1*08:16
**202**	216	LPPVREVTRHVIQVS	1.7	HLA-DRB1*11:04
**202**	216	LPPVREVTRHVIQVS	1.7	HLA-DRB1*11:06
**408**	422	TLWLREHNRLAAALK	1.7	HLA-DRB1*11:11
**202**	216	LPPVREVTRHVIQVS	1.8	HLA-DRB1*08:10
**594**	608	WREFCGLPRLETPAD	1.8	HLA-DRB1*11:01

**360**	374	GRAYLPFVPPRAPSA	1.8	HLA-DRB1*11:01
**594**	608	WREFCGLPRLETPAD	1.8	HLA-DRB1*11:09
**360**	374	GRAYLPFVPPRAPSA	1.8	HLA-DRB1*11:09
**594**	608	WREFCGLPRLETPAD	1.8	HLA-DRB1*11:10
**360**	374	GRAYLPFVPPRAPSA	1.8	HLA-DRB1*11:10
**95**	109	ETSIQAMKRKVNLKT	1.8	HLA-DRB1*11:14
**594**	608	WREFCGLPRLETPAD	1.8	HLA-DRB1*11:15
**360**	374	GRAYLPFVPPRAPSA	1.8	HLA-DRB1*11:15
**95**	109	ETSIQAMKRKVNLKT	1.8	HLA-DRB1*11:20
**594**	608	WREFCGLPRLETPAD	1.8	HLA-DRB1*11:29
**360**	374	GRAYLPFVPPRAPSA	1.8	HLA-DRB1*11:29
**388**	402	PCFLAGDGRATEVPS	1.9	HLA-DRB1*03:01
**39**	53	VSSVLEESKRLVDTA	1.9	HLA-DRB1*03:07
**674**	688	NSHVFTDAQRRELEK	1.9	HLA-DRB1*03:15
**388**	402	PCFLAGDGRATEVPS	1.9	HLA-DRB1*03:23
**388**	402	PCFLAGDGRATEVPS	1.9	HLA-DRB1*03:36
**95**	109	ETSIQAMKRKVNLKT	1.9	HLA-DRB1*08:05
**39**	53	VSSVLEESKRLVDTA	1.9	HLA-DRB1*11:07
**360**	374	GRAYLPFVPPRAPSA	1.9	HLA-DRB1*11:11
**202**	216	LPPVREVTRHVIQVS	1.9	HLA-DRB1*11:14
**674**	688	NSHVFTDAQRRELEK	1.9	HLA-DRB1*11:19
**202**	216	LPPVREVTRHVIQVS	1.9	HLA-DRB1*11:20
**594**	608	WREFCGLPRLETPAD	1.9	HLA-DRB1*11:27
**594**	608	WREFCGLPRLETPAD	1.9	HLA-DRB1*11:37
**570**	584	SSTLDLASINLQRGR	1.9	HLA-DRB4*01:01
**674**	688	NSHVFTDAQRRELEK	2.0	HLA-DRB1*03:06
**540**	554	RGLLARPAKLQVQDQ	2.0	HLA-DRB1*08:04
**202**	216	LPPVREVTRHVIQVS	2.0	HLA-DRB1*08:06
**674**	688	NSHVFTDAQRRELEK	2.0	HLA-DRB1*08:11
**532**	546	GGGLDPLIRGLLARP	2.0	HLA-DRB1*11:04
**70**	84	PAQLLSFSKLPEPTS	2.0	HLA-DRB1*11:05
**594**	608	WREFCGLPRLETPAD	2.0	HLA-DRB1*11:05
**540**	554	RGLLARPAKLQVQDQ	2.0	HLA-DRB1*14:15
**388**	402	PCFLAGDGRATEVPS	2.1	HLA-DRB1*03:14
**39**	53	VSSVLEESKRLVDTA	2.1	HLA-DRB1*03:15
**239**	253	HDIAFTPQSTSKAAF	2.1	HLA-DRB1*04:05
**413**	427	EHNRLAAALKALNAH	2.1	HLA-DRB1*08:04
**39**	53	VSSVLEESKRLVDTA	2.1	HLA-DRB1*08:05
**52**	66	TAMYATMQRNLKKRG	2.1	HLA-DRB1*08:05
**360**	374	GRAYLPFVPPRAPSA	2.1	HLA-DRB1*08:05
**346**	360	AEGLLRVHARLRDSG	2.1	HLA-DRB1*08:06
**95**	109	ETSIQAMKRKVNLKT	2.1	HLA-DRB1*08:10
**95**	109	ETSIQAMKRKVNLKT	2.1	HLA-DRB1*08:12
**102**	116	KRKVNLKTQQSQHPT	2.1	HLA-DRB1*11:03
**70**	84	PAQLLSFSKLPEPTS	2.1	HLA-DRB1*11:11
**532**	546	GGGLDPLIRGLLARP	2.1	HLA-DRB1*11:25
**494**	508	HATIHPLVRRLDASF	2.1	HLA-DRB1*11:52
**413**	427	EHNRLAAALKALNAH	2.1	HLA-DRB1*14:15
**674**	688	NSHVFTDAQRRELEK	2.2	HLA-DRB1*03:01
**327**	341	STVYGSSPALERQLR	2.2	HLA-DRB1*03:05
**216**	230	SNEVVTDDDRYSDLL	2.2	HLA-DRB1*03:14

**674**	688	NSHVFTDAQRRELEK	2.2	HLA-DRB1*03:23
**674**	688	NSHVFTDAQRRELEK	2.2	HLA-DRB1*03:36
**209**	223	TRHVIQVSNEVVTDD	2.2	HLA-DRB1*08:07
**674**	688	NSHVFTDAQRRELEK	2.2	HLA-DRB1*11:01
**619**	633	ADKILDLYKHPDNID	2.2	HLA-DRB1*11:05
**674**	688	NSHVFTDAQRRELEK	2.2	HLA-DRB1*11:09
**674**	688	NSHVFTDAQRRELEK	2.2	HLA-DRB1*11:10
**202**	216	LPPVREVTRHVIQVS	2.2	HLA-DRB1*11:11
**430**	444	ADAVYQEARKVVGAL	2.2	HLA-DRB1*11:13
**674**	688	NSHVFTDAQRRELEK	2.2	HLA-DRB1*11:15
**674**	688	NSHVFTDAQRRELEK	2.2	HLA-DRB1*11:29
**674**	688	NSHVFTDAQRRELEK	2.2	HLA-DRB1*11:37
**209**	223	TRHVIQVSNEVVTDD	2.2	HLA-DRB1*11:52
**430**	444	ADAVYQEARKVVGAL	2.3	HLA-DRB1*08:03
**430**	444	ADAVYQEARKVVGAL	2.3	HLA-DRB1*08:14
**674**	688	NSHVFTDAQRRELEK	2.3	HLA-DRB1*11:27
**388**	402	PCFLAGDGRATEVPS	2.4	HLA-DRB1*03:06
**413**	427	EHNRLAAALKALNAH	2.4	HLA-DRB1*08:02
**413**	427	EHNRLAAALKALNAH	2.4	HLA-DRB1*08:09
**102**	116	KRKVNLKTQQSQHPT	2.4	HLA-DRB1*08:11
**532**	546	GGGLDPLIRGLLARP	2.4	HLA-DRB1*11:06
**594**	608	WREFCGLPRLETPAD	2.4	HLA-DRB1*11:08
**70**	84	PAQLLSFSKLPEPTS	2.4	HLA-DRB1*11:14
**70**	84	PAQLLSFSKLPEPTS	2.4	HLA-DRB1*11:20
**691**	705	LSRVICDNTGLTRVP	2.5	HLA-DRB1*03:06
**674**	688	NSHVFTDAQRRELEK	2.5	HLA-DRB1*03:14
**95**	109	ETSIQAMKRKVNLKT	2.5	HLA-DRB1*08:01
**239**	253	HDIAFTPQSTSKAAF	2.5	HLA-DRB1*08:02
**239**	253	HDIAFTPQSTSKAAF	2.5	HLA-DRB1*08:09
**102**	116	KRKVNLKTQQSQHPT	2.5	HLA-DRB1*08:10
**95**	109	ETSIQAMKRKVNLKT	2.5	HLA-DRB1*08:16
**408**	422	TLWLREHNRLAAALK	2.5	HLA-DRB1*11:14
**408**	422	TLWLREHNRLAAALK	2.5	HLA-DRB1*11:20
**761**	775	RRVLVYSCRHGYELQ	2.5	HLA-DRB1*11:21
**413**	427	EHNRLAAALKALNAH	2.5	HLA-DRB1*11:25
**413**	427	EHNRLAAALKALNAH	2.5	HLA-DRB1*11:52
**425**	439	NAHWSADAVYQEARK	2.6	HLA-DRB1*03:40
**52**	66	TAMYATMQRNLKKRG	2.6	HLA-DRB1*08:03
**52**	66	TAMYATMQRNLKKRG	2.6	HLA-DRB1*08:14
**413**	427	EHNRLAAALKALNAH	2.6	HLA-DRB1*08:31
**619**	633	ADKILDLYKHPDNID	2.6	HLA-DRB1*11:01
**70**	84	PAQLLSFSKLPEPTS	2.6	HLA-DRB1*11:01
**39**	53	VSSVLEESKRLVDTA	2.6	HLA-DRB1*11:02
**619**	633	ADKILDLYKHPDNID	2.6	HLA-DRB1*11:03
**619**	633	ADKILDLYKHPDNID	2.6	HLA-DRB1*11:08
**619**	633	ADKILDLYKHPDNID	2.6	HLA-DRB1*11:09
**70**	84	PAQLLSFSKLPEPTS	2.6	HLA-DRB1*11:09
**619**	633	ADKILDLYKHPDNID	2.6	HLA-DRB1*11:10
**70**	84	PAQLLSFSKLPEPTS	2.6	HLA-DRB1*11:10
**619**	633	ADKILDLYKHPDNID	2.6	HLA-DRB1*11:15
**70**	84	PAQLLSFSKLPEPTS	2.6	HLA-DRB1*11:15

**39**	53	VSSVLEESKRLVDTA	2.6	HLA-DRB1*11:16
**39**	53	VSSVLEESKRLVDTA	2.6	HLA-DRB1*11:21
**619**	633	ADKILDLYKHPDNID	2.6	HLA-DRB1*11:29
**70**	84	PAQLLSFSKLPEPTS	2.6	HLA-DRB1*11:29
**216**	230	SNEVVTDDDRYSDLL	2.7	HLA-DRB1*03:07
**202**	216	LPPVREVTRHVIQVS	2.7	HLA-DRB1*08:07
**674**	688	NSHVFTDAQRRELEK	2.7	HLA-DRB1*08:07
**413**	427	EHNRLAAALKALNAH	2.7	HLA-DRB1*11:04
**216**	230	SNEVVTDDDRYSDLL	2.7	HLA-DRB1*11:07
**594**	608	WREFCGLPRLETPAD	2.7	HLA-DRB1*11:19
**430**	444	ADAVYQEARKVVGAL	2.7	HLA-DRB1*11:52
**619**	633	ADKILDLYKHPDNID	2.8	HLA-DRB1*08:07
**202**	216	LPPVREVTRHVIQVS	2.8	HLA-DRB1*08:11
**532**	546	GGGLDPLIRGLLARP	2.8	HLA-DRB1*08:31
**39**	53	VSSVLEESKRLVDTA	2.8	HLA-DRB1*11:03
**674**	688	NSHVFTDAQRRELEK	2.8	HLA-DRB1*11:05
**767**	781	SCRHGYELQGREQLT	2.8	HLA-DRB1*11:08
**346**	360	AEGLLRVHARLRDSG	2.8	HLA-DRB1*11:25
**413**	427	EHNRLAAALKALNAH	2.8	HLA-DRB1*11:27
**70**	84	PAQLLSFSKLPEPTS	2.8	HLA-DRB1*11:52
**239**	253	HDIAFTPQSTSKAAF	2.8	HLA-DRB4*01:01
**202**	216	LPPVREVTRHVIQVS	2.9	HLA-DRB1*08:04
**413**	427	EHNRLAAALKALNAH	2.9	HLA-DRB1*08:06
**95**	109	ETSIQAMKRKVNLKT	2.9	HLA-DRB1*08:11
**346**	360	AEGLLRVHARLRDSG	2.9	HLA-DRB1*11:13
**619**	633	ADKILDLYKHPDNID	2.9	HLA-DRB1*11:19
**202**	216	LPPVREVTRHVIQVS	2.9	HLA-DRB1*14:15
**691**	705	LSRVICDNTGLTRVP	3.0	HLA-DRB1*03:01
**388**	402	PCFLAGDGRATEVPS	3.0	HLA-DRB1*03:07
**691**	705	LSRVICDNTGLTRVP	3.0	HLA-DRB1*03:23
**691**	705	LSRVICDNTGLTRVP	3.0	HLA-DRB1*03:36
**52**	66	TAMYATMQRNLKKRG	3.0	HLA-DRB1*08:01
**39**	53	VSSVLEESKRLVDTA	3.0	HLA-DRB1*08:03
**532**	546	GGGLDPLIRGLLARP	3.0	HLA-DRB1*08:06
**39**	53	VSSVLEESKRLVDTA	3.0	HLA-DRB1*08:14
**52**	66	TAMYATMQRNLKKRG	3.0	HLA-DRB1*08:16
**102**	116	KRKVNLKTQQSQHPT	3.0	HLA-DRB1*11:02
**761**	775	RRVLVYSCRHGYELQ	3.0	HLA-DRB1*11:02
**430**	444	ADAVYQEARKVVGAL	3.0	HLA-DRB1*11:04
**413**	427	EHNRLAAALKALNAH	3.0	HLA-DRB1*11:06
**388**	402	PCFLAGDGRATEVPS	3.0	HLA-DRB1*11:07
**102**	116	KRKVNLKTQQSQHPT	3.0	HLA-DRB1*11:16
**761**	775	RRVLVYSCRHGYELQ	3.0	HLA-DRB1*11:16
**202**	216	LPPVREVTRHVIQVS	3.0	HLA-DRB1*11:19
**413**	427	EHNRLAAALKALNAH	3.0	HLA-DRB1*11:37
**698**	712	NTGLTRVPMDAFQVG	3.0	HLA-DRB4*01:01
**216**	230	SNEVVTDDDRYSDLL	3.1	HLA-DRB1*03:05
**674**	688	NSHVFTDAQRRELEK	3.1	HLA-DRB1*03:07
**430**	444	ADAVYQEARKVVGAL	3.1	HLA-DRB1*11:06
**346**	360	AEGLLRVHARLRDSG	3.1	HLA-DRB1*11:06
**39**	53	VSSVLEESKRLVDTA	3.1	HLA-DRB1*11:06

**674**	688	NSHVFTDAQRRELEK	3.1	HLA-DRB1*11:07
**360**	374	GRAYLPFVPPRAPSA	3.1	HLA-DRB1*11:14
**494**	508	HATIHPLVRRLDASF	3.1	HLA-DRB1*11:14
**430**	444	ADAVYQEARKVVGAL	3.1	HLA-DRB1*11:14
**674**	688	NSHVFTDAQRRELEK	3.1	HLA-DRB1*11:14
**360**	374	GRAYLPFVPPRAPSA	3.1	HLA-DRB1*11:20
**494**	508	HATIHPLVRRLDASF	3.1	HLA-DRB1*11:20
**430**	444	ADAVYQEARKVVGAL	3.1	HLA-DRB1*11:20
**674**	688	NSHVFTDAQRRELEK	3.1	HLA-DRB1*11:20
**619**	633	ADKILDLYKHPDNID	3.1	HLA-DRB1*11:37
**102**	116	KRKVNLKTQQSQHPT	3.2	HLA-DRB1*08:01
**102**	116	KRKVNLKTQQSQHPT	3.2	HLA-DRB1*08:16
**39**	53	VSSVLEESKRLVDTA	3.2	HLA-DRB1*11:04
**767**	781	SCRHGYELQGREQLT	3.2	HLA-DRB1*11:19
**430**	444	ADAVYQEARKVVGAL	3.2	HLA-DRB1*11:25
**70**	84	PAQLLSFSKLPEPTS	3.2	HLA-DRB1*11:27
**202**	216	LPPVREVTRHVIQVS	3.3	HLA-DRB1*08:31
**413**	427	EHNRLAAALKALNAH	3.3	HLA-DRB1*11:01
**102**	116	KRKVNLKTQQSQHPT	3.3	HLA-DRB1*11:04
**202**	216	LPPVREVTRHVIQVS	3.3	HLA-DRB1*11:08
**413**	427	EHNRLAAALKALNAH	3.3	HLA-DRB1*11:09
**413**	427	EHNRLAAALKALNAH	3.3	HLA-DRB1*11:10
**413**	427	EHNRLAAALKALNAH	3.3	HLA-DRB1*11:15
**413**	427	EHNRLAAALKALNAH	3.3	HLA-DRB1*11:29
**761**	775	RRVLVYSCRHGYELQ	3.4	HLA-DRB1*11:14
**761**	775	RRVLVYSCRHGYELQ	3.4	HLA-DRB1*11:20
**540**	554	RGLLARPAKLQVQDQ	3.4	HLA-DRB1*11:25
**239**	253	HDIAFTPQSTSKAAF	3.4	HLA-DRB1*11:37
**388**	402	PCFLAGDGRATEVPS	3.5	HLA-DRB1*03:05
**674**	688	NSHVFTDAQRRELEK	3.5	HLA-DRB1*08:02
**532**	546	GGGLDPLIRGLLARP	3.5	HLA-DRB1*08:04
**674**	688	NSHVFTDAQRRELEK	3.5	HLA-DRB1*08:09
**346**	360	AEGLLRVHARLRDSG	3.5	HLA-DRB1*11:04
**532**	546	GGGLDPLIRGLLARP	3.5	HLA-DRB1*14:15
**674**	688	NSHVFTDAQRRELEK	3.6	HLA-DRB1*03:05
**102**	116	KRKVNLKTQQSQHPT	3.6	HLA-DRB1*08:12
**430**	444	ADAVYQEARKVVGAL	3.6	HLA-DRB1*08:31
**346**	360	AEGLLRVHARLRDSG	3.6	HLA-DRB1*11:19
**70**	84	PAQLLSFSKLPEPTS	3.6	HLA-DRB1*11:37
**532**	546	GGGLDPLIRGLLARP	3.7	HLA-DRB1*11:01
**430**	444	ADAVYQEARKVVGAL	3.7	HLA-DRB1*11:03
**532**	546	GGGLDPLIRGLLARP	3.7	HLA-DRB1*11:03
**532**	546	GGGLDPLIRGLLARP	3.7	HLA-DRB1*11:09
**532**	546	GGGLDPLIRGLLARP	3.7	HLA-DRB1*11:10
**19**	33	FFPFISRGKELLWGK	3.7	HLA-DRB1*11:11
**532**	546	GGGLDPLIRGLLARP	3.7	HLA-DRB1*11:15
**532**	546	GGGLDPLIRGLLARP	3.7	HLA-DRB1*11:29
**691**	705	LSRVICDNTGLTRVP	3.8	HLA-DRB1*03:07
**70**	84	PAQLLSFSKLPEPTS	3.8	HLA-DRB1*08:02
**70**	84	PAQLLSFSKLPEPTS	3.8	HLA-DRB1*08:09
**209**	223	TRHVIQVSNEVVTDD	3.8	HLA-DRB1*08:12

**430**	444	ADAVYQEARKVVGAL	3.8	HLA-DRB1*11:02
**691**	705	LSRVICDNTGLTRVP	3.8	HLA-DRB1*11:07
**619**	633	ADKILDLYKHPDNID	3.8	HLA-DRB1*11:13
**430**	444	ADAVYQEARKVVGAL	3.8	HLA-DRB1*11:16
**767**	781	SCRHGYELQGREQLT	3.8	HLA-DRB1*11:27
**767**	781	SCRHGYELQGREQLT	3.9	HLA-DRB1*03:14
**619**	633	ADKILDLYKHPDNID	3.9	HLA-DRB1*08:02
**346**	360	AEGLLRVHARLRDSG	3.9	HLA-DRB1*08:05
**619**	633	ADKILDLYKHPDNID	3.9	HLA-DRB1*08:09
**413**	427	EHNRLAAALKALNAH	3.9	HLA-DRB1*11:05
**102**	116	KRKVNLKTQQSQHPT	3.9	HLA-DRB1*11:06
**19**	33	FFPFISRGKELLWGK	3.9	HLA-DRB1*11:14
**19**	33	FFPFISRGKELLWGK	3.9	HLA-DRB1*11:20
**654**	668	ACLIGKQMKALRDGD	3.9	HLA-DRB1*11:21
**594**	608	WREFCGLPRLETPAD	4.0	HLA-DRB1*08:02
**540**	554	RGLLARPAKLQVQDQ	4.0	HLA-DRB1*08:02
**346**	360	AEGLLRVHARLRDSG	4.0	HLA-DRB1*08:03
**594**	608	WREFCGLPRLETPAD	4.0	HLA-DRB1*08:05
**594**	608	WREFCGLPRLETPAD	4.0	HLA-DRB1*08:09
**540**	554	RGLLARPAKLQVQDQ	4.0	HLA-DRB1*08:09
**346**	360	AEGLLRVHARLRDSG	4.0	HLA-DRB1*08:14
**39**	53	VSSVLEESKRLVDTA	4.0	HLA-DRB1*08:31
**418**	432	AAALKALNAHWSADA	4.0	HLA-DRB1*08:31
**202**	216	LPPVREVTRHVIQVS	4.0	HLA-DRB1*11:01
**102**	116	KRKVNLKTQQSQHPT	4.0	HLA-DRB1*11:05
**202**	216	LPPVREVTRHVIQVS	4.0	HLA-DRB1*11:09
**202**	216	LPPVREVTRHVIQVS	4.0	HLA-DRB1*11:10
**202**	216	LPPVREVTRHVIQVS	4.0	HLA-DRB1*11:15
**39**	53	VSSVLEESKRLVDTA	4.0	HLA-DRB1*11:25
**202**	216	LPPVREVTRHVIQVS	4.0	HLA-DRB1*11:29
**532**	546	GGGLDPLIRGLLARP	4.0	HLA-DRB1*11:37
**39**	53	VSSVLEESKRLVDTA	4.1	HLA-DRB1*03:01
**691**	705	LSRVICDNTGLTRVP	4.1	HLA-DRB1*03:05
**39**	53	VSSVLEESKRLVDTA	4.1	HLA-DRB1*03:23
**39**	53	VSSVLEESKRLVDTA	4.1	HLA-DRB1*03:36
**674**	688	NSHVFTDAQRRELEK	4.1	HLA-DRB1*03:40
**674**	688	NSHVFTDAQRRELEK	4.1	HLA-DRB1*08:01
**346**	360	AEGLLRVHARLRDSG	4.1	HLA-DRB1*08:01
**674**	688	NSHVFTDAQRRELEK	4.1	HLA-DRB1*08:05
**52**	66	TAMYATMQRNLKKRG	4.1	HLA-DRB1*08:11
**674**	688	NSHVFTDAQRRELEK	4.1	HLA-DRB1*08:16
**346**	360	AEGLLRVHARLRDSG	4.1	HLA-DRB1*08:16
**654**	668	ACLIGKQMKALRDGD	4.1	HLA-DRB1*11:02
**346**	360	AEGLLRVHARLRDSG	4.1	HLA-DRB1*11:08
**532**	546	GGGLDPLIRGLLARP	4.1	HLA-DRB1*11:08
**654**	668	ACLIGKQMKALRDGD	4.1	HLA-DRB1*11:16
**102**	116	KRKVNLKTQQSQHPT	4.1	HLA-DRB1*11:21
**619**	633	ADKILDLYKHPDNID	4.1	HLA-DRB1*11:27
**767**	781	SCRHGYELQGREQLT	4.1	HLA-DRB1*11:37
**39**	53	VSSVLEESKRLVDTA	4.2	HLA-DRB1*08:11
**408**	422	TLWLREHNRLAAALK	4.2	HLA-DRB1*11:25

**346**	360	AEGLLRVHARLRDSG	4.2	HLA-DRB1*11:52
**102**	116	KRKVNLKTQQSQHPT	4.2	HLA-DRB4*01:01
**340**	354	LRNWTSAEGLLRVHA	4.3	HLA-DRB1*03:40
**413**	427	EHNRLAAALKALNAH	4.3	HLA-DRB1*08:01
**532**	546	GGGLDPLIRGLLARP	4.3	HLA-DRB1*08:10
**413**	427	EHNRLAAALKALNAH	4.3	HLA-DRB1*08:16
**619**	633	ADKILDLYKHPDNID	4.3	HLA-DRB1*11:02
**143**	157	PNTCLANKYRPITGA	4.3	HLA-DRB1*11:03
**540**	554	RGLLARPAKLQVQDQ	4.3	HLA-DRB1*11:03
**532**	546	GGGLDPLIRGLLARP	4.3	HLA-DRB1*11:05
**413**	427	EHNRLAAALKALNAH	4.3	HLA-DRB1*11:08
**39**	53	VSSVLEESKRLVDTA	4.3	HLA-DRB1*11:08
**39**	53	VSSVLEESKRLVDTA	4.3	HLA-DRB1*11:14
**619**	633	ADKILDLYKHPDNID	4.3	HLA-DRB1*11:16
**39**	53	VSSVLEESKRLVDTA	4.3	HLA-DRB1*11:19
**39**	53	VSSVLEESKRLVDTA	4.3	HLA-DRB1*11:20
**52**	66	TAMYATMQRNLKKRG	4.4	HLA-DRB1*03:14
**691**	705	LSRVICDNTGLTRVP	4.4	HLA-DRB1*03:15
**209**	223	TRHVIQVSNEVVTDD	4.4	HLA-DRB1*08:10
**532**	546	GGGLDPLIRGLLARP	4.4	HLA-DRB1*08:12
**532**	546	GGGLDPLIRGLLARP	4.4	HLA-DRB1*11:19
**202**	216	LPPVREVTRHVIQVS	4.4	HLA-DRB1*11:27
**39**	53	VSSVLEESKRLVDTA	4.5	HLA-DRB1*03:06
**83**	97	TSGVIARAAEIMETS	4.5	HLA-DRB1*08:12
**532**	546	GGGLDPLIRGLLARP	4.5	HLA-DRB1*11:27
**430**	444	ADAVYQEARKVVGAL	4.7	HLA-DRB1*08:04
**767**	781	SCRHGYELQGREQLT	4.7	HLA-DRB1*11:01
**202**	216	LPPVREVTRHVIQVS	4.7	HLA-DRB1*11:05
**767**	781	SCRHGYELQGREQLT	4.7	HLA-DRB1*11:09
**767**	781	SCRHGYELQGREQLT	4.7	HLA-DRB1*11:10
**102**	116	KRKVNLKTQQSQHPT	4.7	HLA-DRB1*11:11
**767**	781	SCRHGYELQGREQLT	4.7	HLA-DRB1*11:15
**413**	427	EHNRLAAALKALNAH	4.7	HLA-DRB1*11:19
**430**	444	ADAVYQEARKVVGAL	4.7	HLA-DRB1*11:21
**767**	781	SCRHGYELQGREQLT	4.7	HLA-DRB1*11:29
**430**	444	ADAVYQEARKVVGAL	4.7	HLA-DRB1*14:15
**430**	444	ADAVYQEARKVVGAL	4.8	HLA-DRB1*03:07
**761**	775	RRVLVYSCRHGYELQ	4.8	HLA-DRB1*11:03
**430**	444	ADAVYQEARKVVGAL	4.8	HLA-DRB1*11:07
**39**	53	VSSVLEESKRLVDTA	4.8	HLA-DRB1*11:27
**691**	705	LSRVICDNTGLTRVP	4.9	HLA-DRB1*03:14
**39**	53	VSSVLEESKRLVDTA	4.9	HLA-DRB1*03:40
**52**	66	TAMYATMQRNLKKRG	4.9	HLA-DRB1*03:40
**52**	66	TAMYATMQRNLKKRG	4.9	HLA-DRB1*11:04
**619**	633	ADKILDLYKHPDNID	4.9	HLA-DRB1*11:11
**209**	223	TRHVIQVSNEVVTDD	4.9	HLA-DRB1*11:13
**430**	444	ADAVYQEARKVVGAL	5.0	HLA-DRB1*03:14
**418**	432	AAALKALNAHWSADA	5.0	HLA-DRB1*08:04
**70**	84	PAQLLSFSKLPEPTS	5.0	HLA-DRB1*08:07
**418**	432	AAALKALNAHWSADA	5.0	HLA-DRB1*14:15
**388**	402	PCFLAGDGRATEVPS	5.1	HLA-DRB1*03:40

**691**	705	LSRVICDNTGLTRVP	5.1	HLA-DRB1*03:40
**39**	53	VSSVLEESKRLVDTA	5.1	HLA-DRB1*08:04
**489**	503	AFRFGHATIHPLVRR	5.1	HLA-DRB1*08:07
**39**	53	VSSVLEESKRLVDTA	5.1	HLA-DRB1*14:15
**430**	444	ADAVYQEARKVVGAL	5.2	HLA-DRB1*03:40
**767**	781	SCRHGYELQGREQLT	5.2	HLA-DRB1*03:40
**674**	688	NSHVFTDAQRRELEK	5.2	HLA-DRB1*08:03
**674**	688	NSHVFTDAQRRELEK	5.2	HLA-DRB1*08:14
**532**	546	GGGLDPLIRGLLARP	5.2	HLA-DRB1*11:02
**540**	554	RGLLARPAKLQVQDQ	5.2	HLA-DRB1*11:02
**239**	253	HDIAFTPQSTSKAAF	5.2	HLA-DRB1*11:05
**532**	546	GGGLDPLIRGLLARP	5.2	HLA-DRB1*11:16
**540**	554	RGLLARPAKLQVQDQ	5.2	HLA-DRB1*11:16
**674**	688	NSHVFTDAQRRELEK	5.2	HLA-DRB1*11:52
**425**	439	NAHWSADAVYQEARK	5.3	HLA-DRB1*03:06
**346**	360	AEGLLRVHARLRDSG	5.3	HLA-DRB1*03:07
**413**	427	EHNRLAAALKALNAH	5.3	HLA-DRB1*08:10
**418**	432	AAALKALNAHWSADA	5.3	HLA-DRB1*11:06
**346**	360	AEGLLRVHARLRDSG	5.3	HLA-DRB1*11:07
**143**	157	PNTCLANKYRPITGA	5.3	HLA-DRB1*11:11
**216**	230	SNEVVTDDDRYSDLL	5.4	HLA-DRB1*03:40
**540**	554	RGLLARPAKLQVQDQ	5.4	HLA-DRB1*11:21
**340**	354	LRNWTSAEGLLRVHA	5.5	HLA-DRB1*03:05
**39**	53	VSSVLEESKRLVDTA	5.5	HLA-DRB1*03:14
**83**	97	TSGVIARAAEIMETS	5.5	HLA-DRB1*08:10
**619**	633	ADKILDLYKHPDNID	5.5	HLA-DRB1*11:21
**239**	253	HDIAFTPQSTSKAAF	5.5	HLA-DRB1*11:27
**346**	360	AEGLLRVHARLRDSG	5.5	HLA-DRB1*11:27
**39**	53	VSSVLEESKRLVDTA	5.5	HLA-DRB1*11:37
**437**	451	ARKVVGALHQIITLR	5.5	HLA-DRB1*11:52
**52**	66	TAMYATMQRNLKKRG	5.6	HLA-DRB1*11:06
**594**	608	WREFCGLPRLETPAD	5.6	HLA-DRB1*11:11
**39**	53	VSSVLEESKRLVDTA	5.6	HLA-DRB1*11:11
**202**	216	LPPVREVTRHVIQVS	5.6	HLA-DRB1*11:37
**767**	781	SCRHGYELQGREQLT	5.7	HLA-DRB1*08:05
**494**	508	HATIHPLVRRLDASF	5.7	HLA-DRB1*08:07
**239**	253	HDIAFTPQSTSKAAF	5.7	HLA-DRB1*11:08
**532**	546	GGGLDPLIRGLLARP	5.7	HLA-DRB1*11:21
**430**	444	ADAVYQEARKVVGAL	5.8	HLA-DRB1*03:15
**413**	427	EHNRLAAALKALNAH	5.8	HLA-DRB1*08:05
**413**	427	EHNRLAAALKALNAH	5.8	HLA-DRB1*08:11
**418**	432	AAALKALNAHWSADA	5.8	HLA-DRB1*11:04
**425**	439	NAHWSADAVYQEARK	5.9	HLA-DRB1*03:01
**52**	66	TAMYATMQRNLKKRG	5.9	HLA-DRB1*03:05
**425**	439	NAHWSADAVYQEARK	5.9	HLA-DRB1*03:23
**425**	439	NAHWSADAVYQEARK	5.9	HLA-DRB1*03:36
**470**	484	YEGYDSTANPTVSNV	5.9	HLA-DRB1*04:05
**95**	109	ETSIQAMKRKVNLKT	5.9	HLA-DRB1*08:03
**360**	374	GRAYLPFVPPRAPSA	5.9	HLA-DRB1*08:04
**95**	109	ETSIQAMKRKVNLKT	5.9	HLA-DRB1*08:14
**360**	374	GRAYLPFVPPRAPSA	5.9	HLA-DRB1*08:31

**540**	554	RGLLARPAKLQVQDQ	5.9	HLA-DRB1*08:31
**346**	360	AEGLLRVHARLRDSG	5.9	HLA-DRB1*08:31
**239**	253	HDIAFTPQSTSKAAF	5.9	HLA-DRB1*11:01
**239**	253	HDIAFTPQSTSKAAF	5.9	HLA-DRB1*11:09
**239**	253	HDIAFTPQSTSKAAF	5.9	HLA-DRB1*11:10
**239**	253	HDIAFTPQSTSKAAF	5.9	HLA-DRB1*11:15
**239**	253	HDIAFTPQSTSKAAF	5.9	HLA-DRB1*11:29
**360**	374	GRAYLPFVPPRAPSA	5.9	HLA-DRB1*14:15
**767**	781	SCRHGYELQGREQLT	6.0	HLA-DRB1*08:11
**346**	360	AEGLLRVHARLRDSG	6.0	HLA-DRB1*11:01
**346**	360	AEGLLRVHARLRDSG	6.0	HLA-DRB1*11:09
**346**	360	AEGLLRVHARLRDSG	6.0	HLA-DRB1*11:10
**408**	422	TLWLREHNRLAAALK	6.0	HLA-DRB1*11:13
**346**	360	AEGLLRVHARLRDSG	6.0	HLA-DRB1*11:15
**346**	360	AEGLLRVHARLRDSG	6.0	HLA-DRB1*11:29
**767**	781	SCRHGYELQGREQLT	6.1	HLA-DRB1*03:05
**340**	354	LRNWTSAEGLLRVHA	6.1	HLA-DRB1*03:14
**209**	223	TRHVIQVSNEVVTDD	6.1	HLA-DRB1*04:05
**594**	608	WREFCGLPRLETPAD	6.1	HLA-DRB1*08:01
**102**	116	KRKVNLKTQQSQHPT	6.1	HLA-DRB1*08:03
**102**	116	KRKVNLKTQQSQHPT	6.1	HLA-DRB1*08:14
**594**	608	WREFCGLPRLETPAD	6.1	HLA-DRB1*08:16
**408**	422	TLWLREHNRLAAALK	6.1	HLA-DRB1*11:04
**408**	422	TLWLREHNRLAAALK	6.1	HLA-DRB1*11:06
**353**	367	HARLRDSGRAYLPFV	6.1	HLA-DRB1*11:13
**202**	216	LPPVREVTRHVIQVS	6.2	HLA-DRB1*08:01
**202**	216	LPPVREVTRHVIQVS	6.2	HLA-DRB1*08:16
**767**	781	SCRHGYELQGREQLT	6.2	HLA-DRB1*11:05
**418**	432	AAALKALNAHWSADA	6.2	HLA-DRB1*11:05
**761**	775	RRVLVYSCRHGYELQ	6.2	HLA-DRB1*11:11
**540**	554	RGLLARPAKLQVQDQ	6.2	HLA-DRB1*11:11
**413**	427	EHNRLAAALKALNAH	6.2	HLA-DRB1*11:13
**319**	333	GLTSFLDASTVYGSS	6.3	HLA-DRB1*04:05
**39**	53	VSSVLEESKRLVDTA	6.3	HLA-DRB1*11:01
**39**	53	VSSVLEESKRLVDTA	6.3	HLA-DRB1*11:09
**39**	53	VSSVLEESKRLVDTA	6.3	HLA-DRB1*11:10
**39**	53	VSSVLEESKRLVDTA	6.3	HLA-DRB1*11:15
**39**	53	VSSVLEESKRLVDTA	6.3	HLA-DRB1*11:29
**346**	360	AEGLLRVHARLRDSG	6.4	HLA-DRB1*03:15
**346**	360	AEGLLRVHARLRDSG	6.4	HLA-DRB1*11:05
**532**	546	GGGLDPLIRGLLARP	6.4	HLA-DRB1*11:11
**614**	628	ASRSVADKILDLYKH	6.4	HLA-DRB4*01:01
**39**	53	VSSVLEESKRLVDTA	6.5	HLA-DRB1*03:05
**102**	116	KRKVNLKTQQSQHPT	6.5	HLA-DRB1*08:05
**83**	97	TSGVIARAAEIMETS	6.5	HLA-DRB1*08:07
**239**	253	HDIAFTPQSTSKAAF	6.5	HLA-DRB1*08:07
**239**	253	HDIAFTPQSTSKAAF	6.5	HLA-DRB1*11:19
**52**	66	TAMYATMQRNLKKRG	6.5	HLA-DRB1*11:25
**425**	439	NAHWSADAVYQEARK	6.6	HLA-DRB1*03:15
**202**	216	LPPVREVTRHVIQVS	6.6	HLA-DRB1*08:03
**202**	216	LPPVREVTRHVIQVS	6.6	HLA-DRB1*08:14

**761**	775	RRVLVYSCRHGYELQ	6.6	HLA-DRB1*11:13
**418**	432	AAALKALNAHWSADA	6.6	HLA-DRB1*11:25
**413**	427	EHNRLAAALKALNAH	6.7	HLA-DRB1*08:12
**239**	253	HDIAFTPQSTSKAAF	6.7	HLA-DRB1*08:31
**102**	116	KRKVNLKTQQSQHPT	6.7	HLA-DRB1*11:01
**102**	116	KRKVNLKTQQSQHPT	6.7	HLA-DRB1*11:09
**102**	116	KRKVNLKTQQSQHPT	6.7	HLA-DRB1*11:10
**102**	116	KRKVNLKTQQSQHPT	6.7	HLA-DRB1*11:15
**102**	116	KRKVNLKTQQSQHPT	6.7	HLA-DRB1*11:29
**413**	427	EHNRLAAALKALNAH	6.8	HLA-DRB1*08:03
**540**	554	RGLLARPAKLQVQDQ	6.8	HLA-DRB1*08:06
**102**	116	KRKVNLKTQQSQHPT	6.8	HLA-DRB1*08:07
**532**	546	GGGLDPLIRGLLARP	6.8	HLA-DRB1*08:11
**413**	427	EHNRLAAALKALNAH	6.8	HLA-DRB1*08:14
**346**	360	AEGLLRVHARLRDSG	6.8	HLA-DRB1*11:37
**715**	729	PEDFESCDSITGMNL	6.9	HLA-DRB1*04:05
**346**	360	AEGLLRVHARLRDSG	7.0	HLA-DRB1*08:04
**52**	66	TAMYATMQRNLKKRG	7.0	HLA-DRB1*08:31
**346**	360	AEGLLRVHARLRDSG	7.0	HLA-DRB1*14:15
**52**	66	TAMYATMQRNLKKRG	7.1	HLA-DRB1*11:03
**767**	781	SCRHGYELQGREQLT	7.1	HLA-DRB1*11:11
**209**	223	TRHVIQVSNEVVTDD	7.1	HLA-DRB1*11:14
**209**	223	TRHVIQVSNEVVTDD	7.1	HLA-DRB1*11:20
**202**	216	LPPVREVTRHVIQVS	7.2	HLA-DRB1*08:02
**767**	781	SCRHGYELQGREQLT	7.2	HLA-DRB1*08:03
**202**	216	LPPVREVTRHVIQVS	7.2	HLA-DRB1*08:09
**767**	781	SCRHGYELQGREQLT	7.2	HLA-DRB1*08:14
**413**	427	EHNRLAAALKALNAH	7.2	HLA-DRB1*11:03
**102**	116	KRKVNLKTQQSQHPT	7.2	HLA-DRB1*11:37
**90**	104	AAEIMETSIQAMKRK	7.2	HLA-DRB4*01:01
**202**	216	LPPVREVTRHVIQVS	7.3	HLA-DRB1*03:15
**70**	84	PAQLLSFSKLPEPTS	7.3	HLA-DRB1*03:15
**767**	781	SCRHGYELQGREQLT	7.3	HLA-DRB1*08:01
**418**	432	AAALKALNAHWSADA	7.3	HLA-DRB1*08:02
**418**	432	AAALKALNAHWSADA	7.3	HLA-DRB1*08:09
**767**	781	SCRHGYELQGREQLT	7.3	HLA-DRB1*08:16
**430**	444	ADAVYQEARKVVGAL	7.4	HLA-DRB1*03:01
**761**	775	RRVLVYSCRHGYELQ	7.4	HLA-DRB1*03:07
**430**	444	ADAVYQEARKVVGAL	7.4	HLA-DRB1*03:23
**430**	444	ADAVYQEARKVVGAL	7.4	HLA-DRB1*03:36
**504**	518	LDASFQEHPDLPGLW	7.4	HLA-DRB1*04:05
**532**	546	GGGLDPLIRGLLARP	7.4	HLA-DRB1*08:07
**761**	775	RRVLVYSCRHGYELQ	7.4	HLA-DRB1*11:07
**413**	427	EHNRLAAALKALNAH	7.4	HLA-DRB1*11:11
**767**	781	SCRHGYELQGREQLT	7.4	HLA-DRB1*11:14
**767**	781	SCRHGYELQGREQLT	7.4	HLA-DRB1*11:20
**828**	842	PYELGDDGRTCVDSG	7.5	HLA-DRB1*03:01
**234**	248	GQYIDHDIAFTPQST	7.5	HLA-DRB1*03:01
**828**	842	PYELGDDGRTCVDSG	7.5	HLA-DRB1*03:23
**234**	248	GQYIDHDIAFTPQST	7.5	HLA-DRB1*03:23
**828**	842	PYELGDDGRTCVDSG	7.5	HLA-DRB1*03:36

**234**	248	GQYIDHDIAFTPQST	7.5	HLA-DRB1*03:36
**202**	216	LPPVREVTRHVIQVS	7.5	HLA-DRB1*08:05
**239**	253	HDIAFTPQSTSKAAF	7.5	HLA-DRB1*08:05
**430**	444	ADAVYQEARKVVGAL	7.6	HLA-DRB1*03:05
**234**	248	GQYIDHDIAFTPQST	7.6	HLA-DRB1*03:06
**761**	775	RRVLVYSCRHGYELQ	7.6	HLA-DRB1*03:15
**532**	546	GGGLDPLIRGLLARP	7.6	HLA-DRB1*08:05
**866**	880	TSTVICRWTRTGTKS	7.6	HLA-DRB1*11:03
**14**	28	ACTEAFFPFISRGKE	7.6	HLA-DRB1*11:14
**14**	28	ACTEAFFPFISRGKE	7.6	HLA-DRB1*11:20
**619**	633	ADKILDLYKHPDNID	7.6	HLA-DRB1*11:52
**14**	28	ACTEAFFPFISRGKE	7.7	HLA-DRB1*11:11
**430**	444	ADAVYQEARKVVGAL	7.8	HLA-DRB1*08:06
**540**	554	RGLLARPAKLQVQDQ	7.8	HLA-DRB1*11:06
**19**	33	FFPFISRGKELLWGK	7.8	HLA-DRB1*11:19
**828**	842	PYELGDDGRTCVDSG	7.9	HLA-DRB1*03:06
**767**	781	SCRHGYELQGREQLT	7.9	HLA-DRB1*08:07
**239**	253	HDIAFTPQSTSKAAF	7.9	HLA-DRB1*08:11
**102**	116	KRKVNLKTQQSQHPT	7.9	HLA-DRB1*11:13
**360**	374	GRAYLPFVPPRAPSA	7.9	HLA-DRB1*11:25
**102**	116	KRKVNLKTQQSQHPT	7.9	HLA-DRB1*11:27
**527**	541	WTLLRGGGLDPLIRG	7.9	HLA-DRB4*01:01
**319**	333	GLTSFLDASTVYGSS	8.0	HLA-DRB1*08:02
**83**	97	TSGVIARAAEIMETS	8.0	HLA-DRB1*08:06
**319**	333	GLTSFLDASTVYGSS	8.0	HLA-DRB1*08:09
**209**	223	TRHVIQVSNEVVTDD	8.0	HLA-DRB1*08:11
**418**	432	AAALKALNAHWSADA	8.0	HLA-DRB1*11:03
**360**	374	GRAYLPFVPPRAPSA	8.0	HLA-DRB1*11:06
**202**	216	LPPVREVTRHVIQVS	8.1	HLA-DRB1*03:07
**532**	546	GGGLDPLIRGLLARP	8.1	HLA-DRB1*08:02
**209**	223	TRHVIQVSNEVVTDD	8.1	HLA-DRB1*08:03
**532**	546	GGGLDPLIRGLLARP	8.1	HLA-DRB1*08:09
**346**	360	AEGLLRVHARLRDSG	8.1	HLA-DRB1*08:11
**209**	223	TRHVIQVSNEVVTDD	8.1	HLA-DRB1*08:14
**360**	374	GRAYLPFVPPRAPSA	8.1	HLA-DRB1*11:04
**202**	216	LPPVREVTRHVIQVS	8.1	HLA-DRB1*11:07
**52**	66	TAMYATMQRNLKKRG	8.1	HLA-DRB1*11:21
**143**	157	PNTCLANKYRPITGA	8.1	HLA-DRB1*11:25
**346**	360	AEGLLRVHARLRDSG	8.2	HLA-DRB1*03:01
**667**	681	GDWFWWENSHVFTDA	8.2	HLA-DRB1*03:14
**346**	360	AEGLLRVHARLRDSG	8.2	HLA-DRB1*03:23
**346**	360	AEGLLRVHARLRDSG	8.2	HLA-DRB1*03:36
**39**	53	VSSVLEESKRLVDTA	8.2	HLA-DRB1*11:05
**667**	681	GDWFWWENSHVFTDA	8.3	HLA-DRB1*03:40
**594**	608	WREFCGLPRLETPAD	8.3	HLA-DRB1*08:11
**151**	165	YRPITGACNNRDHPR	8.4	HLA-DRB1*08:05
**95**	109	ETSIQAMKRKVNLKT	8.4	HLA-DRB1*08:07
**733**	747	RETFPQDDKCGFPES	8.5	HLA-DRB1*03:14
**570**	584	SSTLDLASINLQRGR	8.5	HLA-DRB1*08:31
**540**	554	RGLLARPAKLQVQDQ	8.5	HLA-DRB1*11:05
**761**	775	RRVLVYSCRHGYELQ	8.6	HLA-DRB1*03:01

**494**	508	HATIHPLVRRLDASF	8.6	HLA-DRB1*03:15
**761**	775	RRVLVYSCRHGYELQ	8.6	HLA-DRB1*03:23
**761**	775	RRVLVYSCRHGYELQ	8.6	HLA-DRB1*03:36
**418**	432	AAALKALNAHWSADA	8.6	HLA-DRB1*11:01
**540**	554	RGLLARPAKLQVQDQ	8.6	HLA-DRB1*11:04
**418**	432	AAALKALNAHWSADA	8.6	HLA-DRB1*11:09
**418**	432	AAALKALNAHWSADA	8.6	HLA-DRB1*11:10
**239**	253	HDIAFTPQSTSKAAF	8.6	HLA-DRB1*11:11
**418**	432	AAALKALNAHWSADA	8.6	HLA-DRB1*11:15
**418**	432	AAALKALNAHWSADA	8.6	HLA-DRB1*11:29
**209**	223	TRHVIQVSNEVVTDD	8.6	HLA-DRB4*01:01
**733**	747	RETFPQDDKCGFPES	8.7	HLA-DRB1*03:05
**353**	367	HARLRDSGRAYLPFV	8.7	HLA-DRB1*03:07
**532**	546	GGGLDPLIRGLLARP	8.7	HLA-DRB1*08:01
**532**	546	GGGLDPLIRGLLARP	8.7	HLA-DRB1*08:16
**353**	367	HARLRDSGRAYLPFV	8.7	HLA-DRB1*11:07
**437**	451	ARKVVGALHQIITLR	8.7	HLA-DRB1*11:13
**430**	444	ADAVYQEARKVVGAL	8.8	HLA-DRB1*03:06
**27**	41	KELLWGKPEESRVSS	8.8	HLA-DRB1*11:02
**27**	41	KELLWGKPEESRVSS	8.8	HLA-DRB1*11:16
**654**	668	ACLIGKQMKALRDGD	8.9	HLA-DRB1*03:07
**828**	842	PYELGDDGRTCVDSG	8.9	HLA-DRB1*03:15
**654**	668	ACLIGKQMKALRDGD	8.9	HLA-DRB1*03:15
**918**	932	SAGMEGRDTHRLPRA	8.9	HLA-DRB1*11:02
**333**	347	SPALERQLRNWTSAE	8.9	HLA-DRB1*11:03
**654**	668	ACLIGKQMKALRDGD	8.9	HLA-DRB1*11:03
**654**	668	ACLIGKQMKALRDGD	8.9	HLA-DRB1*11:07
**619**	633	ADKILDLYKHPDNID	8.9	HLA-DRB1*11:14
**918**	932	SAGMEGRDTHRLPRA	8.9	HLA-DRB1*11:16
**619**	633	ADKILDLYKHPDNID	8.9	HLA-DRB1*11:20
**418**	432	AAALKALNAHWSADA	8.9	HLA-DRB1*11:37
**346**	360	AEGLLRVHARLRDSG	9.0	HLA-DRB1*03:06
**151**	165	YRPITGACNNRDHPR	9.0	HLA-DRB1*08:01
**314**	328	RQQMNGLTSFLDAST	9.0	HLA-DRB1*08:12
**151**	165	YRPITGACNNRDHPR	9.0	HLA-DRB1*08:16
**143**	157	PNTCLANKYRPITGA	9.0	HLA-DRB1*11:02
**333**	347	SPALERQLRNWTSAE	9.0	HLA-DRB1*11:04
**333**	347	SPALERQLRNWTSAE	9.0	HLA-DRB1*11:06
**641**	655	ENFLPRARTGPLFAC	9.0	HLA-DRB1*11:11
**143**	157	PNTCLANKYRPITGA	9.0	HLA-DRB1*11:16
**408**	422	TLWLREHNRLAAALK	9.0	HLA-DRB1*11:27
**667**	681	GDWFWWENSHVFTDA	9.1	HLA-DRB1*03:05
**918**	932	SAGMEGRDTHRLPRA	9.1	HLA-DRB1*08:04
**314**	328	RQQMNGLTSFLDAST	9.1	HLA-DRB1*08:05
**151**	165	YRPITGACNNRDHPR	9.1	HLA-DRB1*08:06
**314**	328	RQQMNGLTSFLDAST	9.1	HLA-DRB1*08:10
**19**	33	FFPFISRGKELLWGK	9.1	HLA-DRB1*11:08
**918**	932	SAGMEGRDTHRLPRA	9.1	HLA-DRB1*11:21
**918**	932	SAGMEGRDTHRLPRA	9.1	HLA-DRB1*14:15
**751**	765	GDFVHCEESGRRVLV	9.2	HLA-DRB1*03:01
**751**	765	GDFVHCEESGRRVLV	9.2	HLA-DRB1*03:23

**751**	765	GDFVHCEESGRRVLV	9.2	HLA-DRB1*03:36
**703**	717	RVPMDAFQVGKFPED	9.2	HLA-DRB1*08:12
**52**	66	TAMYATMQRNLKKRG	9.2	HLA-DRB1*11:02
**14**	28	ACTEAFFPFISRGKE	9.2	HLA-DRB1*11:05
**327**	341	STVYGSSPALERQLR	9.2	HLA-DRB1*11:14
**52**	66	TAMYATMQRNLKKRG	9.2	HLA-DRB1*11:16
**327**	341	STVYGSSPALERQLR	9.2	HLA-DRB1*11:20
**761**	775	RRVLVYSCRHGYELQ	9.2	HLA-DRB1*11:52
**918**	932	SAGMEGRDTHRLPRA	9.3	HLA-DRB1*08:11
**418**	432	AAALKALNAHWSADA	9.3	HLA-DRB1*08:12
**102**	116	KRKVNLKTQQSQHPT	9.3	HLA-DRB1*11:08
**333**	347	SPALERQLRNWTSAE	9.3	HLA-DRB1*11:13
**408**	422	TLWLREHNRLAAALK	9.3	HLA-DRB1*11:19
**83**	97	TSGVIARAAEIMETS	9.4	HLA-DRB1*08:04
**239**	253	HDIAFTPQSTSKAAF	9.4	HLA-DRB1*08:04
**918**	932	SAGMEGRDTHRLPRA	9.4	HLA-DRB1*11:03
**83**	97	TSGVIARAAEIMETS	9.4	HLA-DRB1*14:15
**239**	253	HDIAFTPQSTSKAAF	9.4	HLA-DRB1*14:15
**239**	253	HDIAFTPQSTSKAAF	9.5	HLA-DRB1*08:01
**540**	554	RGLLARPAKLQVQDQ	9.5	HLA-DRB1*08:12
**239**	253	HDIAFTPQSTSKAAF	9.5	HLA-DRB1*08:16
**408**	422	TLWLREHNRLAAALK	9.5	HLA-DRB1*11:01
**408**	422	TLWLREHNRLAAALK	9.5	HLA-DRB1*11:09
**408**	422	TLWLREHNRLAAALK	9.5	HLA-DRB1*11:10
**408**	422	TLWLREHNRLAAALK	9.5	HLA-DRB1*11:15
**408**	422	TLWLREHNRLAAALK	9.5	HLA-DRB1*11:29
**90**	104	AAEIMETSIQAMKRK	9.5	HLA-DRB1*11:52
**209**	223	TRHVIQVSNEVVTDD	9.6	HLA-DRB1*08:06
**703**	717	RVPMDAFQVGKFPED	9.6	HLA-DRB1*08:10
**674**	688	NSHVFTDAQRRELEK	9.6	HLA-DRB1*11:03
**400**	414	VPSLTALHTLWLREH	9.6	HLA-DRB1*11:13
**891**	905	TPELRCGKHQAVGTS	9.6	HLA-DRB1*11:52
**606**	620	PADLSTAIASRSVAD	9.6	HLA-DRB4*01:01
**751**	765	GDFVHCEESGRRVLV	9.7	HLA-DRB1*03:07
**83**	97	TSGVIARAAEIMETS	9.7	HLA-DRB1*08:11
**540**	554	RGLLARPAKLQVQDQ	9.7	HLA-DRB1*08:11
**594**	608	WREFCGLPRLETPAD	9.7	HLA-DRB1*11:06
**751**	765	GDFVHCEESGRRVLV	9.7	HLA-DRB1*11:07
**408**	422	TLWLREHNRLAAALK	9.7	HLA-DRB1*11:08
**418**	432	AAALKALNAHWSADA	9.7	HLA-DRB1*11:27
**425**	439	NAHWSADAVYQEARK	9.8	HLA-DRB1*03:07
**828**	842	PYELGDDGRTCVDSG	9.8	HLA-DRB1*03:14
**353**	367	HARLRDSGRAYLPFV	9.8	HLA-DRB1*03:15
**918**	932	SAGMEGRDTHRLPRA	9.8	HLA-DRB1*08:06
**425**	439	NAHWSADAVYQEARK	9.8	HLA-DRB1*11:07
**327**	341	STVYGSSPALERQLR	9.8	HLA-DRB1*11:08
**52**	66	TAMYATMQRNLKKRG	9.8	HLA-DRB1*11:13
**532**	546	GGGLDPLIRGLLARP	9.8	HLA-DRB1*11:14
**532**	546	GGGLDPLIRGLLARP	9.8	HLA-DRB1*11:20
**751**	765	GDFVHCEESGRRVLV	9.9	HLA-DRB1*03:15
**102**	116	KRKVNLKTQQSQHPT	9.9	HLA-DRB1*11:14

**102**	116	KRKVNLKTQQSQHPT	9.9	HLA-DRB1*11:19
**102**	116	KRKVNLKTQQSQHPT	9.9	HLA-DRB1*11:20
**27**	41	KELLWGKPEESRVSS	9.9	HLA-DRB1*11:21
**346**	360	AEGLLRVHARLRDSG	10.0	HLA-DRB1*03:40
**360**	374	GRAYLPFVPPRAPSA	10.0	HLA-DRB1*08:06
**52**	66	TAMYATMQRNLKKRG	10.0	HLA-DRB1*08:07
**360**	374	GRAYLPFVPPRAPSA	10.0	HLA-DRB1*08:10
**239**	253	HDIAFTPQSTSKAAF	10.0	HLA-DRB1*11:52
