# Supplementary material for: Predicted T-Cell and B-Cell Epitopes of NIS: Where Do Sjögren’s Syndrome and Hashimoto’s Thyroiditis Converge?
Source: Int J Mol Sci. 2025 Dec 24;27(1):200. doi: 10.3390/ijms27010200 (PMC12785876; doi:10.3390/ijms27010200)
Supplement: Supplementary file 1 [file ijms-27-00200-s001.zip › Table S3 IJMS.pdf]

Rank	Sequence	Start position	Score
**1**	QQYVGPYEGYDSTANP	464	0.95
**1**	RDSGRAYLPFVPPRAP	357	0.95
**2**	PCHASARCRNTKGGFQ	807	0.92
**2**	CGTGDQGALFGNLSTA	296	0.92
**2**	CFPIQLPEEARPAAGT	269	0.92
**2**	GQYIDHDIAFTPQSTS	234	0.92
**2**	HPRWGASNTALARWLP	163	0.92
**3**	QEGWDFQPPLCKDVNE	784	0.91
**3**	AFFSPWTLLRGGGLDP	522	0.91
**3**	SACAPEPGIPGETRGP	373	0.91
**3**	YRSSAACGTGDQGALF	290	0.91
**3**	TGACNNRDHPRWGASN	155	0.91
**4**	RRELEKHSLSRVICDN	683	0.90
**4**	GADCQMTCENQNPCFP	256	0.90
**5**	ETSIQAMKRKVNLKTQ	95	0.89
**6**	TVICRWTRTGTKSTLP	868	0.88
**6**	RGPCFLAGDGRATEVP	386	0.88
**6**	PGIPGETRGPCFLAGD	379	0.88
**6**	QSTSKAAFGGGADCQM	246	0.88
**7**	PISETGGGTPELRCGK	883	0.87
**7**	CLCADPYELGDDGRTC	823	0.87
**7**	DNTGLTRVPMDAFQVG	697	0.87
**7**	HGLPGYNEWREFCGLP	586	0.87
**7**	AVYQEARKVVGALHQI	432	0.87
**8**	STAIASRSVADKILDL	610	0.86
**9**	LEAWRETFPQDDKCGF	729	0.85
**9**	FESCDSITGMNLEAWR	718	0.85
**9**	ASFQEHPDLPGLWLHQ	506	0.85
**9**	IPRILGPEAFQQYVGP	454	0.85
**9**	RVHARLRDSGRAYLPF	351	0.85

**10**	TFPQDDKCGFPESVEN	735	0.84
**11**	QESAGMEGRDTHRLPR	916	0.83
**11**	GTKSTLPISETGGGTP	877	0.83
**11**	DKILDLYKHPDNIDVW	620	0.83
**11**	AKLQVQDQLMNEELTE	547	0.83
**11**	LARWLPPVYEDGFSQP	173	0.83
**12**	YATMQRNLKKRGILSP	55	0.82
**12**	ANMSGCLPYMLPPKCP	128	0.82
**13**	HQAVGTSPQRAAAQDS	899	0.81
**13**	FQVGKFPEDFESCDSI	709	0.81
**13**	RGGGLDPLIRGLLARP	531	0.81
**13**	ELLWGKPEESRVSSVL	28	0.81
**13**	DDDRYSDLLMAWGQYI	222	0.81
**13**	DGFSQPRGWNPGFLYN	183	0.81
**14**	PRQQMNGLTSFLDAST	313	0.80
**15**	RTCVDSGRLPRVTWIS	836	0.79
**15**	LASINLQRGRDHGLPG	575	0.79
**15**	AGDGRATEVPSLTALH	392	0.79
**15**	RGWNPGFLYNGFPLPP	189	0.79
**16**	GKQMKALRDGDWFWWE	658	0.78
**16**	PRARTGPLFACLIGKQ	645	0.78
**16**	TVYGSSPALERQLRNW	328	0.78
**16**	RHVIQVSNEVVTDDDR	210	0.78
**17**	PPLCKDVNECADGAHP	791	0.77
**17**	EFCGLPRLETPADLST	596	0.77
**18**	CRHGYELQGREQLTCT	768	0.76
**18**	GHATIHPLVRRLDASF	493	0.76
**18**	PPKCPNTCLANKYRPI	139	0.76
**19**	AGTACLPFYRSSAACG	282	0.75
**19**	TCLANKYRPITGACNN	145	0.75
**20**	LIGGFAGLTSTVICRW	858	0.74

**20**	ELGDDGRTCVDSGRLP	830	0.74
**20**	LVDTAMYATMQRNLKK	49	0.74
**20**	VMACTEAFFPFISRGK	12	0.74
**21**	NLSTANPRQQMNGLTS	307	0.73
**22**	DGDWFWWENSHVFTDA	666	0.72
**22**	ESRVSSVLEESKRLVD	36	0.72
**23**	GVIARAAEIMETSIQA	85	0.71
**23**	CGFPESVENGDFVHCE	742	0.71
**24**	HQIITLRDYIPRILGP	445	0.70
**25**	RCRNTKGGFQCLCADP	813	0.69
**25**	DFVHCEESGRRVLVYS	752	0.69
**25**	LGGLAENFLPRARTGP	636	0.69
**25**	TSFLDASTVYGSSPAL	321	0.69
**25**	FLYNGFPLPPVREVTR	195	0.69
**26**	YEGYDSTANPTVSNVF	470	0.68
**26**	AAALKALNAHWSADAV	418	0.68
**26**	PLPPVREVTRHVIQVS	201	0.68
**26**	SQHPTDALSEDLLSII	112	0.68
**27**	MSLAALLIGGFAGLTS	852	0.66
**27**	LPEPTSGVIARAAEIM	79	0.66
**27**	TCENQNPCFPIQLPEE	262	0.66
**28**	PGLWLHQAFFSPWTLL	515	0.65
**28**	ALERQLRNWTSAEGLL	335	0.65
**29**	HVFTDAQRRELEKHSL	676	0.64
**29**	VSNVFSTAAFRFGHAT	481	0.64
**30**	GRLPRVTWISMSLAAL	842	0.62
**31**	YKHPDNIDVWLGGLAE	626	0.61
**32**	TALHTLWLREHNRLAA	404	0.60
**32**	NWTSAEGLLRVHARLR	342	0.60
**33**	MNEELTERLFVLSNSS	556	0.59
**34**	GILSPAQLLSFSKLPE	66	0.53

**35**	LVRRLDASFQEHPDLP	500	0.51
