# Supplementary material for: Predicted T-Cell and B-Cell Epitopes of NIS: Where Do Sjögren’s Syndrome and Hashimoto’s Thyroiditis Converge?
Source: Int J Mol Sci. 2025 Dec 24;27(1):200. doi: 10.3390/ijms27010200 (PMC12785876; doi:10.3390/ijms27010200)
Supplement: Supplementary file 1 [file ijms-27-00200-s001.zip › Table S4 IJMS.pdf]

Chain ID	Residue ID	Residue name	Contact number	Propensity score	DiscoTope score
**A**	195	PHE	15	5.854	3.456
**A**	196	LEU	4	4.061	3.134
**A**	114	HIS	11	4.484	2.704
**A**	925	ASP	0	2.992	2.648
**A**	111	GLN	2	3.195	2.597
**A**	112	SER	2	2.945	2.376
**A**	199	GLY	7	3.392	2.197
**A**	926	THR	2	2.722	2.179
**A**	110	GLN	0	2.334	2.065
**A**	927	HIS	1	2.409	2.017
**A**	184	GLY	10	3.501	1.948
**A**	924	ARG	4	2.561	1.806
**A**	921	MET	0	1.956	1.731
**A**	194	GLY	0	1.747	1.546
**A**	928	ARG	1	1.779	1.459
**A**	923	GLY	3	1.998	1.423
**A**	922	GLU	4	1.985	1.297
**A**	113	GLN	15	2.986	0.917
**A**	918	SER	1	1.107	0.864
**A**	917	GLU	0	0.934	0.826
**A**	920	GLY	4	1.337	0.723
**A**	192	ASN	10	2.057	0.67
**A**	929	LEU	2	0.865	0.536
**A**	916	GLN	1	0.731	0.532
**A**	919	ALA	4	0.957	0.387
**A**	912	GLN	0	0.307	0.272
**A**	904	THR	0	0.18	0.159
**A**	887	THR	1	0.303	0.153
**A**	197	TYR	33	4.458	0.151
**A**	889	GLY	3	0.547	0.139
**A**	930	PRO	2	0.406	0.13

**A**	881	THR	0	0.118	0.104
**A**	913	ASP	0	0.009	0.008
**A**	914	SER	0	-0.008	-0.007
**A**	471	GLU	12	1.54	-0.017
**A**	915	GLU	1	0.084	-0.041
**A**	903	GLY	4	0.449	-0.063
**A**	888	GLY	2	0.171	-0.078
**A**	200	PHE	16	1.99	-0.079
**A**	886	GLU	1	0.026	-0.092
**A**	183	ASP	22	2.641	-0.192
**A**	885	SER	0	-0.275	-0.243
**A**	931	ARG	0	-0.33	-0.292
**A**	893	GLU	1	-0.203	-0.295
**A**	908	ARG	2	-0.143	-0.356
**A**	902	VAL	0	-0.431	-0.381
**A**	892	PRO	3	-0.125	-0.456
**A**	890	GLY	2	-0.256	-0.457
**A**	907	GLN	3	-0.187	-0.511
**A**	910	ALA	0	-0.588	-0.521
**A**	905	SER	2	-0.341	-0.531
**A**	906	PRO	3	-0.21	-0.531
**A**	891	THR	2	-0.405	-0.589
**A**	107	LEU	6	0.097	-0.604
**A**	884	ILE	0	-0.691	-0.612
**A**	911	ALA	4	-0.224	-0.658
**A**	901	ALA	1	-0.624	-0.667
**A**	909	ALA	3	-0.474	-0.765
**A**	900	GLN	0	-0.904	-0.8
**A**	932	ALA	0	-0.939	-0.831
**A**	115	PRO	16	1.131	-0.839
**A**	883	PRO	4	-0.444	-0.853
**A**	933	LEU	0	-0.995	-0.881

**A**	182	GLU	22	1.841	-0.901
**A**	472	GLY	3	-0.63	-0.902
**A**	106	ASN	14	0.745	-0.951
**A**	880	SER	5	-0.475	-0.995
**A**	894	LEU	0	-1.211	-1.072
**A**	109	THR	14	0.471	-1.193
**A**	882	LEU	7	-0.442	-1.196
**A**	108	LYS	7	-0.561	-1.301
**A**	898	LYS	3	-1.183	-1.392
**A**	739	ASP	0	-1.574	-1.393
**A**	879	LYS	1	-1.467	-1.414
**A**	201	PRO	10	-0.358	-1.467
**A**	897	GLY	1	-1.529	-1.468
**A**	899	HIS	2	-1.635	-1.677
**A**	193	PRO	12	-0.353	-1.692
**A**	895	ARG	3	-1.579	-1.742
**A**	198	ASN	21	0.646	-1.843
**A**	896	CYS	0	-2.512	-2.223
**A**	383	GLY	1	-2.459	-2.291
**A**	476	THR	1	-2.536	-2.36
**A**	878	THR	4	-2.27	-2.469
**A**	627	LYS	9	-1.758	-2.59
**A**	382	PRO	0	-3.223	-2.853
**A**	784	GLN	0	-3.267	-2.892
**A**	181	TYR	25	-0.044	-2.914
**A**	738	GLN	0	-3.37	-2.983
**A**	30	LEU	7	-2.706	-3.199
**A**	680	ASP	0	-3.618	-3.202
**A**	31	TRP	11	-2.246	-3.253
**A**	475	SER	8	-2.709	-3.318
**A**	117	ASP	5	-3.16	-3.371
**A**	469	PRO	2	-3.568	-3.387

**A**	33	LYS	8	-2.82	-3.416
**A**	32	GLY	6	-3.184	-3.508
**A**	105	VAL	20	-1.444	-3.578
**A**	512	PRO	2	-3.824	-3.614
**A**	785	GLU	4	-3.566	-3.616
**A**	103	ARG	12	-2.601	-3.682
