# Supplementary material for: Predicted T-Cell and B-Cell Epitopes of NIS: Where Do Sjögren’s Syndrome and Hashimoto’s Thyroiditis Converge?
Source: Int J Mol Sci. 2025 Dec 24;27(1):200. doi: 10.3390/ijms27010200 (PMC12785876; doi:10.3390/ijms27010200)
Supplement: Supplementary file 1 [file ijms-27-00200-s001.zip › Table S5 IJMS REV.pdf]

Peptide Start	*Peptide End*	*Peptide Sequence*	*Percentile rank*	*Allele*
**354**	368	DAAFYKTFKTVEPTG	0.19	HLA-DRB1*08:05
**340**	354	KLKWRPDEEILKALD	0.23	HLA-DRB1*03:14
**275**	289	EMPLTALLRNLGKMT	0.24	HLA-DRB1*11:06
**275**	289	EMPLTALLRNLGKMT	0.25	HLA-DRB1*11:04
**226**	240	LKYLEAVEKVKRTRD	0.25	HLA-DRB1*11:13
**340**	354	KLKWRPDEEILKALD	0.26	HLA-DRB1*03:05
**354**	368	DAAFYKTFKTVEPTG	0.26	HLA-DRB1*08:01
**354**	368	DAAFYKTFKTVEPTG	0.26	HLA-DRB1*08:16
**275**	289	EMPLTALLRNLGKMT	0.27	HLA-DRB1*08:31
**226**	240	LKYLEAVEKVKRTRD	0.3	HLA-DRB1*11:02
**226**	240	LKYLEAVEKVKRTRD	0.3	HLA-DRB1*11:16
**354**	368	DAAFYKTFKTVEPTG	0.32	HLA-DRB1*08:03
**354**	368	DAAFYKTFKTVEPTG	0.32	HLA-DRB1*08:14
**340**	354	KLKWRPDEEILKALD	0.33	HLA-DRB1*03:40
**275**	289	EMPLTALLRNLGKMT	0.35	HLA-DRB1*11:25
**226**	240	LKYLEAVEKVKRTRD	0.36	HLA-DRB1*11:21
**275**	289	EMPLTALLRNLGKMT	0.42	HLA-DRB1*08:04
**275**	289	EMPLTALLRNLGKMT	0.42	HLA-DRB1*14:15
**126**	140	FTFIQFKKDLKESMK	0.45	HLA-DRB1*03:05
**126**	140	FTFIQFKKDLKESMK	0.45	HLA-DRB1*03:40
**191**	205	SSEGLAIVTKYITKG	0.46	HLA-DRB1*11:13
**126**	140	FTFIQFKKDLKESMK	0.48	HLA-DRB1*03:14
**275**	289	EMPLTALLRNLGKMT	0.5	HLA-DRB1*11:27
**191**	205	SSEGLAIVTKYITKG	0.51	HLA-DRB1*11:25
**191**	205	SSEGLAIVTKYITKG	0.52	HLA-DRB1*11:04
**275**	289	EMPLTALLRNLGKMT	0.54	HLA-DRB1*11:37
**275**	289	EMPLTALLRNLGKMT	0.56	HLA-DRB1*11:01
**275**	289	EMPLTALLRNLGKMT	0.56	HLA-DRB1*11:09
**275**	289	EMPLTALLRNLGKMT	0.56	HLA-DRB1*11:10
**275**	289	EMPLTALLRNLGKMT	0.56	HLA-DRB1*11:15
**275**	289	EMPLTALLRNLGKMT	0.56	HLA-DRB1*11:29
**44**	58	GTYYIKEQKLGLENA	0.59	HLA-DRB1*11:27
**74**	88	IQEIKSFSQEGRTTK	0.6	HLA-DRB1*11:02
**275**	289	EMPLTALLRNLGKMT	0.6	HLA-DRB1*11:05
**74**	88	IQEIKSFSQEGRTTK	0.6	HLA-DRB1*11:16
**44**	58	GTYYIKEQKLGLENA	0.62	HLA-DRB1*08:02
**44**	58	GTYYIKEQKLGLENA	0.62	HLA-DRB1*08:07
**44**	58	GTYYIKEQKLGLENA	0.62	HLA-DRB1*08:09
**191**	205	SSEGLAIVTKYITKG	0.62	HLA-DRB1*11:52
**44**	58	GTYYIKEQKLGLENA	0.63	HLA-DRB1*11:05
**191**	205	SSEGLAIVTKYITKG	0.64	HLA-DRB1*08:04
**126**	140	FTFIQFKKDLKESMK	0.64	HLA-DRB1*08:10
**44**	58	GTYYIKEQKLGLENA	0.64	HLA-DRB1*11:08
**191**	205	SSEGLAIVTKYITKG	0.64	HLA-DRB1*14:15
**44**	58	GTYYIKEQKLGLENA	0.65	HLA-DRB1*11:19
**44**	58	GTYYIKEQKLGLENA	0.66	HLA-DRB1*08:11
**243**	257	EVIHLIEEHRLVREH	0.67	HLA-DRB1*03:07
**243**	257	EVIHLIEEHRLVREH	0.67	HLA-DRB1*11:07
**354**	368	DAAFYKTFKTVEPTG	0.71	HLA-DRB1*08:11

**226**	240	LKYLEAVEKVKRTRD	0.72	HLA-DRB1*03:07
**226**	240	LKYLEAVEKVKRTRD	0.72	HLA-DRB1*11:07
**74**	88	IQEIKSFSQEGRTTK	0.72	HLA-DRB1*11:21
**126**	140	FTFIQFKKDLKESMK	0.75	HLA-DRB1*08:06
**226**	240	LKYLEAVEKVKRTRD	0.78	HLA-DRB1*11:14
**226**	240	LKYLEAVEKVKRTRD	0.78	HLA-DRB1*11:20
**191**	205	SSEGLAIVTKYITKG	0.8	HLA-DRB1*08:31
**275**	289	EMPLTALLRNLGKMT	0.8	HLA-DRB1*11:13
**275**	289	EMPLTALLRNLGKMT	0.81	HLA-DRB1*11:08
**44**	58	GTYYIKEQKLGLENA	0.81	HLA-DRB1*11:37
**126**	140	FTFIQFKKDLKESMK	0.84	HLA-DRB1*08:12
**191**	205	SSEGLAIVTKYITKG	0.84	HLA-DRB1*11:06
**191**	205	SSEGLAIVTKYITKG	0.86	HLA-DRB1*08:10
**474**	488	AGGVHPAIALREYRK	0.86	HLA-DRB1*08:12
**275**	289	EMPLTALLRNLGKMT	0.86	HLA-DRB1*11:19
**425**	439	TTDMTLQQVLMAMSQ	0.88	HLA-DRB4*01:01
**44**	58	GTYYIKEQKLGLENA	0.91	HLA-DRB1*11:01
**44**	58	GTYYIKEQKLGLENA	0.91	HLA-DRB1*11:09
**44**	58	GTYYIKEQKLGLENA	0.91	HLA-DRB1*11:10
**44**	58	GTYYIKEQKLGLENA	0.91	HLA-DRB1*11:15
**44**	58	GTYYIKEQKLGLENA	0.91	HLA-DRB1*11:29
**226**	240	LKYLEAVEKVKRTRD	0.92	HLA-DRB1*03:15
**191**	205	SSEGLAIVTKYITKG	0.93	HLA-DRB1*08:12
**44**	58	GTYYIKEQKLGLENA	0.96	HLA-DRB1*03:40
**191**	205	SSEGLAIVTKYITKG	0.97	HLA-DRB1*11:01
**191**	205	SSEGLAIVTKYITKG	0.97	HLA-DRB1*11:09
**191**	205	SSEGLAIVTKYITKG	0.97	HLA-DRB1*11:10
**191**	205	SSEGLAIVTKYITKG	0.97	HLA-DRB1*11:15
**191**	205	SSEGLAIVTKYITKG	0.97	HLA-DRB1*11:29
**275**	289	EMPLTALLRNLGKMT	1.1	HLA-DRB1*08:02
**275**	289	EMPLTALLRNLGKMT	1.1	HLA-DRB1*08:09
**474**	488	AGGVHPAIALREYRK	1.1	HLA-DRB1*08:10
**44**	58	GTYYIKEQKLGLENA	1.1	HLA-DRB1*11:11
**44**	58	GTYYIKEQKLGLENA	1.2	HLA-DRB1*03:14
**243**	257	EVIHLIEEHRLVREH	1.2	HLA-DRB1*03:15
**243**	257	EVIHLIEEHRLVREH	1.2	HLA-DRB1*11:02
**191**	205	SSEGLAIVTKYITKG	1.2	HLA-DRB1*11:05
**243**	257	EVIHLIEEHRLVREH	1.2	HLA-DRB1*11:16
**226**	240	LKYLEAVEKVKRTRD	1.2	HLA-DRB1*11:52
**316**	330	KARIHPFHILIALET	1.3	HLA-DRB1*08:12
**226**	240	LKYLEAVEKVKRTRD	1.3	HLA-DRB1*11:03
**226**	240	LKYLEAVEKVKRTRD	1.3	HLA-DRB1*11:08
**226**	240	LKYLEAVEKVKRTRD	1.3	HLA-DRB1*11:19
**191**	205	SSEGLAIVTKYITKG	1.3	HLA-DRB1*11:19
**243**	257	EVIHLIEEHRLVREH	1.3	HLA-DRB1*11:21
**191**	205	SSEGLAIVTKYITKG	1.3	HLA-DRB1*11:27
**243**	257	EVIHLIEEHRLVREH	1.4	HLA-DRB1*03:40
**44**	58	GTYYIKEQKLGLENA	1.4	HLA-DRB1*08:01
**44**	58	GTYYIKEQKLGLENA	1.4	HLA-DRB1*08:03
**44**	58	GTYYIKEQKLGLENA	1.4	HLA-DRB1*08:05
**191**	205	SSEGLAIVTKYITKG	1.4	HLA-DRB1*08:11

**44**	58	GTYYIKEQKLGLENA	1.4	HLA-DRB1*08:14
**44**	58	GTYYIKEQKLGLENA	1.4	HLA-DRB1*08:16
**334**	348	GHGLRGKLKWRPDEE	1.4	HLA-DRB1*11:03
**191**	205	SSEGLAIVTKYITKG	1.4	HLA-DRB1*11:08
**191**	205	SSEGLAIVTKYITKG	1.4	HLA-DRB1*11:37
**243**	257	EVIHLIEEHRLVREH	1.5	HLA-DRB1*03:01
**243**	257	EVIHLIEEHRLVREH	1.5	HLA-DRB1*03:23
**243**	257	EVIHLIEEHRLVREH	1.5	HLA-DRB1*03:36
**191**	205	SSEGLAIVTKYITKG	1.5	HLA-DRB1*08:06
**44**	58	GTYYIKEQKLGLENA	1.6	HLA-DRB1*03:05
**191**	205	SSEGLAIVTKYITKG	1.6	HLA-DRB1*08:02
**191**	205	SSEGLAIVTKYITKG	1.6	HLA-DRB1*08:09
**316**	330	KARIHPFHILIALET	1.6	HLA-DRB1*08:10
**448**	462	SLPMIWAQKTNTPAD	1.6	HLA-DRB4*01:01
**370**	384	RFLLAVDVSASMNQR	1.7	HLA-DRB1*03:01
**370**	384	RFLLAVDVSASMNQR	1.7	HLA-DRB1*03:23
**370**	384	RFLLAVDVSASMNQR	1.7	HLA-DRB1*03:36
**209**	223	VHELYKEKALSVETE	1.7	HLA-DRB1*08:01
**126**	140	FTFIQFKKDLKESMK	1.7	HLA-DRB1*08:01
**260**	274	TNHLKSKEVWKALLQ	1.7	HLA-DRB1*08:04
**474**	488	AGGVHPAIALREYRK	1.7	HLA-DRB1*08:06
**209**	223	VHELYKEKALSVETE	1.7	HLA-DRB1*08:16
**126**	140	FTFIQFKKDLKESMK	1.7	HLA-DRB1*08:16
**243**	257	EVIHLIEEHRLVREH	1.7	HLA-DRB1*11:13
**260**	274	TNHLKSKEVWKALLQ	1.7	HLA-DRB1*14:15
**370**	384	RFLLAVDVSASMNQR	1.8	HLA-DRB1*03:06
**126**	140	FTFIQFKKDLKESMK	1.8	HLA-DRB1*08:03
**126**	140	FTFIQFKKDLKESMK	1.8	HLA-DRB1*08:14
**191**	205	SSEGLAIVTKYITKG	1.8	HLA-DRB1*11:02
**44**	58	GTYYIKEQKLGLENA	1.8	HLA-DRB1*11:14
**191**	205	SSEGLAIVTKYITKG	1.8	HLA-DRB1*11:16
**44**	58	GTYYIKEQKLGLENA	1.8	HLA-DRB1*11:20
**275**	289	EMPLTALLRNLGKMT	1.8	HLA-DRB1*11:52
**226**	240	LKYLEAVEKVKRTRD	1.9	HLA-DRB1*03:01
**226**	240	LKYLEAVEKVKRTRD	1.9	HLA-DRB1*03:23
**226**	240	LKYLEAVEKVKRTRD	1.9	HLA-DRB1*03:36
**275**	289	EMPLTALLRNLGKMT	1.9	HLA-DRB1*08:06
**431**	445	QQVLMAMSQIPAGGT	1.9	HLA-DRB1*08:31
**226**	240	LKYLEAVEKVKRTRD	2.0	HLA-DRB1*03:40
**448**	462	SLPMIWAQKTNTPAD	2.0	HLA-DRB1*08:02
**448**	462	SLPMIWAQKTNTPAD	2.0	HLA-DRB1*08:09
**275**	289	EMPLTALLRNLGKMT	2.0	HLA-DRB1*11:03
**226**	240	LKYLEAVEKVKRTRD	2.0	HLA-DRB1*11:06
**226**	240	LKYLEAVEKVKRTRD	2.0	HLA-DRB1*11:25
**354**	368	DAAFYKTFKTVEPTG	2.0	HLA-DRB1*11:37
**243**	257	EVIHLIEEHRLVREH	2.1	HLA-DRB1*03:14
**310**	324	NEKLLKKARIHPFHI	2.1	HLA-DRB1*11:03
**23**	37	YVWQVTDMNRLHRFL	2.1	HLA-DRB1*11:21
**340**	354	KLKWRPDEEILKALD	2.2	HLA-DRB1*03:01
**370**	384	RFLLAVDVSASMNQR	2.2	HLA-DRB1*03:05
**340**	354	KLKWRPDEEILKALD	2.2	HLA-DRB1*03:23

**340**	354	KLKWRPDEEILKALD	2.2	HLA-DRB1*03:36
**316**	330	KARIHPFHILIALET	2.2	HLA-DRB1*08:04
**226**	240	LKYLEAVEKVKRTRD	2.2	HLA-DRB1*11:11
**334**	348	GHGLRGKLKWRPDEE	2.2	HLA-DRB1*11:11
**334**	348	GHGLRGKLKWRPDEE	2.2	HLA-DRB1*11:25
**316**	330	KARIHPFHILIALET	2.2	HLA-DRB1*14:15
**243**	257	EVIHLIEEHRLVREH	2.3	HLA-DRB1*03:05
**209**	223	VHELYKEKALSVETE	2.3	HLA-DRB1*08:03
**191**	205	SSEGLAIVTKYITKG	2.3	HLA-DRB1*08:07
**209**	223	VHELYKEKALSVETE	2.3	HLA-DRB1*08:11
**226**	240	LKYLEAVEKVKRTRD	2.3	HLA-DRB1*08:12
**209**	223	VHELYKEKALSVETE	2.3	HLA-DRB1*08:14
**23**	37	YVWQVTDMNRLHRFL	2.3	HLA-DRB1*11:02
**74**	88	IQEIKSFSQEGRTTK	2.3	HLA-DRB1*11:13
**23**	37	YVWQVTDMNRLHRFL	2.3	HLA-DRB1*11:16
**226**	240	LKYLEAVEKVKRTRD	2.4	HLA-DRB1*03:06
**474**	488	AGGVHPAIALREYRK	2.4	HLA-DRB1*04:05
**340**	354	KLKWRPDEEILKALD	2.5	HLA-DRB1*03:06
**226**	240	LKYLEAVEKVKRTRD	2.5	HLA-DRB1*03:14
**354**	368	DAAFYKTFKTVEPTG	2.5	HLA-DRB1*08:07
**191**	205	SSEGLAIVTKYITKG	2.5	HLA-DRB1*11:03
**280**	294	ALLRNLGKMTANSVL	2.5	HLA-DRB1*11:06
**354**	368	DAAFYKTFKTVEPTG	2.5	HLA-DRB1*11:08
**243**	257	EVIHLIEEHRLVREH	2.5	HLA-DRB1*11:14
**243**	257	EVIHLIEEHRLVREH	2.5	HLA-DRB1*11:20
**316**	330	KARIHPFHILIALET	2.5	HLA-DRB1*11:21
**243**	257	EVIHLIEEHRLVREH	2.6	HLA-DRB1*03:06
**420**	434	VPCPVTTDMTLQQVL	2.6	HLA-DRB1*03:06
**354**	368	DAAFYKTFKTVEPTG	2.6	HLA-DRB1*04:05
**334**	348	GHGLRGKLKWRPDEE	2.6	HLA-DRB1*11:02
**334**	348	GHGLRGKLKWRPDEE	2.6	HLA-DRB1*11:16
**191**	205	SSEGLAIVTKYITKG	2.6	HLA-DRB1*11:21
**18**	32	NSQDGYVWQVTDMNR	2.6	HLA-DRB4*01:01
**44**	58	GTYYIKEQKLGLENA	2.6	HLA-DRB4*01:01
**340**	354	KLKWRPDEEILKALD	2.7	HLA-DRB1*03:07
**275**	289	EMPLTALLRNLGKMT	2.7	HLA-DRB1*08:05
**226**	240	LKYLEAVEKVKRTRD	2.7	HLA-DRB1*08:10
**316**	330	KARIHPFHILIALET	2.7	HLA-DRB1*08:31
**431**	445	QQVLMAMSQIPAGGT	2.7	HLA-DRB1*11:04
**340**	354	KLKWRPDEEILKALD	2.7	HLA-DRB1*11:07
**316**	330	KARIHPFHILIALET	2.7	HLA-DRB1*11:13
**354**	368	DAAFYKTFKTVEPTG	2.7	HLA-DRB1*11:19
**420**	434	VPCPVTTDMTLQQVL	2.8	HLA-DRB1*03:01
**370**	384	RFLLAVDVSASMNQR	2.8	HLA-DRB1*03:07
**420**	434	VPCPVTTDMTLQQVL	2.8	HLA-DRB1*03:23
**420**	434	VPCPVTTDMTLQQVL	2.8	HLA-DRB1*03:36
**431**	445	QQVLMAMSQIPAGGT	2.8	HLA-DRB1*08:04
**354**	368	DAAFYKTFKTVEPTG	2.8	HLA-DRB1*11:01
**243**	257	EVIHLIEEHRLVREH	2.8	HLA-DRB1*11:03
**226**	240	LKYLEAVEKVKRTRD	2.8	HLA-DRB1*11:04
**370**	384	RFLLAVDVSASMNQR	2.8	HLA-DRB1*11:07

**354**	368	DAAFYKTFKTVEPTG	2.8	HLA-DRB1*11:09
**354**	368	DAAFYKTFKTVEPTG	2.8	HLA-DRB1*11:10
**74**	88	IQEIKSFSQEGRTTK	2.8	HLA-DRB1*11:14
**354**	368	DAAFYKTFKTVEPTG	2.8	HLA-DRB1*11:15
**74**	88	IQEIKSFSQEGRTTK	2.8	HLA-DRB1*11:20
**354**	368	DAAFYKTFKTVEPTG	2.8	HLA-DRB1*11:29
**431**	445	QQVLMAMSQIPAGGT	2.8	HLA-DRB1*14:15
**340**	354	KLKWRPDEEILKALD	2.9	HLA-DRB1*03:15
**370**	384	RFLLAVDVSASMNQR	2.9	HLA-DRB1*03:15
**474**	488	AGGVHPAIALREYRK	2.9	HLA-DRB1*08:03
**126**	140	FTFIQFKKDLKESMK	2.9	HLA-DRB1*08:11
**474**	488	AGGVHPAIALREYRK	2.9	HLA-DRB1*08:14
**204**	218	KGWKEVHELYKEKAL	2.9	HLA-DRB1*11:04
**431**	445	QQVLMAMSQIPAGGT	2.9	HLA-DRB1*11:06
**145**	159	GRALRKAIADWYNEK	2.9	HLA-DRB4*01:01
**474**	488	AGGVHPAIALREYRK	3.0	HLA-DRB1*08:01
**474**	488	AGGVHPAIALREYRK	3.0	HLA-DRB1*08:16
**179**	193	HKDLLRLSHLKPSSE	3.0	HLA-DRB1*08:31
**204**	218	KGWKEVHELYKEKAL	3.0	HLA-DRB1*08:31
**179**	193	HKDLLRLSHLKPSSE	3.0	HLA-DRB1*11:06
**226**	240	LKYLEAVEKVKRTRD	3.0	HLA-DRB1*11:27
**354**	368	DAAFYKTFKTVEPTG	3.0	HLA-DRB1*11:27
**462**	476	DVFIVFTDNETFAGG	3.1	HLA-DRB1*04:05
**260**	274	TNHLKSKEVWKALLQ	3.1	HLA-DRB1*08:02
**226**	240	LKYLEAVEKVKRTRD	3.1	HLA-DRB1*08:06
**209**	223	VHELYKEKALSVETE	3.1	HLA-DRB1*08:07
**260**	274	TNHLKSKEVWKALLQ	3.1	HLA-DRB1*08:09
**316**	330	KARIHPFHILIALET	3.1	HLA-DRB1*11:02
**74**	88	IQEIKSFSQEGRTTK	3.1	HLA-DRB1*11:03
**280**	294	ALLRNLGKMTANSVL	3.1	HLA-DRB1*11:04
**354**	368	DAAFYKTFKTVEPTG	3.1	HLA-DRB1*11:05
**204**	218	KGWKEVHELYKEKAL	3.1	HLA-DRB1*11:06
**316**	330	KARIHPFHILIALET	3.1	HLA-DRB1*11:16
**126**	140	FTFIQFKKDLKESMK	3.2	HLA-DRB1*08:05
**243**	257	EVIHLIEEHRLVREH	3.2	HLA-DRB1*08:12
**275**	289	EMPLTALLRNLGKMT	3.2	HLA-DRB1*11:11
**334**	348	GHGLRGKLKWRPDEE	3.2	HLA-DRB1*11:21
**126**	140	FTFIQFKKDLKESMK	3.3	HLA-DRB1*03:01
**226**	240	LKYLEAVEKVKRTRD	3.3	HLA-DRB1*03:05
**126**	140	FTFIQFKKDLKESMK	3.3	HLA-DRB1*03:23
**126**	140	FTFIQFKKDLKESMK	3.3	HLA-DRB1*03:36
**74**	88	IQEIKSFSQEGRTTK	3.3	HLA-DRB1*08:12
**275**	289	EMPLTALLRNLGKMT	3.3	HLA-DRB1*11:02
**310**	324	NEKLLKKARIHPFHI	3.3	HLA-DRB1*11:02
**179**	193	HKDLLRLSHLKPSSE	3.3	HLA-DRB1*11:04
**310**	324	NEKLLKKARIHPFHI	3.3	HLA-DRB1*11:11
**275**	289	EMPLTALLRNLGKMT	3.3	HLA-DRB1*11:16
**310**	324	NEKLLKKARIHPFHI	3.3	HLA-DRB1*11:16
**431**	445	QQVLMAMSQIPAGGT	3.3	HLA-DRB4*01:01
**7**	21	QMQPLNEKQIANSQD	3.4	HLA-DRB1*08:04
**275**	289	EMPLTALLRNLGKMT	3.4	HLA-DRB1*08:10

**316**	330	KARIHPFHILIALET	3.4	HLA-DRB1*11:25
**7**	21	QMQPLNEKQIANSQD	3.4	HLA-DRB1*14:15
**232**	246	VEKVKRTRDELEVIH	3.5	HLA-DRB1*04:05
**191**	205	SSEGLAIVTKYITKG	3.5	HLA-DRB1*08:01
**126**	140	FTFIQFKKDLKESMK	3.5	HLA-DRB1*08:04
**179**	193	HKDLLRLSHLKPSSE	3.5	HLA-DRB1*08:04
**191**	205	SSEGLAIVTKYITKG	3.5	HLA-DRB1*08:16
**226**	240	LKYLEAVEKVKRTRD	3.5	HLA-DRB1*08:31
**260**	274	TNHLKSKEVWKALLQ	3.5	HLA-DRB1*11:25
**126**	140	FTFIQFKKDLKESMK	3.5	HLA-DRB1*14:15
**179**	193	HKDLLRLSHLKPSSE	3.5	HLA-DRB1*14:15
**316**	330	KARIHPFHILIALET	3.5	HLA-DRB4*01:01
**370**	384	RFLLAVDVSASMNQR	3.6	HLA-DRB1*03:40
**354**	368	DAAFYKTFKTVEPTG	3.6	HLA-DRB1*08:02
**474**	488	AGGVHPAIALREYRK	3.6	HLA-DRB1*08:05
**354**	368	DAAFYKTFKTVEPTG	3.6	HLA-DRB1*08:09
**74**	88	IQEIKSFSQEGRTTK	3.6	HLA-DRB1*08:10
**474**	488	AGGVHPAIALREYRK	3.6	HLA-DRB1*08:11
**191**	205	SSEGLAIVTKYITKG	3.6	HLA-DRB1*11:11
**126**	140	FTFIQFKKDLKESMK	3.7	HLA-DRB1*03:06
**370**	384	RFLLAVDVSASMNQR	3.7	HLA-DRB1*03:14
**243**	257	EVIHLIEEHRLVREH	3.7	HLA-DRB1*08:10
**310**	324	NEKLLKKARIHPFHI	3.7	HLA-DRB1*11:21
**179**	193	HKDLLRLSHLKPSSE	3.7	HLA-DRB1*11:25
**179**	193	HKDLLRLSHLKPSSE	3.7	HLA-DRB4*01:01
**191**	205	SSEGLAIVTKYITKG	3.8	HLA-DRB1*08:03
**191**	205	SSEGLAIVTKYITKG	3.8	HLA-DRB1*08:14
**280**	294	ALLRNLGKMTANSVL	3.8	HLA-DRB1*08:31
**431**	445	QQVLMAMSQIPAGGT	3.8	HLA-DRB1*11:05
**448**	462	SLPMIWAQKTNTPAD	3.9	HLA-DRB1*08:11
**275**	289	EMPLTALLRNLGKMT	3.9	HLA-DRB1*08:12
**275**	289	EMPLTALLRNLGKMT	3.9	HLA-DRB1*11:21
**126**	140	FTFIQFKKDLKESMK	4.0	HLA-DRB1*03:07
**126**	140	FTFIQFKKDLKESMK	4.0	HLA-DRB1*11:07
**2**	16	EESVNQMQPLNEKQI	4.1	HLA-DRB1*04:05
**226**	240	LKYLEAVEKVKRTRD	4.1	HLA-DRB1*08:04
**191**	205	SSEGLAIVTKYITKG	4.1	HLA-DRB1*08:05
**226**	240	LKYLEAVEKVKRTRD	4.1	HLA-DRB1*11:05
**448**	462	SLPMIWAQKTNTPAD	4.1	HLA-DRB1*11:52
**226**	240	LKYLEAVEKVKRTRD	4.1	HLA-DRB1*14:15
**179**	193	HKDLLRLSHLKPSSE	4.2	HLA-DRB1*11:05
**431**	445	QQVLMAMSQIPAGGT	4.2	HLA-DRB1*11:25
**420**	434	VPCPVTTDMTLQQVL	4.3	HLA-DRB1*03:15
**407**	421	EKDSYVVAFSDEMVP	4.3	HLA-DRB1*04:05
**316**	330	KARIHPFHILIALET	4.3	HLA-DRB1*08:03
**316**	330	KARIHPFHILIALET	4.3	HLA-DRB1*08:14
**191**	205	SSEGLAIVTKYITKG	4.3	HLA-DRB1*11:14
**191**	205	SSEGLAIVTKYITKG	4.3	HLA-DRB1*11:20
**204**	218	KGWKEVHELYKEKAL	4.4	HLA-DRB1*08:04
**316**	330	KARIHPFHILIALET	4.4	HLA-DRB1*08:07
**474**	488	AGGVHPAIALREYRK	4.4	HLA-DRB1*08:31

**280**	294	ALLRNLGKMTANSVL	4.4	HLA-DRB1*11:25
**204**	218	KGWKEVHELYKEKAL	4.4	HLA-DRB1*14:15
**275**	289	EMPLTALLRNLGKMT	4.5	HLA-DRB1*08:01
**316**	330	KARIHPFHILIALET	4.5	HLA-DRB1*08:02
**448**	462	SLPMIWAQKTNTPAD	4.5	HLA-DRB1*08:07
**316**	330	KARIHPFHILIALET	4.5	HLA-DRB1*08:09
**275**	289	EMPLTALLRNLGKMT	4.5	HLA-DRB1*08:16
**316**	330	KARIHPFHILIALET	4.5	HLA-DRB1*11:06
**260**	274	TNHLKSKEVWKALLQ	4.6	HLA-DRB1*08:31
**316**	330	KARIHPFHILIALET	4.6	HLA-DRB1*11:03
**204**	218	KGWKEVHELYKEKAL	4.6	HLA-DRB1*11:25
**484**	498	REYRKKMDIPAKLIV	4.7	HLA-DRB1*08:02
**484**	498	REYRKKMDIPAKLIV	4.7	HLA-DRB1*08:09
**226**	240	LKYLEAVEKVKRTRD	4.7	HLA-DRB1*11:01
**226**	240	LKYLEAVEKVKRTRD	4.7	HLA-DRB1*11:09
**226**	240	LKYLEAVEKVKRTRD	4.7	HLA-DRB1*11:10
**226**	240	LKYLEAVEKVKRTRD	4.7	HLA-DRB1*11:15
**226**	240	LKYLEAVEKVKRTRD	4.7	HLA-DRB1*11:29
**502**	516	TSNGFTIADPDDRGM	4.8	HLA-DRB1*03:14
**474**	488	AGGVHPAIALREYRK	4.8	HLA-DRB1*08:04
**479**	493	PAIALREYRKKMDIP	4.8	HLA-DRB1*11:02
**316**	330	KARIHPFHILIALET	4.8	HLA-DRB1*11:04
**243**	257	EVIHLIEEHRLVREH	4.8	HLA-DRB1*11:11
**204**	218	KGWKEVHELYKEKAL	4.8	HLA-DRB1*11:13
**479**	493	PAIALREYRKKMDIP	4.8	HLA-DRB1*11:16
**474**	488	AGGVHPAIALREYRK	4.8	HLA-DRB1*14:15
**361**	375	FKTVEPTGKRFLLAV	4.9	HLA-DRB1*11:52
**52**	66	KLGLENAEALIRLIE	5.0	HLA-DRB1*03:07
**126**	140	FTFIQFKKDLKESMK	5.0	HLA-DRB1*03:15
**448**	462	SLPMIWAQKTNTPAD	5.0	HLA-DRB1*08:01
**209**	223	VHELYKEKALSVETE	5.0	HLA-DRB1*08:05
**316**	330	KARIHPFHILIALET	5.0	HLA-DRB1*08:06
**448**	462	SLPMIWAQKTNTPAD	5.0	HLA-DRB1*08:16
**52**	66	KLGLENAEALIRLIE	5.0	HLA-DRB1*11:07
**243**	257	EVIHLIEEHRLVREH	5.0	HLA-DRB1*11:52
**243**	257	EVIHLIEEHRLVREH	5.1	HLA-DRB1*08:07
**280**	294	ALLRNLGKMTANSVL	5.1	HLA-DRB1*11:01
**280**	294	ALLRNLGKMTANSVL	5.1	HLA-DRB1*11:09
**280**	294	ALLRNLGKMTANSVL	5.1	HLA-DRB1*11:10
**280**	294	ALLRNLGKMTANSVL	5.1	HLA-DRB1*11:15
**179**	193	HKDLLRLSHLKPSSE	5.1	HLA-DRB1*11:27
**280**	294	ALLRNLGKMTANSVL	5.1	HLA-DRB1*11:29
**95**	109	ALAICSQCSDISTKQ	5.1	HLA-DRB4*01:01
**420**	434	VPCPVTTDMTLQQVL	5.2	HLA-DRB1*03:07
**316**	330	KARIHPFHILIALET	5.2	HLA-DRB1*08:11
**126**	140	FTFIQFKKDLKESMK	5.2	HLA-DRB1*08:31
**23**	37	YVWQVTDMNRLHRFL	5.2	HLA-DRB1*11:03
**420**	434	VPCPVTTDMTLQQVL	5.2	HLA-DRB1*11:07
**275**	289	EMPLTALLRNLGKMT	5.3	HLA-DRB1*08:11
**179**	193	HKDLLRLSHLKPSSE	5.3	HLA-DRB1*11:01
**479**	493	PAIALREYRKKMDIP	5.3	HLA-DRB1*11:03

**179**	193	HKDLLRLSHLKPSSE	5.3	HLA-DRB1*11:09
**179**	193	HKDLLRLSHLKPSSE	5.3	HLA-DRB1*11:10
**179**	193	HKDLLRLSHLKPSSE	5.3	HLA-DRB1*11:15
**179**	193	HKDLLRLSHLKPSSE	5.3	HLA-DRB1*11:29
**448**	462	SLPMIWAQKTNTPAD	5.3	HLA-DRB1*11:37
**52**	66	KLGLENAEALIRLIE	5.4	HLA-DRB1*03:01
**52**	66	KLGLENAEALIRLIE	5.4	HLA-DRB1*03:23
**52**	66	KLGLENAEALIRLIE	5.4	HLA-DRB1*03:36
**280**	294	ALLRNLGKMTANSVL	5.4	HLA-DRB1*08:04
**431**	445	QQVLMAMSQIPAGGT	5.4	HLA-DRB1*11:01
**280**	294	ALLRNLGKMTANSVL	5.4	HLA-DRB1*11:05
**431**	445	QQVLMAMSQIPAGGT	5.4	HLA-DRB1*11:09
**431**	445	QQVLMAMSQIPAGGT	5.4	HLA-DRB1*11:10
**431**	445	QQVLMAMSQIPAGGT	5.4	HLA-DRB1*11:15
**126**	140	FTFIQFKKDLKESMK	5.4	HLA-DRB1*11:19
**431**	445	QQVLMAMSQIPAGGT	5.4	HLA-DRB1*11:29
**226**	240	LKYLEAVEKVKRTRD	5.4	HLA-DRB1*11:37
**280**	294	ALLRNLGKMTANSVL	5.4	HLA-DRB1*14:15
**502**	516	TSNGFTIADPDDRGM	5.5	HLA-DRB1*03:40
**243**	257	EVIHLIEEHRLVREH	5.5	HLA-DRB1*11:08
**126**	140	FTFIQFKKDLKESMK	5.5	HLA-DRB1*11:14
**243**	257	EVIHLIEEHRLVREH	5.5	HLA-DRB1*11:19
**126**	140	FTFIQFKKDLKESMK	5.5	HLA-DRB1*11:20
**474**	488	AGGVHPAIALREYRK	5.5	HLA-DRB1*11:52
**431**	445	QQVLMAMSQIPAGGT	5.6	HLA-DRB1*08:02
**260**	274	TNHLKSKEVWKALLQ	5.6	HLA-DRB1*08:06
**431**	445	QQVLMAMSQIPAGGT	5.6	HLA-DRB1*08:09
**334**	348	GHGLRGKLKWRPDEE	5.6	HLA-DRB1*11:04
**209**	223	VHELYKEKALSVETE	5.6	HLA-DRB1*11:14
**209**	223	VHELYKEKALSVETE	5.6	HLA-DRB1*11:20
**280**	294	ALLRNLGKMTANSVL	5.6	HLA-DRB1*11:27
**420**	434	VPCPVTTDMTLQQVL	5.7	HLA-DRB1*03:05
**502**	516	TSNGFTIADPDDRGM	5.7	HLA-DRB1*03:05
**74**	88	IQEIKSFSQEGRTTK	5.7	HLA-DRB1*03:15
**7**	21	QMQPLNEKQIANSQD	5.8	HLA-DRB1*08:02
**179**	193	HKDLLRLSHLKPSSE	5.8	HLA-DRB1*08:06
**7**	21	QMQPLNEKQIANSQD	5.8	HLA-DRB1*08:09
**126**	140	FTFIQFKKDLKESMK	5.9	HLA-DRB1*11:25
**18**	32	NSQDGYVWQVTDMNR	6.0	HLA-DRB1*04:05
**179**	193	HKDLLRLSHLKPSSE	6.0	HLA-DRB1*08:02
**179**	193	HKDLLRLSHLKPSSE	6.0	HLA-DRB1*08:09
**52**	66	KLGLENAEALIRLIE	6.1	HLA-DRB1*03:06
**474**	488	AGGVHPAIALREYRK	6.1	HLA-DRB1*08:07
**479**	493	PAIALREYRKKMDIP	6.1	HLA-DRB1*08:10
**204**	218	KGWKEVHELYKEKAL	6.1	HLA-DRB1*11:05
**126**	140	FTFIQFKKDLKESMK	6.1	HLA-DRB1*11:08
**334**	348	GHGLRGKLKWRPDEE	6.1	HLA-DRB1*11:14
**334**	348	GHGLRGKLKWRPDEE	6.1	HLA-DRB1*11:20
**179**	193	HKDLLRLSHLKPSSE	6.1	HLA-DRB1*11:37
**226**	240	LKYLEAVEKVKRTRD	6.2	HLA-DRB1*08:03
**226**	240	LKYLEAVEKVKRTRD	6.2	HLA-DRB1*08:14

**316**	330	KARIHPFHILIALET	6.2	HLA-DRB1*11:05
**243**	257	EVIHLIEEHRLVREH	6.2	HLA-DRB1*11:06
**316**	330	KARIHPFHILIALET	6.2	HLA-DRB1*11:14
**316**	330	KARIHPFHILIALET	6.2	HLA-DRB1*11:20
**74**	88	IQEIKSFSQEGRTTK	6.3	HLA-DRB1*03:07
**204**	218	KGWKEVHELYKEKAL	6.3	HLA-DRB1*08:06
**260**	274	TNHLKSKEVWKALLQ	6.3	HLA-DRB1*08:10
**238**	252	TRDELEVIHLIEEHR	6.3	HLA-DRB1*08:10
**238**	252	TRDELEVIHLIEEHR	6.3	HLA-DRB1*08:12
**74**	88	IQEIKSFSQEGRTTK	6.3	HLA-DRB1*11:07
**310**	324	NEKLLKKARIHPFHI	6.3	HLA-DRB1*11:25
**431**	445	QQVLMAMSQIPAGGT	6.3	HLA-DRB1*11:37
**226**	240	LKYLEAVEKVKRTRD	6.4	HLA-DRB1*08:05
**204**	218	KGWKEVHELYKEKAL	6.4	HLA-DRB1*11:01
**334**	348	GHGLRGKLKWRPDEE	6.4	HLA-DRB1*11:06
**204**	218	KGWKEVHELYKEKAL	6.4	HLA-DRB1*11:09
**204**	218	KGWKEVHELYKEKAL	6.4	HLA-DRB1*11:10
**23**	37	YVWQVTDMNRLHRFL	6.4	HLA-DRB1*11:13
**280**	294	ALLRNLGKMTANSVL	6.4	HLA-DRB1*11:13
**204**	218	KGWKEVHELYKEKAL	6.4	HLA-DRB1*11:15
**204**	218	KGWKEVHELYKEKAL	6.4	HLA-DRB1*11:29
**44**	58	GTYYIKEQKLGLENA	6.5	HLA-DRB1*03:07
**2**	16	EESVNQMQPLNEKQI	6.5	HLA-DRB1*08:06
**431**	445	QQVLMAMSQIPAGGT	6.5	HLA-DRB1*08:06
**354**	368	DAAFYKTFKTVEPTG	6.5	HLA-DRB1*08:06
**44**	58	GTYYIKEQKLGLENA	6.5	HLA-DRB1*11:07
**126**	140	FTFIQFKKDLKESMK	6.5	HLA-DRB1*11:11
**316**	330	KARIHPFHILIALET	6.5	HLA-DRB1*11:19
**502**	516	TSNGFTIADPDDRGM	6.5	HLA-DRB4*01:01
**52**	66	KLGLENAEALIRLIE	6.6	HLA-DRB1*03:15
**209**	223	VHELYKEKALSVETE	6.6	HLA-DRB1*03:40
**179**	193	HKDLLRLSHLKPSSE	6.6	HLA-DRB1*11:08
**431**	445	QQVLMAMSQIPAGGT	6.6	HLA-DRB1*11:13
**126**	140	FTFIQFKKDLKESMK	6.7	HLA-DRB1*08:02
**126**	140	FTFIQFKKDLKESMK	6.7	HLA-DRB1*08:09
**474**	488	AGGVHPAIALREYRK	6.7	HLA-DRB1*11:06
**179**	193	HKDLLRLSHLKPSSE	6.7	HLA-DRB1*11:13
**431**	445	QQVLMAMSQIPAGGT	6.7	HLA-DRB1*11:27
**462**	476	DVFIVFTDNETFAGG	6.8	HLA-DRB1*03:06
**420**	434	VPCPVTTDMTLQQVL	6.8	HLA-DRB1*03:14
**462**	476	DVFIVFTDNETFAGG	6.8	HLA-DRB1*03:15
**126**	140	FTFIQFKKDLKESMK	6.8	HLA-DRB1*11:02
**126**	140	FTFIQFKKDLKESMK	6.8	HLA-DRB1*11:16
**44**	58	GTYYIKEQKLGLENA	6.8	HLA-DRB1*11:52
**316**	330	KARIHPFHILIALET	6.8	HLA-DRB1*11:52
**209**	223	VHELYKEKALSVETE	6.9	HLA-DRB1*03:07
**260**	274	TNHLKSKEVWKALLQ	6.9	HLA-DRB1*11:03
**209**	223	VHELYKEKALSVETE	6.9	HLA-DRB1*11:07
**479**	493	PAIALREYRKKMDIP	6.9	HLA-DRB1*11:21
**238**	252	TRDELEVIHLIEEHR	6.9	HLA-DRB4*01:01
**479**	493	PAIALREYRKKMDIP	7.0	HLA-DRB1*08:12

**126**	140	FTFIQFKKDLKESMK	7.0	HLA-DRB1*11:03
**448**	462	SLPMIWAQKTNTPAD	7.0	HLA-DRB1*11:04
**316**	330	KARIHPFHILIALET	7.0	HLA-DRB1*11:11
**310**	324	NEKLLKKARIHPFHI	7.0	HLA-DRB1*11:14
**310**	324	NEKLLKKARIHPFHI	7.0	HLA-DRB1*11:20
**44**	58	GTYYIKEQKLGLENA	7.0	HLA-DRB1*11:25
**74**	88	IQEIKSFSQEGRTTK	7.1	HLA-DRB1*03:01
**44**	58	GTYYIKEQKLGLENA	7.1	HLA-DRB1*03:01
**44**	58	GTYYIKEQKLGLENA	7.1	HLA-DRB1*03:15
**74**	88	IQEIKSFSQEGRTTK	7.1	HLA-DRB1*03:23
**44**	58	GTYYIKEQKLGLENA	7.1	HLA-DRB1*03:23
**74**	88	IQEIKSFSQEGRTTK	7.1	HLA-DRB1*03:36
**44**	58	GTYYIKEQKLGLENA	7.1	HLA-DRB1*03:36
**226**	240	LKYLEAVEKVKRTRD	7.1	HLA-DRB1*08:01
**448**	462	SLPMIWAQKTNTPAD	7.1	HLA-DRB1*08:04
**226**	240	LKYLEAVEKVKRTRD	7.1	HLA-DRB1*08:16
**74**	88	IQEIKSFSQEGRTTK	7.1	HLA-DRB1*11:11
**44**	58	GTYYIKEQKLGLENA	7.1	HLA-DRB1*11:13
**74**	88	IQEIKSFSQEGRTTK	7.1	HLA-DRB1*11:19
**204**	218	KGWKEVHELYKEKAL	7.1	HLA-DRB1*11:37
**280**	294	ALLRNLGKMTANSVL	7.1	HLA-DRB1*11:37
**448**	462	SLPMIWAQKTNTPAD	7.1	HLA-DRB1*14:15
**44**	58	GTYYIKEQKLGLENA	7.2	HLA-DRB1*03:06
**179**	193	HKDLLRLSHLKPSSE	7.2	HLA-DRB1*08:12
**448**	462	SLPMIWAQKTNTPAD	7.2	HLA-DRB1*08:31
**204**	218	KGWKEVHELYKEKAL	7.2	HLA-DRB1*11:08
**361**	375	FKTVEPTGKRFLLAV	7.2	HLA-DRB1*11:13
**226**	240	LKYLEAVEKVKRTRD	7.3	HLA-DRB1*08:02
**226**	240	LKYLEAVEKVKRTRD	7.3	HLA-DRB1*08:09
**179**	193	HKDLLRLSHLKPSSE	7.3	HLA-DRB1*08:10
**431**	445	QQVLMAMSQIPAGGT	7.3	HLA-DRB1*08:10
**431**	445	QQVLMAMSQIPAGGT	7.3	HLA-DRB1*08:12
**448**	462	SLPMIWAQKTNTPAD	7.3	HLA-DRB1*11:01
**448**	462	SLPMIWAQKTNTPAD	7.3	HLA-DRB1*11:09
**448**	462	SLPMIWAQKTNTPAD	7.3	HLA-DRB1*11:10
**448**	462	SLPMIWAQKTNTPAD	7.3	HLA-DRB1*11:15
**109**	123	QAAFKAVSEVCRIPT	7.3	HLA-DRB1*11:19
**179**	193	HKDLLRLSHLKPSSE	7.3	HLA-DRB1*11:19
**448**	462	SLPMIWAQKTNTPAD	7.3	HLA-DRB1*11:29
**209**	223	VHELYKEKALSVETE	7.4	HLA-DRB1*03:06
**74**	88	IQEIKSFSQEGRTTK	7.4	HLA-DRB1*11:06
**69**	83	RGCEVIQEIKSFSQE	7.4	HLA-DRB1*11:06
**462**	476	DVFIVFTDNETFAGG	7.5	HLA-DRB1*03:01
**462**	476	DVFIVFTDNETFAGG	7.5	HLA-DRB1*03:23
**462**	476	DVFIVFTDNETFAGG	7.5	HLA-DRB1*03:36
**44**	58	GTYYIKEQKLGLENA	7.5	HLA-DRB1*11:04
**126**	140	FTFIQFKKDLKESMK	7.5	HLA-DRB1*11:21
**243**	257	EVIHLIEEHRLVREH	7.5	HLA-DRB1*11:25
**448**	462	SLPMIWAQKTNTPAD	7.5	HLA-DRB1*11:27
**260**	274	TNHLKSKEVWKALLQ	7.6	HLA-DRB1*08:11
**260**	274	TNHLKSKEVWKALLQ	7.6	HLA-DRB1*08:12

**243**	257	EVIHLIEEHRLVREH	7.6	HLA-DRB1*11:04
**448**	462	SLPMIWAQKTNTPAD	7.6	HLA-DRB1*11:05
**204**	218	KGWKEVHELYKEKAL	7.6	HLA-DRB1*11:27
**44**	58	GTYYIKEQKLGLENA	7.7	HLA-DRB1*08:31
**400**	414	CMVVTRTEKDSYVVA	7.8	HLA-DRB1*03:15
**474**	488	AGGVHPAIALREYRK	7.8	HLA-DRB1*11:04
**280**	294	ALLRNLGKMTANSVL	7.8	HLA-DRB1*11:08
**354**	368	DAAFYKTFKTVEPTG	7.8	HLA-DRB1*11:11
**74**	88	IQEIKSFSQEGRTTK	7.8	HLA-DRB1*11:25
**74**	88	IQEIKSFSQEGRTTK	7.9	HLA-DRB1*03:14
**354**	368	DAAFYKTFKTVEPTG	7.9	HLA-DRB1*03:14
**462**	476	DVFIVFTDNETFAGG	7.9	HLA-DRB1*08:03
**44**	58	GTYYIKEQKLGLENA	7.9	HLA-DRB1*08:04
**74**	88	IQEIKSFSQEGRTTK	7.9	HLA-DRB1*08:04
**238**	252	TRDELEVIHLIEEHR	7.9	HLA-DRB1*08:06
**346**	360	DEEILKALDAAFYKT	7.9	HLA-DRB1*08:12
**462**	476	DVFIVFTDNETFAGG	7.9	HLA-DRB1*08:14
**448**	462	SLPMIWAQKTNTPAD	7.9	HLA-DRB1*11:06
**23**	37	YVWQVTDMNRLHRFL	7.9	HLA-DRB1*11:14
**23**	37	YVWQVTDMNRLHRFL	7.9	HLA-DRB1*11:20
**474**	488	AGGVHPAIALREYRK	7.9	HLA-DRB1*11:25
**44**	58	GTYYIKEQKLGLENA	7.9	HLA-DRB1*14:15
**74**	88	IQEIKSFSQEGRTTK	7.9	HLA-DRB1*14:15
**209**	223	VHELYKEKALSVETE	8.0	HLA-DRB1*08:02
**474**	488	AGGVHPAIALREYRK	8.0	HLA-DRB1*08:02
**121**	135	IPTHLFTFIQFKKDL	8.0	HLA-DRB1*08:03
**448**	462	SLPMIWAQKTNTPAD	8.0	HLA-DRB1*08:03
**209**	223	VHELYKEKALSVETE	8.0	HLA-DRB1*08:09
**474**	488	AGGVHPAIALREYRK	8.0	HLA-DRB1*08:09
**121**	135	IPTHLFTFIQFKKDL	8.0	HLA-DRB1*08:14
**448**	462	SLPMIWAQKTNTPAD	8.0	HLA-DRB1*08:14
**126**	140	FTFIQFKKDLKESMK	8.0	HLA-DRB1*11:05
**448**	462	SLPMIWAQKTNTPAD	8.1	HLA-DRB1*08:05
**431**	445	QQVLMAMSQIPAGGT	8.1	HLA-DRB1*08:07
**179**	193	HKDLLRLSHLKPSSE	8.1	HLA-DRB1*11:03
**448**	462	SLPMIWAQKTNTPAD	8.1	HLA-DRB1*11:11
**204**	218	KGWKEVHELYKEKAL	8.1	HLA-DRB1*11:19
**74**	88	IQEIKSFSQEGRTTK	8.2	HLA-DRB1*03:06
**74**	88	IQEIKSFSQEGRTTK	8.2	HLA-DRB1*08:31
**280**	294	ALLRNLGKMTANSVL	8.2	HLA-DRB1*11:03
**69**	83	RGCEVIQEIKSFSQE	8.2	HLA-DRB1*11:04
**316**	330	KARIHPFHILIALET	8.2	HLA-DRB1*11:08
**361**	375	FKTVEPTGKRFLLAV	8.2	HLA-DRB1*11:21
**462**	476	DVFIVFTDNETFAGG	8.2	HLA-DRB1*11:52
**462**	476	DVFIVFTDNETFAGG	8.3	HLA-DRB1*03:05
**275**	289	EMPLTALLRNLGKMT	8.3	HLA-DRB1*11:14
**275**	289	EMPLTALLRNLGKMT	8.3	HLA-DRB1*11:20
**61**	75	LIRLIEDGRGCEVIQ	8.4	HLA-DRB1*03:01
**462**	476	DVFIVFTDNETFAGG	8.4	HLA-DRB1*03:14
**61**	75	LIRLIEDGRGCEVIQ	8.4	HLA-DRB1*03:23
**61**	75	LIRLIEDGRGCEVIQ	8.4	HLA-DRB1*03:36

**52**	66	KLGLENAEALIRLIE	8.4	HLA-DRB1*03:40
**126**	140	FTFIQFKKDLKESMK	8.4	HLA-DRB1*11:04
**260**	274	TNHLKSKEVWKALLQ	8.4	HLA-DRB1*11:06
**44**	58	GTYYIKEQKLGLENA	8.4	HLA-DRB1*11:06
**474**	488	AGGVHPAIALREYRK	8.4	HLA-DRB1*11:37
**316**	330	KARIHPFHILIALET	8.4	HLA-DRB1*11:37
**415**	429	FSDEMVPCPVTTDMT	8.5	HLA-DRB1*04:05
**74**	88	IQEIKSFSQEGRTTK	8.5	HLA-DRB1*08:06
**407**	421	EKDSYVVAFSDEMVP	8.5	HLA-DRB1*08:07
**7**	21	QMQPLNEKQIANSQD	8.5	HLA-DRB1*08:31
**69**	83	RGCEVIQEIKSFSQE	8.5	HLA-DRB1*08:31
**74**	88	IQEIKSFSQEGRTTK	8.5	HLA-DRB1*11:04
**310**	324	NEKLLKKARIHPFHI	8.5	HLA-DRB1*11:04
**260**	274	TNHLKSKEVWKALLQ	8.5	HLA-DRB1*11:04
**74**	88	IQEIKSFSQEGRTTK	8.5	HLA-DRB1*11:08
**109**	123	QAAFKAVSEVCRIPT	8.5	HLA-DRB1*11:08
**126**	140	FTFIQFKKDLKESMK	8.5	HLA-DRB1*11:27
**316**	330	KARIHPFHILIALET	8.6	HLA-DRB1*04:05
**44**	58	GTYYIKEQKLGLENA	8.6	HLA-DRB1*08:06
**243**	257	EVIHLIEEHRLVREH	8.6	HLA-DRB1*11:27
**420**	434	VPCPVTTDMTLQQVL	8.7	HLA-DRB1*03:40
**204**	218	KGWKEVHELYKEKAL	8.7	HLA-DRB1*11:52
**462**	476	DVFIVFTDNETFAGG	8.8	HLA-DRB1*03:40
**354**	368	DAAFYKTFKTVEPTG	8.8	HLA-DRB1*03:40
**484**	498	REYRKKMDIPAKLIV	8.8	HLA-DRB1*11:05
**448**	462	SLPMIWAQKTNTPAD	8.8	HLA-DRB1*11:25
**502**	516	TSNGFTIADPDDRGM	8.9	HLA-DRB1*03:06
**474**	488	AGGVHPAIALREYRK	8.9	HLA-DRB1*03:15
**179**	193	HKDLLRLSHLKPSSE	8.9	HLA-DRB1*08:05
**2**	16	EESVNQMQPLNEKQI	8.9	HLA-DRB1*08:10
**2**	16	EESVNQMQPLNEKQI	8.9	HLA-DRB1*08:12
**316**	330	KARIHPFHILIALET	8.9	HLA-DRB1*11:01
**204**	218	KGWKEVHELYKEKAL	8.9	HLA-DRB1*11:03
**316**	330	KARIHPFHILIALET	8.9	HLA-DRB1*11:09
**316**	330	KARIHPFHILIALET	8.9	HLA-DRB1*11:10
**316**	330	KARIHPFHILIALET	8.9	HLA-DRB1*11:15
**316**	330	KARIHPFHILIALET	8.9	HLA-DRB1*11:29
**249**	263	EEHRLVREHLLTNHL	8.9	HLA-DRB4*01:01
**217**	231	ALSVETEKLLKYLEA	9.0	HLA-DRB1*03:07
**462**	476	DVFIVFTDNETFAGG	9.0	HLA-DRB1*08:07
**385**	399	VLGSILNASTVAAAM	9.0	HLA-DRB1*08:31
**161**	175	GMALALAVTKYKQRN	9.0	HLA-DRB1*11:02
**217**	231	ALSVETEKLLKYLEA	9.0	HLA-DRB1*11:07
**161**	175	GMALALAVTKYKQRN	9.0	HLA-DRB1*11:16
**462**	476	DVFIVFTDNETFAGG	9.1	HLA-DRB1*03:07
**243**	257	EVIHLIEEHRLVREH	9.1	HLA-DRB1*08:06
**462**	476	DVFIVFTDNETFAGG	9.1	HLA-DRB1*11:07
**126**	140	FTFIQFKKDLKESMK	9.1	HLA-DRB1*11:37
**209**	223	VHELYKEKALSVETE	9.2	HLA-DRB1*03:01
**209**	223	VHELYKEKALSVETE	9.2	HLA-DRB1*03:14
**209**	223	VHELYKEKALSVETE	9.2	HLA-DRB1*03:23

**209**	223	VHELYKEKALSVETE	9.2	HLA-DRB1*03:36
**334**	348	GHGLRGKLKWRPDEE	9.2	HLA-DRB1*08:04
**361**	375	FKTVEPTGKRFLLAV	9.2	HLA-DRB1*11:02
**44**	58	GTYYIKEQKLGLENA	9.2	HLA-DRB1*11:03
**361**	375	FKTVEPTGKRFLLAV	9.2	HLA-DRB1*11:16
**334**	348	GHGLRGKLKWRPDEE	9.2	HLA-DRB1*14:15
**7**	21	QMQPLNEKQIANSQD	9.3	HLA-DRB1*08:06
**334**	348	GHGLRGKLKWRPDEE	9.3	HLA-DRB1*11:01
**334**	348	GHGLRGKLKWRPDEE	9.3	HLA-DRB1*11:09
**334**	348	GHGLRGKLKWRPDEE	9.3	HLA-DRB1*11:10
**462**	476	DVFIVFTDNETFAGG	9.3	HLA-DRB1*11:14
**334**	348	GHGLRGKLKWRPDEE	9.3	HLA-DRB1*11:15
**431**	445	QQVLMAMSQIPAGGT	9.3	HLA-DRB1*11:19
**462**	476	DVFIVFTDNETFAGG	9.3	HLA-DRB1*11:20
**161**	175	GMALALAVTKYKQRN	9.3	HLA-DRB1*11:21
**334**	348	GHGLRGKLKWRPDEE	9.3	HLA-DRB1*11:29
**209**	223	VHELYKEKALSVETE	9.4	HLA-DRB1*03:05
**385**	399	VLGSILNASTVAAAM	9.4	HLA-DRB1*04:05
**275**	289	EMPLTALLRNLGKMT	9.4	HLA-DRB1*08:03
**385**	399	VLGSILNASTVAAAM	9.4	HLA-DRB1*08:04
**126**	140	FTFIQFKKDLKESMK	9.4	HLA-DRB1*08:07
**275**	289	EMPLTALLRNLGKMT	9.4	HLA-DRB1*08:14
**260**	274	TNHLKSKEVWKALLQ	9.4	HLA-DRB1*11:02
**126**	140	FTFIQFKKDLKESMK	9.4	HLA-DRB1*11:06
**161**	175	GMALALAVTKYKQRN	9.4	HLA-DRB1*11:06
**431**	445	QQVLMAMSQIPAGGT	9.4	HLA-DRB1*11:08
**69**	83	RGCEVIQEIKSFSQE	9.4	HLA-DRB1*11:13
**260**	274	TNHLKSKEVWKALLQ	9.4	HLA-DRB1*11:16
**385**	399	VLGSILNASTVAAAM	9.4	HLA-DRB1*14:15
**209**	223	VHELYKEKALSVETE	9.5	HLA-DRB1*03:15
**217**	231	ALSVETEKLLKYLEA	9.5	HLA-DRB1*03:15
**502**	516	TSNGFTIADPDDRGM	9.5	HLA-DRB1*03:15
**2**	16	EESVNQMQPLNEKQI	9.5	HLA-DRB1*08:05
**204**	218	KGWKEVHELYKEKAL	9.5	HLA-DRB1*08:05
**479**	493	PAIALREYRKKMDIP	9.5	HLA-DRB1*08:06
**448**	462	SLPMIWAQKTNTPAD	9.5	HLA-DRB1*11:13
**52**	66	KLGLENAEALIRLIE	9.6	HLA-DRB1*03:05
**69**	83	RGCEVIQEIKSFSQE	9.6	HLA-DRB1*03:07
**23**	37	YVWQVTDMNRLHRFL	9.6	HLA-DRB1*03:15
**385**	399	VLGSILNASTVAAAM	9.6	HLA-DRB1*08:02
**346**	360	DEEILKALDAAFYKT	9.6	HLA-DRB1*08:07
**385**	399	VLGSILNASTVAAAM	9.6	HLA-DRB1*08:09
**69**	83	RGCEVIQEIKSFSQE	9.6	HLA-DRB1*11:07
**7**	21	QMQPLNEKQIANSQD	9.6	HLA-DRB1*11:25
**431**	445	QQVLMAMSQIPAGGT	9.6	HLA-DRB1*11:52
**23**	37	YVWQVTDMNRLHRFL	9.7	HLA-DRB1*03:07
**69**	83	RGCEVIQEIKSFSQE	9.7	HLA-DRB1*03:15
**23**	37	YVWQVTDMNRLHRFL	9.7	HLA-DRB1*11:07
**44**	58	GTYYIKEQKLGLENA	9.7	HLA-DRB1*11:21
**161**	175	GMALALAVTKYKQRN	9.7	HLA-DRB1*11:25
**400**	414	CMVVTRTEKDSYVVA	9.8	HLA-DRB1*03:07

**316**	330	KARIHPFHILIALET	9.8	HLA-DRB1*08:01
**316**	330	KARIHPFHILIALET	9.8	HLA-DRB1*08:16
**126**	140	FTFIQFKKDLKESMK	9.8	HLA-DRB1*11:01
**361**	375	FKTVEPTGKRFLLAV	9.8	HLA-DRB1*11:03
**310**	324	NEKLLKKARIHPFHI	9.8	HLA-DRB1*11:06
**400**	414	CMVVTRTEKDSYVVA	9.8	HLA-DRB1*11:07
**126**	140	FTFIQFKKDLKESMK	9.8	HLA-DRB1*11:09
**126**	140	FTFIQFKKDLKESMK	9.8	HLA-DRB1*11:10
**126**	140	FTFIQFKKDLKESMK	9.8	HLA-DRB1*11:15
**126**	140	FTFIQFKKDLKESMK	9.8	HLA-DRB1*11:29
**502**	516	TSNGFTIADPDDRGM	9.9	HLA-DRB1*03:01
**502**	516	TSNGFTIADPDDRGM	9.9	HLA-DRB1*03:23
**502**	516	TSNGFTIADPDDRGM	9.9	HLA-DRB1*03:36
**74**	88	IQEIKSFSQEGRTTK	9.9	HLA-DRB1*03:40
**260**	274	TNHLKSKEVWKALLQ	9.9	HLA-DRB1*11:05
**479**	493	PAIALREYRKKMDIP	9.9	HLA-DRB1*11:11
**385**	399	VLGSILNASTVAAAM	9.9	HLA-DRB1*11:52
**474**	488	AGGVHPAIALREYRK	10.0	HLA-DRB1*03:06
**260**	274	TNHLKSKEVWKALLQ	10.0	HLA-DRB1*08:01
**204**	218	KGWKEVHELYKEKAL	10.0	HLA-DRB1*08:02
**204**	218	KGWKEVHELYKEKAL	10.0	HLA-DRB1*08:09
**260**	274	TNHLKSKEVWKALLQ	10.0	HLA-DRB1*08:16
**44**	58	GTYYIKEQKLGLENA	10.0	HLA-DRB1*11:02
**44**	58	GTYYIKEQKLGLENA	10.0	HLA-DRB1*11:16
**474**	488	AGGVHPAIALREYRK	10.0	HLA-DRB4*01:01
