# Supplementary material for: Predicted T-Cell and B-Cell Epitopes of NIS: Where Do Sjögren’s Syndrome and Hashimoto’s Thyroiditis Converge?
Source: Int J Mol Sci. 2025 Dec 24;27(1):200. doi: 10.3390/ijms27010200 (PMC12785876; doi:10.3390/ijms27010200)
Supplement: Supplementary file 1 [file ijms-27-00200-s001.zip › Table S6 IJMS.pdf]

Rank	Sequence	Start position	Score
**1**	VHPAIALREYRKKMDI	477	0.91
**1**	ASTVAAAMCMVVTRTE	392	0.91
**2**	DMCGFDTGALDVIRNF	518	0.88
**2**	NGFTIADPDDRGMLDM	504	0.88
**2**	MCMVVTRTEKDSYVVA	399	0.88
**3**	GKLKWRPDEEILKALD	339	0.86
**3**	EKLCNEKLLKKARIHP	306	0.86
**4**	PAGGTDCSLPMIWAQK	441	0.85
**4**	QQVLMAMSQIPAGGTD	431	0.85
**5**	CFGSEGGTYYIKEQKL	38	0.84
**5**	TKYITKGWKEVHELYK	199	0.84
**6**	CEVIQEIKSFSQEGRT	71	0.83
**7**	DEMVPCPVTTDMTLQQ	417	0.82
**7**	KQIANSQDGYVWQVTD	14	0.82
**8**	IHLIEEHRLVREHLLT	245	0.81
**9**	PGNSEVSLVCEKLCNE	296	0.80
**9**	KESMKCGMWGRALRKA	136	0.80
**10**	PVTTDMTLQQVLMAMS	423	0.78
**10**	VSASMNQRVLGSILNA	377	0.78
**10**	TNHLKSKEVWKALLQE	260	0.78
**11**	IVCGMTSNGFTIADPD	497	0.77
**11**	QKTNTPADVFIVFTDN	455	0.77
**11**	SVNQMQPLNEKQIANS	4	0.77
**11**	KAIADWYNEKGGMALA	150	0.77
**12**	IRLIEDGRGCEVIQEI	62	0.76
**12**	FIVFTDNETFAGGVHP	464	0.76
**12**	LPMIWAQKTNTPADVF	449	0.76
**12**	LETYKTGHGLRGKLKW	328	0.76
**12**	ISTKQAAFKAVSEVCR	105	0.76
**13**	YIKEQKLGLENAEALI	47	0.75

**13**	LGSILNASTVAAAMCM	386	0.75
**13**	DEEILKALDAAFYKTF	346	0.75
**13**	NGWSHKDLLRLSHLKP	175	0.75
**14**	KMTANSVLEPGNSEVS	287	0.74
**14**	PTHLFTFIQFKKDLKE	122	0.74
**15**	GMWGRALRKAIADWYN	142	0.73
**16**	QDGYVWQVTDMNRLHR	20	0.71
**17**	DSYVVAFSDEMVPCPV	409	0.70
**17**	HRLVREHLLTNHLKSK	251	0.70
**18**	SFSQEGRTTKQEPMLF	79	0.69
**18**	TKYKQRNGWSHKDLLR	169	0.69
**19**	LSHLKPSSEGLAIVTK	185	0.68
**20**	KKMDIPAKLIVCGMTS	488	0.67
**21**	RTTKQEPMLFALAICS	85	0.66
**22**	EAVEKVKRTRDELEVI	230	0.65
**23**	FKTVEPTGKRFLLAVD	361	0.63
**24**	LKKARIHPFHILIALE	314	0.61
**24**	TEKLLKYLEAVEKVKR	222	0.61
**25**	QEMPLTALLRNLGKMT	274	0.60
**26**	GKRFLLAVDVSASMNQ	368	0.58
**26**	KEVWKALLQEMPLTAL	266	0.58
**26**	WKEVHELYKEKALSVE	206	0.58
**27**	NEKGGMALALAVTKYK	157	0.56
