# Supplementary material for: Predicted T-Cell and B-Cell Epitopes of NIS: Where Do Sjögren’s Syndrome and Hashimoto’s Thyroiditis Converge?
Source: Int J Mol Sci. 2025 Dec 24;27(1):200. doi: 10.3390/ijms27010200 (PMC12785876; doi:10.3390/ijms27010200)
Supplement: Supplementary file 1 [file ijms-27-00200-s001.zip › Table S7 IJMS.pdf]

Chain ID	Residue ID	Residue name	Contact number	Propensity score	DiscoTope score
**A**	13	GLU	0	1.892	1.674
**A**	1	MET	6	1.166	0.342
**A**	2	GLU	8	0.907	-0.118
**A**	12	ASN	12	0.928	-0.559
**A**	340	LYS	0	-0.64	-0.567
**A**	3	GLU	7	-0.541	-1.284
**A**	10	PRO	9	-0.782	-1.727
**A**	339	GLY	7	-1.068	-1.75
**A**	9	GLN	8	-1.292	-2.063
**A**	239	ARG	0	-2.481	-2.195
**A**	338	ARG	3	-2.117	-2.218
**A**	4	SER	16	-0.691	-2.452
**A**	491	ASP	0	-3.006	-2.661
**A**	14	LYS	13	-1.441	-2.771
**A**	21	ASP	2	-3.152	-3.019
**A**	5	VAL	15	-1.537	-3.085
**A**	488	LYS	7	-2.639	-3.14
**A**	240	ASP	7	-3.156	-3.598
