# Supplementary figures and images for: Predicted T-Cell and B-Cell Epitopes of NIS: Where Do Sjögren’s Syndrome and Hashimoto’s Thyroiditis Converge?
Source: Int J Mol Sci. 2025 Dec 24;27(1):200. doi: 10.3390/ijms27010200 (PMC12785876; doi:10.3390/ijms27010200)

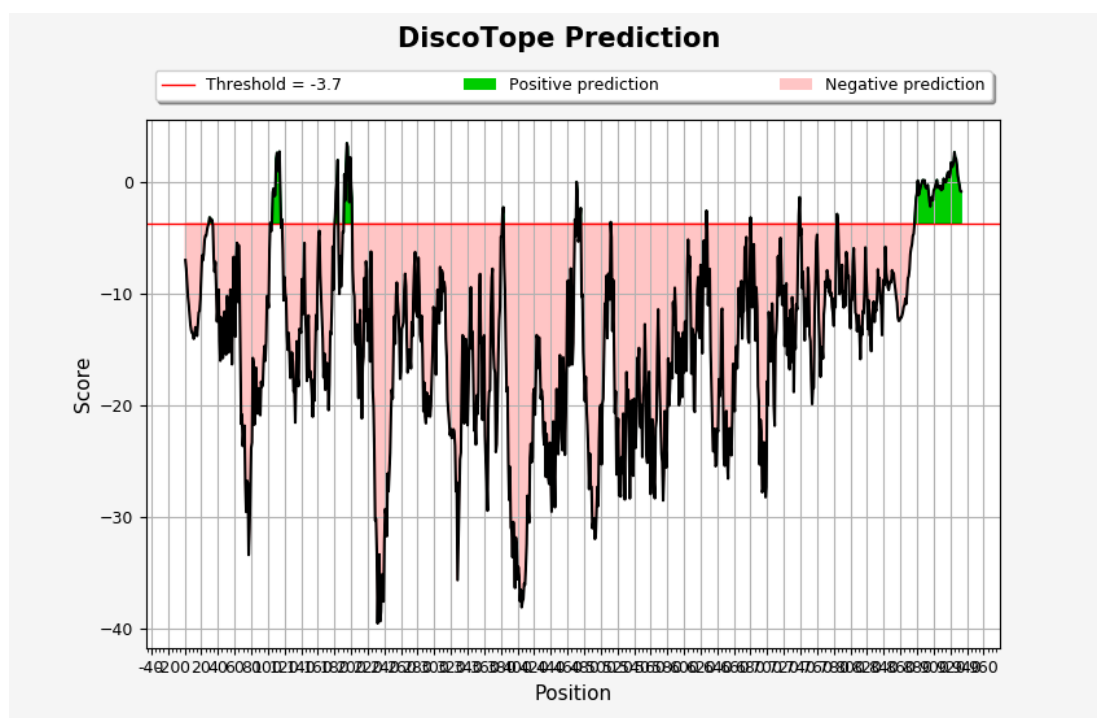

a

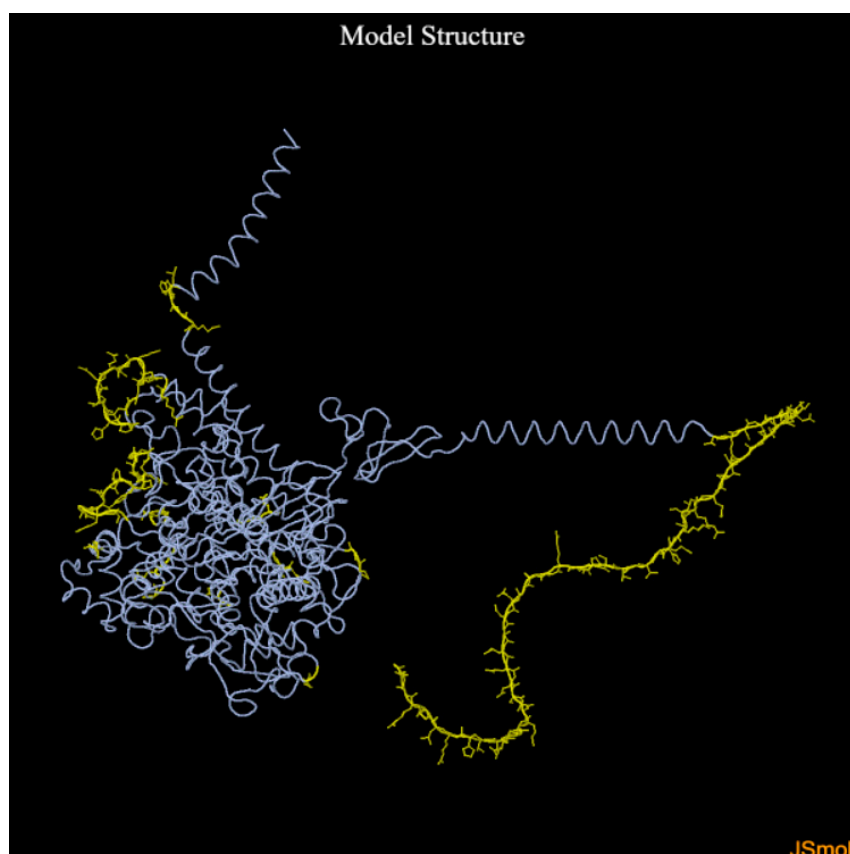

b

Supplement: Supplementary file 1 [file ijms-27-00200-s001.zip › Figure S1 Cammaroto (1).pdf]

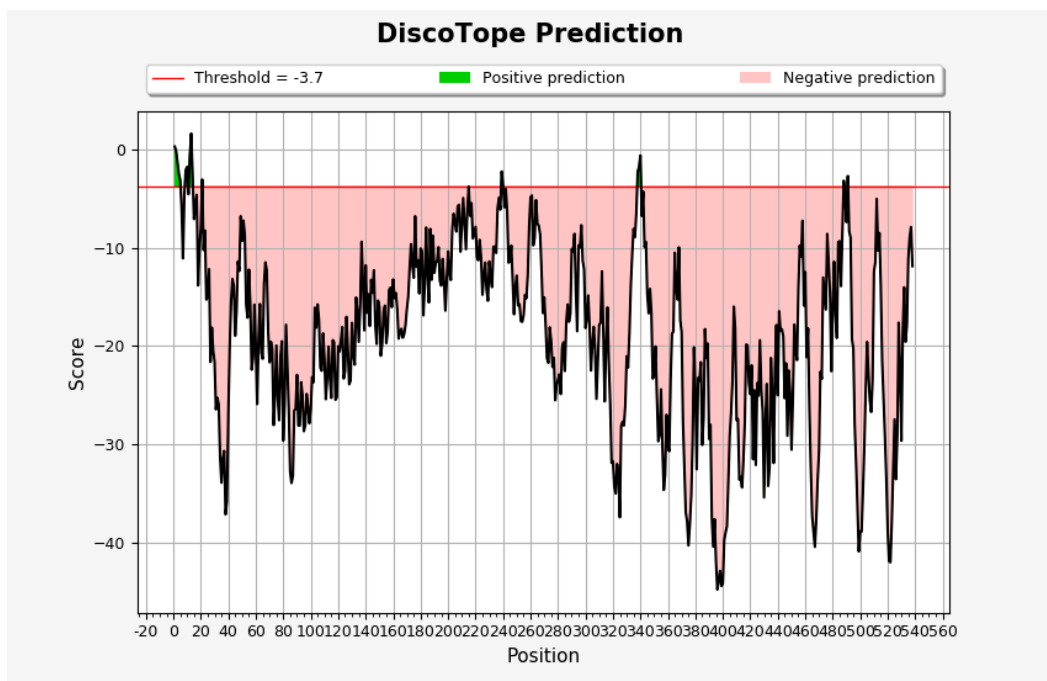

a

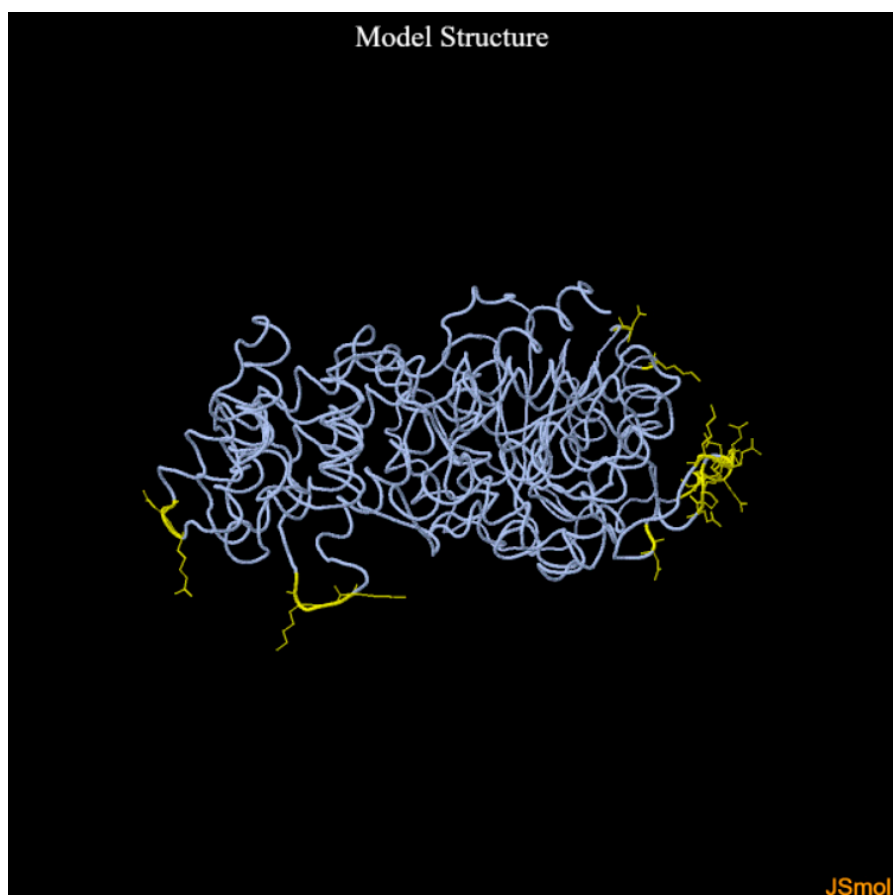

b

Supplement: Supplementary file 1 [file ijms-27-00200-s001.zip › Figure S2 Cammaroto.pdf]
